# Donor–Acceptor-Substituted 5‑Azaazulenes

**DOI:** 10.1021/acs.joc.5c01663

**Published:** 2025-10-17

**Authors:** Enikő Meiszter, Gábor Turczel, András Stirling, Péter Pál Fehér, Gábor London

**Affiliations:** 1 Institute of Organic Chemistry, 579839 HUN-REN Research Centre for Natural Sciences, 1117 Budapest, Hungary; 2 Department of Organic Chemistry and Technology, Faculty of Chemical Technology and Biotechnology, Budapest University of Technology and Economics, H-1111 Budapest, Hungary; 3 NMR Research Laboratory, Centre for Structural Science, HUN-REN Research Centre for Natural Sciences, H-1117 Budapest, Hungary; 4 Eszterházy Károly Catholic University, Leányka u. 6, H-3300 Eger, Hungary

## Abstract

The synthesis of 5-azaazulenes with both donor and acceptor substituents on their seven-membered rings is reported through the ring expansion of stable azapentalene derivatives upon reaction with dimethyl acetylenedicarboxylate. Regioisomeric products were obtained and characterized. The mechanism of the transformation and the excited state energy levels of the products were studied computationally, suggesting that these structures can be entry points to chromophore design for organic photonics applications.

## Graphical abstract

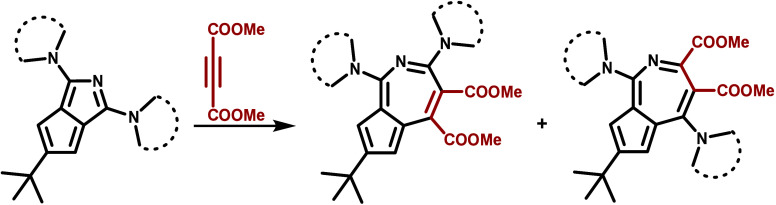

Conjugated azaheterocycles recently became attractive starting points for the design of organic emitters due to their potential to exhibit inverted singlet–triplet (IST) gaps.^1^ Currently, the number of available IST chromophores is limited; hence, research efforts focus on exploring design principles to expand the scope of promising scaffolds.^2^ This led to a deeper understanding of the relationships between molecular structure, energetics, and properties. Notably, development is often based on computational screening of large compound libraries, which is an efficient approach to quickly discover molecules with specific structural and electronic features and to derive design principles from these features.^3^ Analysis of the synthetic accessibility of computed scaffolds must follow virtual screening.^4^ Recent computational studies on IST molecules considered azaazulenes—azulene^5^ derivatives with incorporated nitrogen atom(s)—as a potential scaffold for development.^2g,3b,3e^ Inspired by these studies, we aimed to explore the relevant chemical space synthetically. Considering their π-electron perimeter for nitrogen atom incorporation and the diverse functional group patterns that could be envisioned, azaazulenes offer great structural diversity. Nevertheless, synthetic studies have focused mostly on 1-azaazaulene derivatives.^6^ From the point of photonics applications, the computationally proposed molecules^3b^ exhibited features that were different from reported 1-azaazulenes. The generated library of molecules contained azaazulene cores with nitrogen atom(s) and substituents as part of the seven-membered ring. While most of the computed structures had multiple nitrogen atoms within the core, access to azaazulenes having both nitrogen atom(s) and substituent(s) as part of the seven-membered ring is challenging in general.^6^ Based on the patterns from the computational screening, we focused our attention on 5-azaazulenes with functional groups on the seven-membered ring as an entry to the chromophore design. From the few available reports on the synthesis of 5-azaazulenes,^7^ particularly interesting is the approach that converts stable azapentalenes, which are potential IST chromophores themselves,^2e^ to substituted 5-azaazulenes in a technically simple, ring-expansion transformation.^7c^ Initially described by Hafner and co-workers, this transformation between azapentalene **1** and dimethyl acetylenedicarboxylate (DMAD) leads to substituted 5-azaazulene **2**, among other products, depending on the reaction conditions (Figure 1a). While a lower excess of DMAD and lower temperature favor the formation of the 5-azaazulene derivative, higher temperature and excess electrophilic reagent lead to a product mixture.^7c,7e^ The composition of the inseparable mixture suggests that the initial azaazulene product reacts further with DMAD under the latter conditions.

**Figure 1:**
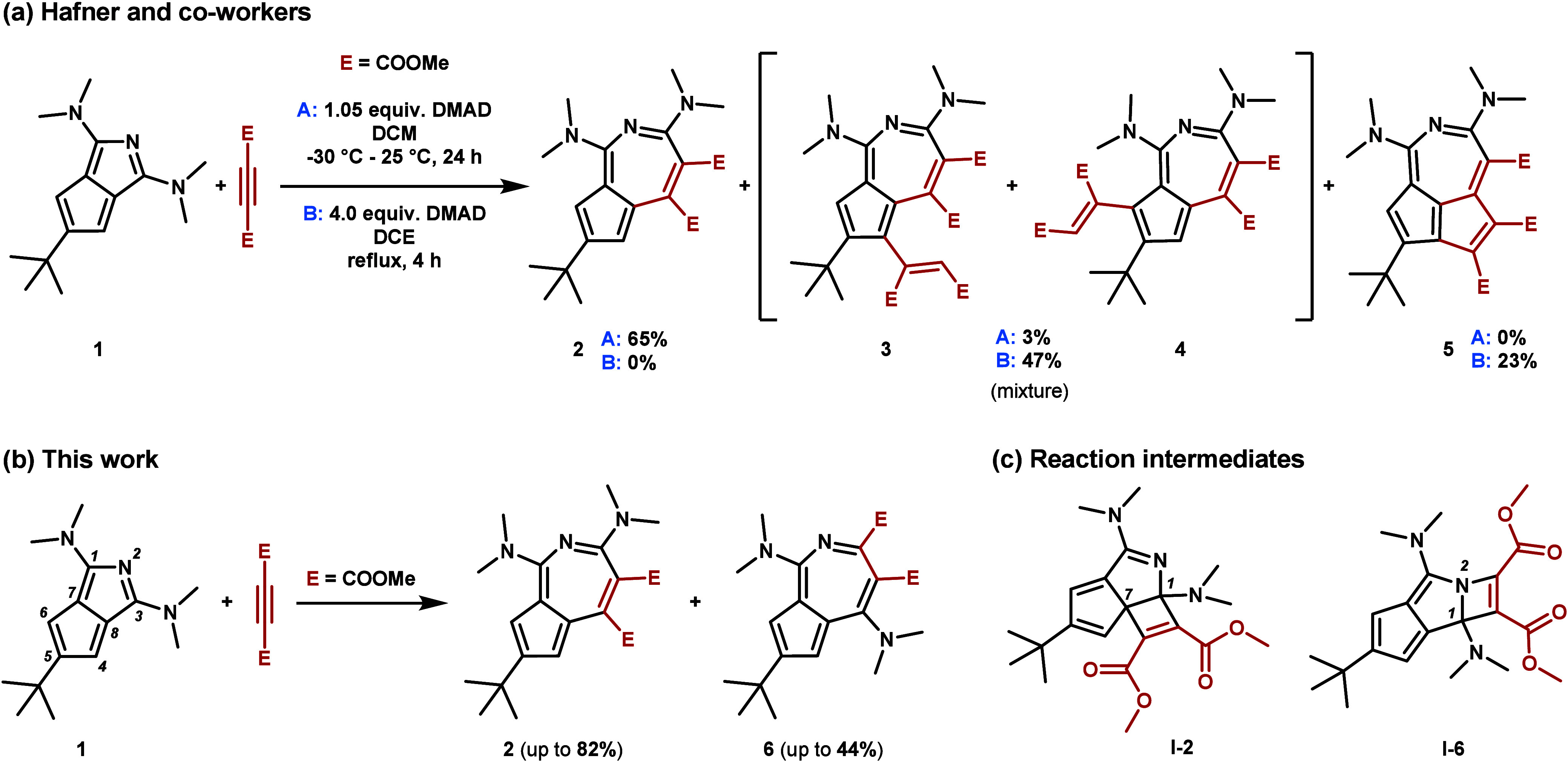
(a) Products from the reaction of azapentalene **1** and DMAD under different conditions as reported by Hafner and co-workers. (b) The formation of regioisomeric azaazulenes described in this work. (c) Calculated structures of intermediates leading to isomeric **2** and **6**.

In contrast to the reported results, we found that in DCM at 0 °C a mixture of regioisomeric products **2** and **6** can be separated and characterized (Figure 1b, c). Moreover, one of the isomers could be obtained selectively by tuning the reaction conditions. The effect of temperature in DCM was negligible; performing the reaction at 25 °C led to a similar outcome. Other species could be detected only in traces under these conditions. The product distribution showed strong solvent dependence (Table 1). The yield of isomer **6** increased in more polar solvents, while in those cases the reactions were somewhat faster and the overall yields were higher. In CH_3_CN the ratio of isomers **2** and **6** was about 1:1, which made the characterization of both isomers possible. In DMF and DMSO compound **2** was the favored isomer.

**Table 1:** Effect of Reaction Conditions on the Formation of Isomeric Azaazulenes **2** and **6**

				Yield (%)^b^	
Entry	Solvent	Temp (°C)	Time (h)^a^	**2**	**6**
1	DCM	0 → 25	17	48	<1
2	DCE	0 → 25	4.5	46	12
3	CH_3_CN	0 → 25	6.5	45	44
4	DMF	0 → 25	5	55	25
5	DMSO	0 → 25	4	74	10
6	DMF	0 → 50	3.5	82	<1
7	toluene	25 → 50	24	33	<1
8	MeOH	0 → 25	4.5	formation of **F1** (67%)	

a Time needed for full conversion of **1**.

b Isolated yields.

Notably, higher temperature in DMF provided compound **2** selectively in 82% yield. In toluene, product formation was slow at rt, which could be improved at higher temperature, although the yield remained low even after a long reaction time. Compound **2** formed selectively in this case, similar to what was found in DCM. The transformation also proceeded upon the exclusion of light with similar results. In MeOH, azaazulenes were not isolated; instead, fulvene derivatives (**F1** and **F2**, Figure 2a) were obtained in good yields (for comprehensive NMR spectroscopic analysis, see Supporting Information). Notably, these products did not form upon the treatment of azaazulenes with MeOH, which rules out a ring-opening pathway by MeOH for their formation and suggests the interruption of the ring expansion process by the solvent.

**Figure 2:**
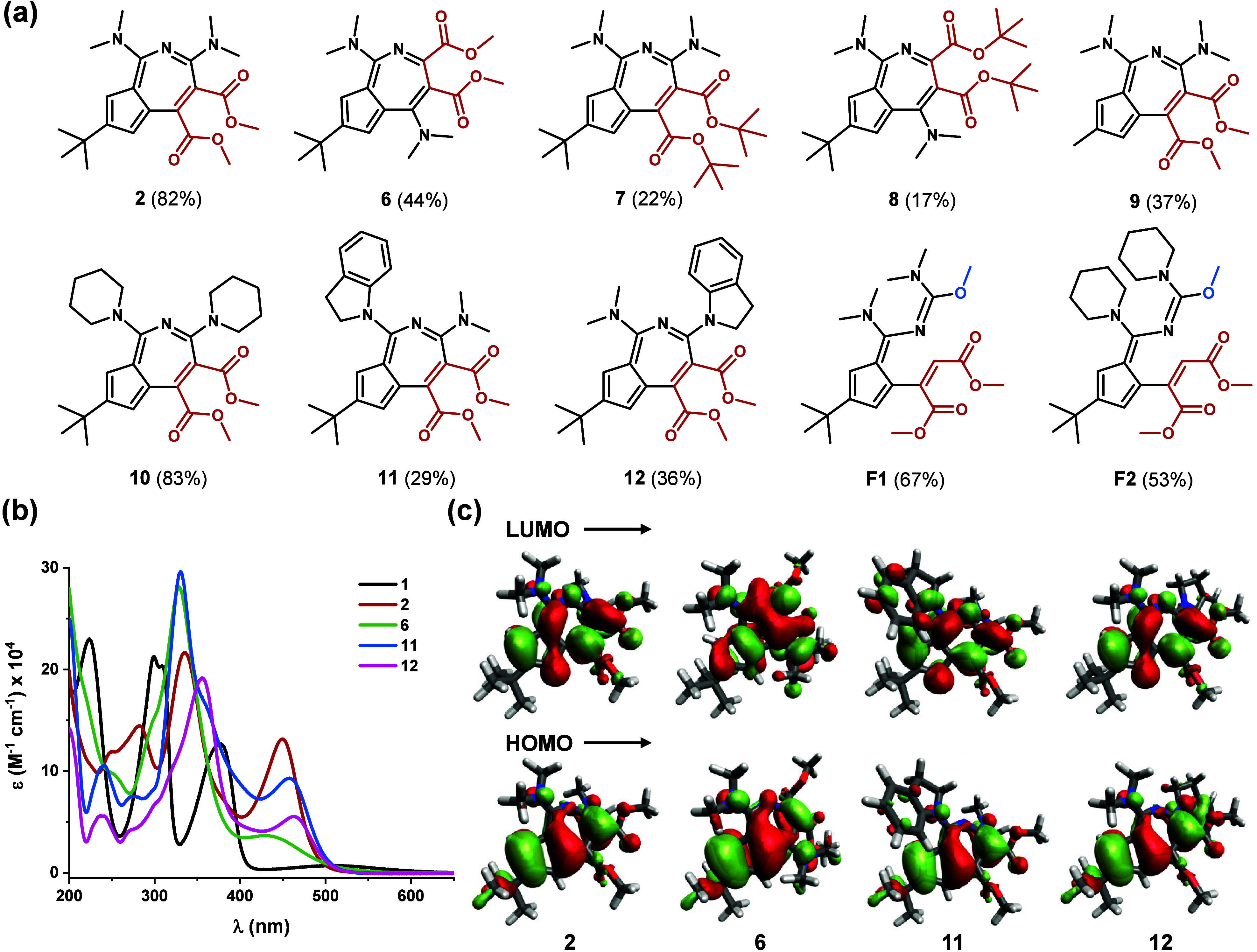
(a) Structures of the isolated compounds. (b) UV–vis spectra of selected products (CH_3_CN, rt). (c) Calculated HOMOs and LUMOs of compounds **2**, **6**, **11** and **12**.

DFT investigation of the ring expansion mechanism revealed that an alkyne carbon of DMAD can attack either the C7 or N2 atoms of azapentalene (Scheme 1). It is then followed by a ring closure to afford a four-membered ring via bond formation between the other alkyne carbon and C1. Depending on whether C7 or N2 is attacked first, this leads to intermediates **I-2** and **I-6**, respectively. Products **2** and **6** are then obtained through the intramolecular rearrangement of the condensed 5- and 4-membered rings into a 7-membered ring to afford the azaazulene core. The rate-determining step for the process is the formation of the 4-membered ring (ΔG^‡^ = 27.7 and 29.6 kcal mol^–1^ for **I-2** and **I-6**, respectively) with the selectivity toward **2** already determined at the initial C7 attack (for the energy profile of both pathways, see section S6 in the Supporting Information).

**Scheme 1:**
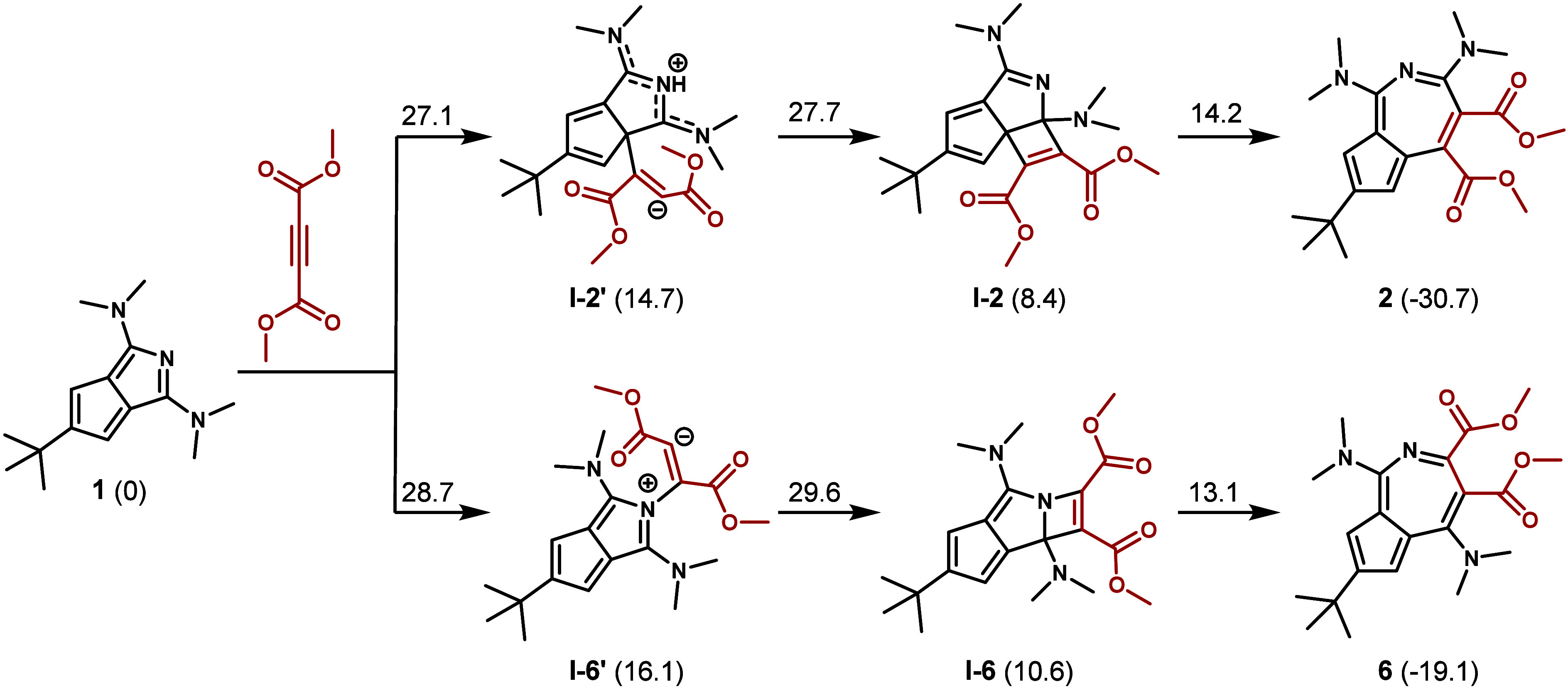
Computed Mechanism of the Reaction between Azapentalene **1** and DMAD Leading to Regioisomeric Azaazulenes **2** and **6** ^a^

a Values in parentheses below the structures and on the arrows are the relative free energies in kcal mol^–1^ of the corresponding structures and the transition states connecting them, respectively. For computational details and detailed energy profiles see the Supporting Information.

The structure of both reaction partners was varied to explore the scope and limitations of the transformation in DMF (Figure 2a; see also section S3 in the Supporting Information). The reaction was found to be sensitive to the structures of both reagents. Replacement of the ester groups of DMAD with alkyl or aryl substituents led to mixtures of unstable products. The reaction did not occur with acetylenedicarboxylic acid or with its disodium salt, while a bicyclononyne derivative led to poor conversion. Using di-*tert*-butyl acetylenedicarboxylate provided isomers **7** and **8** in lower yield, likely due to the bulky *t*Bu groups.

The reactivity of a series of available^8^ azapentalene derivatives were also explored. Azapentalenes with different C5 substituents provided product in the case of the 5-methyl derivative (**S1**), while those with bromine, azophenyl, or formyl groups were not reactive. Reactants with different amine substituents on the heterocyclic ring of azapentalene were tolerated. Both the bispiperidine (**S2**) and the monodimethylamine monoindoline (**S3**) derivative were reactive and led to stable products. The transformation was selective for piperidine substituted **10**, while it gave an isomeric product mixture of **11** and **12** when different amines were present in the starting azapentalene. The latter isomers could be separated and characterized. Notably, the formation of **11** and **12** were facilitated by the addition of K_2_CO_3_ (see also section S4 in the Supporting Information). The effect of the base is unclear, but it is likely that it neutralizes the (partially) hydrolyzed DMAD reagent that could negatively affect the reaction in this case. Postsynthetic modifications of azaazulene **2**, including ester hydrolysis, reduction and amine substitution, were attempted, but no stable products could be isolated (see also section S3 in the Supporting Information).

In their UV–vis spectra (Figure 2b), the main absorption bands of azaazulenes **2** (335 and 450 nm), **6** (330 and 435 nm), **11** (330 and 458 nm) and **12** (355 and 463 nm) are red-shifted compared to those of the parent azapentalene **1** (300 and 376 nm). These could be assigned as HOMO → LUMO and HOMO–1 → LUMO transitions (Tables S2–S5 in the Supporting Information). The isolated molecules did not show any apparent fluorescence. For isomers **2** and **6** both the first oxidation and reduction processes were irreversible based on cyclic voltammetry measurements (see section S5 in the Supporting Information).

The excited state properties and potential IST behavior of the synthesized azaazulenes were also evaluated. Most computational studies suggest that symmetry is a required structural design element for IST materials;^1b,2c,3d^ however, there are recent reports that debate the role of molecular symmetry.^3c,2h^ Therefore, the search for nonsymmetric IST molecules can be highly profitable. Figure 2c shows that the HOMO orbitals of the different azaazulenes are similar and are dominated by a cyclopentadiene-type arrangement on the 5-membered carbocycle. Regarding their LUMOs, **2**, **11** and **12** show similarities due to the presence of a pentafulvene type pattern, which is not apparent in the LUMO of **6**. Overall, however, the HOMO and LUMO orbital pairs for the studied azaazulenes are very similar, which is generally undesirable for IST candidates because for transitions between states of similar orbital characteristics the quantum mechanical exchange integral becomes large and leads to triplet stabilization and destabilization of singlet excited states.^9^ In any case, it is instructive to further examine the excited state properties of representative synthesized systems (Figure 3). The Tamm–Dancoff Approximation (TDA)-DFT calculated excitation energies indicate that compounds **2**, **6**, **11** and **12** obey Hund’s rule based on their S_1_ and T_1_ energies; however, higher-lying Hunds’s rule violation [E­(S_*x*_-T_*y*_) < 0] ^3d,10^ is predicted for **2** (S_2_-T_4_, S_3_-T_4_), **11** (S_1_-T_3_, S_3_-T_4_, S_4_-T_4_) and **12** (S_4_-T_5_). Hund’s rule violations in the higher excited states are already being considered as entry points for further structural tuning toward negative ΔE­(S_1_–T_1_),^10^ and hence practically useful emitters.

**Figure 3:**
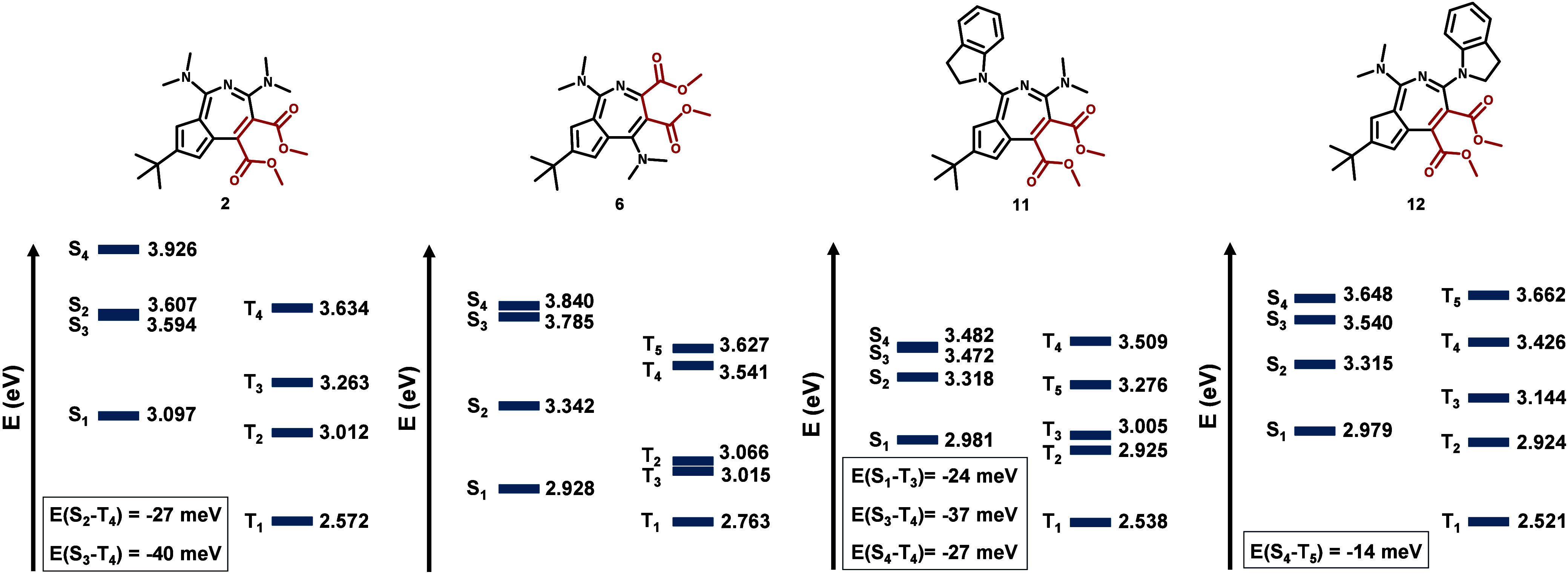
Excited-state energy diagrams of compounds **2**, **6**, **11** and **12**.

Hole–electron analysis was also performed to analyze the nature of the excitations (for further details, see section S6 in the Supporting Information). This reinforced the previous findings; i.e., there is a high degree of similarity between the S_1_ and T_1_ states. The only notable exception is **6**, where the hole of the S_1_ state contains a significant (27%) amount of HOMO–1 that is not present in T_1_. HOMO–1 has slightly different characteristics, as it is centered mainly on the 5-membered ring and has a rotated (relative to the HOMO) cyclopentadiene-type arrangement, while HOMO and LUMO extend to the whole azaazulene core (Figure S9). This indicates that functionalization of **6** at the C4 or C6 sites could influence the ΔE­(S_1_–T_1_). Considering the hole–electron distances and overlaps, all of the synthesized compounds feature mostly local excitations (for further details, see section S6 in the Supporting Information). For compound **2** the largest hole–electron separation is in the S_4_ (1.9 Å), while for **12** it is in the S_3_ (1.6 Å) state, indicating that charge transfer barely exceeds a single C–C bond distance.

In summary, we disclosed the synthesis of substituted 5-azaazulene derivatives through a ring expansion reaction of stable azapentalenes. In contrast to previous reports, the process led to regioisomeric azaazulene products that we have isolated, characterized, and evaluated their potential as IST chromophores. Although the synthesized set of compounds did not show inversion of the low-lying singlet and triplet levels based on computations, higher-lying Hund’s rule violation was observed for some derivatives. This type of behavior has been considered as a starting point for further structural tuning toward applications.^10^

## Supplementary Material



## Data Availability

The data underlying this study are available in the published article and its Supporting Information.

## Notes

Detailed synthetic procedures for starting materials and products and further data on scope and limitations of the transformation, and on attempted postsynthetic modifications of compound **2**. Detailed procedures and results of cyclic voltammetry measurements on **2** and **6**. Detailed procedures for theoretical characterization and Cartesian coordinates of calculated structures. Images of NMR spectra and HRMS spectra of the synthesized compounds. (PDF)
