# Supplementary material for: Donor–Acceptor-Substituted 5‑Azaazulenes
Source: J Org Chem. 2025 Oct 17;90(43):15381–6. doi: 10.1021/acs.joc.5c01663 (PMC12584107; doi:10.1021/acs.joc.5c01663)
Supplement: Supplementary file 1 [file jo5c01663_si_001.pdf]

Supplementary Information

Donor-acceptor-substituted 5-azaazulenes

Enikő Meiszter[1,2], Gábor Turczel[3], András Stirling[1,4,*], Péter Pál Fehér[1,*], Gábor London[1,*]

[1]Institute of Organic Chemistry, HUN-REN Research Centre for Natural Sciences, 1117 Budapest, Hungary

[2]Department of Organic Chemistry and Technology, Faculty of Chemical Technology and Biotechnology, Budapest University of Technology and Economics, H-1111 Budapest, Hungary

[3]NMR Research Laboratory, Centre for Structural Science, HUN-REN Research Centre for Natural Sciences, H-1117 Budapest, Hungary

[4]Eszterházy Károly Catholic University, Leányka u. 6, H-3300 Eger, Hungary

Table of Contents

S1 General Information 3

S2 Syntheses 5

S3 Scope and limitations and attempted post-synthetic modifications 14

S4 Effect of bases 17

S5 Electrochemistry 18

S6 Computational details 22

S7 NMR spectra 33

S8 HRMS spectra 49

S9 Cartesian coordinates and free energies in XYZ format 56

S10 References 66

S1 General Information

Reagents, Solvents, Instrumentation and Chromatography. Commercial reagents, solvents, and catalysts (Sigma-Aldrich, Fluorochem, VWR) were purchased as reagent grade and used without further purification. Solvents for extraction or column chromatography were of technical quality. Organic solutions were concentrated by rotary evaporation at 40 °C. The microwave reactions were carried out in an Anton Paar microwave synthesizer reactor type Microwave300 in sealed reaction vessels. Thin-layer chromatography (TLC) was carried out on 'Merck silica gel 60 F_{254}' or 'Merck aluminium oxide 60 F_{254} neutral' type UV-active silica or alumina sheets. Column chromatography was performed using a Teledyne Isco CombiFlash® Rf+ automated flash chromatographer with 'RediSep R_f GOLD' silica gel or basic alumina column at 25(±1) °C. The cartridge was filled with Zeochem® 'ZEOprep 60 25-40 µm' silica gel or EcoChrom™ 'MP Alumina B - Super I' basic alumina. Analytical RP-HPLC-UV/Vis-MS measurements were carried out using a Shimadzu LCMS-2020 instrument applying a Gemini C18 column (100 x 2.00 mm I.D.) in which the stationary phase is 5 µm silica with a pore size of 110 Å. The chromatograms were detected by a UV-Vis diode array (190-800 nm) and an ESI-MS detector. The following linear gradient elution profile was applied: 0 min 0 % B; 2.5 min 100% B; 3.25 100% B; 3.75 min 0% B; 5 min 0% B) with eluent A (0,1% HCOOH, 5% acetonitrile and 95% water) and B (0,1% HCOOH, 95% acetonitrile and 5% water) at a flow rate of 0.8-mL/min at 40 °C. Room temperature refers to 25(±1) °C.

NMR Spectroscopy. NMR experiments were carried out on a, 600, 500, 400 and 300 MHz Varian NMR System spectrometers equipped with inverse detection probes at 30 °C unless otherwise stated. Notation for the splitting patterns in the ^{1}H NMR spectra includes singlet (s), doublet (d), triplet (t), broad (br) and multiplet or overlapping peaks (m). Chemical shifts (δ values) are reported in ppm, coupling constants (J) are expressed in Hertz. ^{1}H and ^{13}C assignments were obtained using the combination of two-dimensional ^{1}H-^{1}H COSY, ^{1}H-^{1}H TOCSY, ^{1}H-^{1}H ROESY or ^{1}H-^{1}H NOESY, ^{1}H-^{13}C HSQC and ^{1}H-^{13}C HMBC measurements.

In the NMR spectra, ^{1}H assignments are marked in blue, while ^{13}C assignments are marked in red on the corresponding compound schemes. In cases where insufficient correlation data prevent the unambiguous assignment of atoms, an asterisk (*) indicates the interchangeable positions.

UV-Vis Absorption Spectroscopy. UV-Vis absorption spectroscopy was performed on a Jasco V-750 spectrophotometer. Data were collected from 200 to 800 nm using a 0.5 nm data interval and 400 nm/s scan speed. Hellma Analytics high-precision quartz cuvettes were used with optical path length of 1.0 cm. Spectroscopic grade solvents are used to prepare the solutions. Spectra were recorded in 3×10^{-5} M solutions at 25 (±1) °C. Baseline correction was used for every solvent.

HRMS Spectroscopy. High-resolution measurements were performed on a Sciex TripleTOF 5600+ high-resolution tandem mass spectrometer equipped with a DuoSpray ion source. ESI ionization was applied in the positive ion detection mode. Samples were dissolved in acetonitrile and flow injected into the acetonitrile/water 1:1 flow. The flow rate was 0.2 mL/min. The resolution of the mass spectrometer was 35000.

Cyclic voltammetry. CV curves were recorded with a WaveNowXV electrochemical workstation using a standard three-electrode setup (Three Electrode Cell Kit, Low Volume) from PINE Research Instrumentation

S2 Syntheses

5-Methyl-1,3-bis(dimethylamino)-2-azapentalene (S1)

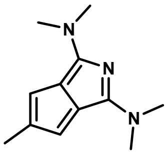

1,3-Bis(dimethylamino)-2-azapentalene-5-carboxaldehyde[1] (230 mg, 1.06 mmol) was dissolved in glacial acetic acid (12 mL), the solution was cooled to 10 °C then solid $NaBH_4$ (240 mg, 6.35 mmol) was added slowly. During addition, the mixture changed from orange to raspberry red as gas bubbles formed. After the addition, the reaction mixture was allowed to warm to room temperature. The reaction was monitored with LC-MS. After 30 min, the conversion of the starting material was complete. The reaction mixture was diluted with DCM (100 mL), transferred to beaker and the mixture was diluted with water (100 mL), then neutralized by adding solid Na_2CO_3 with intense stirring. When the gas formation stopped, the organic phase was separated with a separatory funnel, then the aqueous phase was extracted with DCM (1 x 100 mL). The combined organic phase was dried over $MgSO_4$, filtered and the solvent was evaporated to dryness to yield a dark purple solid. The crude product was diluted with DCM, evaporated onto Celite, then purified by column chromatography [basic alumina, DCM + 0.5% TEA → DCM/methanol (10%) + 0.5% TEA]. The product (**S1**) was obtained as a purple crystalline solid (206 mg, 96%).[1]

^{1}H NMR (500 MHz, $CDCl_3$) δ = 5.97 (s, 2H), 3.33 (s, 6H), 3.25 (s, 6H), 2.12 (s, 3H) ppm; ^{13}C{^{1}H} NMR (126 MHz, $CDCl_3$) δ = 170.4, 129.3, 121.5, 115.4, 39.8, 38.4, 14.9 ppm. HRMS (ESI$^+$): m/z: [M + H]$^+$, calculated for $[C_{12}H_{18}N_3]^+$: 204.1495, found: 204.1502.

5-(*tert*-Butyl)-1,3-bis(dimethylamino)-2-azapentalene (1)

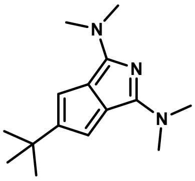

<u>*Step 1:*</u> To abs. THF (25 mL) in a two-necked flask (250 mL), under Ar atmosphere at 0 °C 6,6-dimethylfulvene (*Note that this compound is prone to decomposition, sensitive to water and oxygen, therefore, it must be stored at -20 °C under Ar atmosphere and opened only when used.*) (1.96 g, 1.88 mL, 18.5 mmol) was added dropwise in 4 h using a syringe pump. Subsequently, a solution of CH_3Li in diethyl ether (*Note that the best results were obtained when its bottle was freshly opened.*) (11.6 mL, 18.5 mmol, 1.6 M) was added dropwise with a syringe pump in 4 h.[2] The mixture was stirred for 16 h at 0 °C (using a cryostat), and the crude *tert*-butyl-cyclopentadienyl lithium salt was used immediately in *Step 3* without isolation or purification *and without allowing to warm up*.

Step 2: (Dichloromethylene)-dimethylammonium chloride (*Note that the best results were obtained when its bottle was freshly opened.*) (3.00 g, 18.5 mmol) was dissolved in abs. DCM (15 mL) in a vial (30 mL), then dimethylcyanamide (1.29 g, 1.44 mL, 18.5 mmol) was added to the solution under an inert atmosphere (Ar) at rt. The reaction mixture was stirred for 45 min until the colorless suspension turned into a yellowish solution, showing the formation of the desired cyanine derivative.[3] The crude product was used immediately (*Note that the product is strongly hygroscopic white solid material, therefore, it should be stored under Ar in solution if not used immediately.*) in *Step 3* without isolation or purification.

Step 3: The solution of *tert*-butyl-cyclopentadienyl lithium salt from *Step 1* at 0 °C under Ar atmosphere was diluted with abs. THF (120 ml), while keeping the temperature at 0 °C (using a cryostat). Then the cyanine derivative from *Step 2* was added dropwise over 2 h using a syringe pump, upon which the solution turned dark red. The reaction mixture was allowed to warm to rt and stirred at this temperature for 12 h, during which the formation of a red precipitate was observed. To the reaction mixture DCM (200 mL) and water (100 mL) was added, this mixture was transferred into a separatory funnel and 5% HCl solution (50 mL) was added. (*Note that the acid treatment of the reaction mixture must be carried out quickly, as the product decomposes under such conditions, but the step cannot be omitted.*) Subsequently, the two-phase system was transferred into a beaker and solid Na_2CO_3 was added portionwise while stirring until gas formation was no longer observed (pH > 7). The organic phase was then separated and dried over $MgSO_4$, filtered and the solvent was evaporated under reduced pressure. The crude product is a dark purplish red solid. The crude product was dissolved in DCM, Celite was added, and the slurry was evaporated to dryness, then purified by column chromatography [basic alumina, DCM + 0.5% TEA → DCM/methanol (5%) + 0.5% TEA]. The product was further purified by recrystallization from DCM : hexane 1:5 (200 mL). The resulting product is a purple solid (852 mg, 19%).

^1H NMR (500 MHz, $CDCl_3$) δ = 6.06 (s, 2H), 3.31 (s, 6H), 3.27 (s, 6H), 1.26 (s, 9H) ppm; $^{13}C\{H\}$ NMR (126 MHz, $CDCl_3$) δ = 170.6, 145.9, 121.0, 111.7, 39.8, 38.4, 32.3 ppm. HRMS (ESI^+): m/z: $[M + H]^+$, calculated for $[C_{15}H_{24}N_3]^+$: 246.1965, found: 246.1971.

Further attempts to optimize the synthesis of compound 1

- If the resulting solution of *Step 1* was allowed to warm to rt the isolated yield decreased.
- In case the reaction time was reduced to 6 or 10 h in *Step 1*, the isolated yield decreased significantly.
- In *Step 1,* faster addition of 6,6-dimethylfulvene and CH_3Li led to the increase of temperature and led to lower isolated yield. The optimal addition rate was 4 hours.
- The attempted isolation of the product of *Step 1* led to significant degradation.
- In *Step 2*, abs. THF cannot be used because (dichloromethylene)-dimethylammonium chloride is not soluble in it.
- In *Step 3*, changing the solvent to DCM or hexane significantly reduced the isolated yield.
- In *Step 3*, when the solution of *tert*-butyl-cyclopentadienyl lithium was cooled to -30 °C instead of 0 °C and (dichloromethylene)-dimethylammonium chloride was added, no significant amount of product was isolated.
- At the end of *Step 3*, the addition of 5% HCl solution is essential, otherwise the product will degrade during workup and purification.
- In *Step 3*, during purification, column chromatography before recrystallization cannot be omitted to obtain pure product.

5-(*tert*-Butyl)-1,3-bis(piperidinyl)-2-azapentalene (S2)

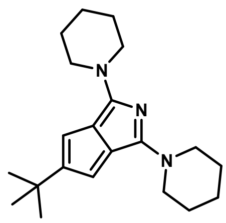

Method A: Azapentalene **1** (50.0 mg, 204 µmol) was dissolved in abs. toluene (5.0 mL) and piperidine (604 µL, 6.11 mmol) was added. The resulting mixture was stirred at 80 °C (in an aluminum heating block) for 4 days. The reaction was monitored with LC-MS. When the reaction was completed, the solvent was evaporated under reduced pressure. The crude product was a dark purple solid, which was purified by column chromatography [basic alumina, hexane → hexane/EtOAc (25%)]. The product (**S2**) was obtained as a purple solid (34.2 mg, 52%).

Method B: Azapentalene **1** (30.0 mg, 122 µmol) was dissolved in abs. toluene (2.0 mL) and piperidin (362 µL, 3.67 mmol) was added, and the mixture was stirred in an MW reactor at 200 °C for 6 hours in a sealed reaction vessel using the following method: from rt the reaction mixture was heated ("as fast as possible" option) to 200 °C, then held at this temperature for 3 hours, finally cooled back to 55 °C, while the power was controlled by IR-based temperature

controller. Afterwards, the conversion was checked using LC-MS, which was not complete, therefore, the mixture was stirred for another 3 hours at 200 °C using the same MW method. Workup and purification steps were identical to those in *Method A* (36.7 mg, 92%).

^{1}H NMR (300 MHz, $CDCl_3$) δ = 6.07 (s, 2H), 3.91 – 3.80 (m, 4H), 3.77 – 3.67 (m, 4H), 1.85 – 1.53 (m, 12H), 1.28 (s, 9H) ppm; ^{13}C{H} NMR (75 MHz, $CDCl_3$) δ = 169.2, 145.3, 120.6, 110.2, 49.0, 47.0, 32.4, 32.2, 26.1, 26.0, 24.3 ppm. HRMS (ESI$^+$): m/z: $[M + H]^+$, calculated for $[C_{21}H_{32}N_3]^+$: 326.2591, found: 326.2588.

5-(*tert*-Butyl)-1-(indolin-1-yl)-3-dimethylamino-2-azapentalene (S3)

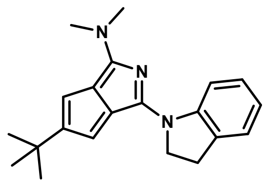

<u>*Method A:*</u> Azapentalene **1** (50.0 mg, 204 µmol) was dissolved in abs. toluene (5.0 mL) and indoline (687 µL, 6.11 mmol; filtered through a pad of silica [hexane → hexane/EtOAc (20%)] prior to reaction to remove potential impurities) was added. The resulting mixture was stirred at 80 °C (in an aluminum heating block) for 4 days. The reaction was monitored with LC-MS. When the reaction was completed, the solvent was evaporated under reduced pressure. The crude product was a dark liquid, which was purified by column chromatography [silica, hexane/EtOAc (5%) → hexane/EtOAc (20%)]. The product (**S3**) was obtained as a silver gray solid (41.8 mg, 64%).

<u>*Method B:*</u> Azapentalene **1** (50 mg, 204 µmol) was dissolved in abs. toluene (2.0 mL) and indoline (687 µL, 6.11 mmol; filtered through a silica column [hexane → hexane/EtOAc (20%)] before reaction to remove potential impurities) was added, and the mixture was stirred in an MW reactor at 150 °C for overall 9 hours in a sealed reaction vessel using the following method: from rt the reaction mixture was heated ("as fast as possible" option) to 150 °C, then held at this temperature for 3 hours, finally cooled back to 55 °C, while the power was controlled by IR-based temperature measurement. The conversion was then checked by LC-MS, which was not complete, so the mixture was stirred for two additional 3-hour periods using the same MW method, and the conversion was checked after each period. Workup and purification steps were identical to those in *Method A* (42.3 mg, 65%).

^{1}H NMR (500 MHz, CD_2Cl_2) δ = 8.43 (d, J = 8.1 Hz, 1H), 7.25 – 7.18 (m, 2H), 7.03 (t, J = 7.4 Hz, 1H), 6.08 (s, 1H), 6.00 (s, 1H), 4.22 (t, J = 8.3 Hz, 2H), 3.40 (s, 3H), 3.29 (t, J = 8.6 Hz, 2H), 3.26 (s, 3H), 1.23 (s, 9H) ppm; ^{13}C{H} NMR (126 MHz, CD_2Cl_2) δ = 171.0,

166.0, 146.8, 143.6, 133.8, 127.9, 125.2, 124.5, 123.6, 120.1, 119.0, 115.0, 113.6, 50.7, 40.2, 39.1, 32.3, 28.2 ppm. HRMS (ESI$^+$): m/z: $[M + H]^+$, calculated for $[C_{21}H_{26}N_3]^+$: 320.2121, found: 320.2135.

General procedure for the preparation of azaazulene derivatives (GP)

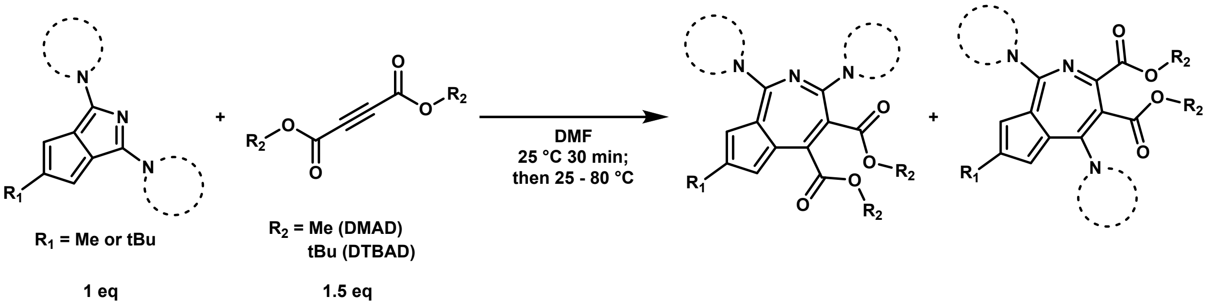

The azapentalene derivative (1 eq; in general 30 mg starting material was used, otherwise noted) was dissolved in DMF (40 mM), and then dimethyl or di-*tert*-butyl acetylenedicarboxylate (DMAD or DTBAD) (1.5 eq) was added with stirring at rt. Upon adding the acetylenedicarboxylate derivative the solution turned to reddish-yellow. After stirring for 30 min at 25°C, the reaction mixture was heated to a given temperature (25 – 110 °C) until the conversion was complete. The reaction was monitored with LC-MS. When the reaction was completed, the solvent was removed on a rotary evaporator at 60 °C. The crude product was purified by column chromatography [silica, hexane/EtOAc (20%) → hexane/EtOAc (50%)].

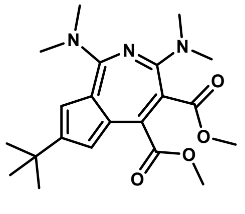

Azaazulene 2 The product was prepared according to **GP** from azapentalene **1** (30.0 mg, 122 µmol) and DMAD (26.1 mg, 22.5 µL, 183 µmol) in DMF (3.0 mL). The reaction was stirred at rt for 30 min and then at 50 °C for 3 h. After purification, the product is obtained as a yellow solid (38.7 mg, 82%).

^{1}H NMR (400 MHz, $CDCl_3$) δ = 6.72 (d, J = 2.1 Hz, 1H), 6.47 (d, J = 2.1 Hz, 1H), 3.97 (s, 3H), 3.78 (s, 3H), 3.31 (s, 6H), 3.07 (s, 6H), 1.29 (s, 9H) ppm; ^{13}C{H} NMR (101 MHz, $CDCl_3$) δ = 170.1, 166.1, 158.0, 156.6, 147.7, 146.7, 121.6, 118.4, 115.2, 114.8, 95.9, 52.6, 51.8, 40.84, 39.5, 32.3, 32.0 ppm. HRMS (ESI$^+$): m/z: $[M + H]^+$, calculated for $[C_{21}H_{30}N_3O_4]^+$: 388.2231, found: 388.2249.

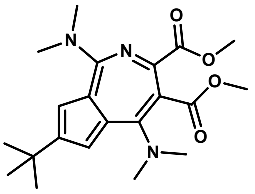

Azaazulene 6 The product was prepared according to **GP** from azapentalene **1** (30.0 mg, 122 µmol) and DMAD (26.1 mg, 22.5 µL, 183 µmol) in CH_3CN (3.0 mL). The reaction mixture was stirred at rt for 6.5 hours. The formation of both compound **6** (20.8 mg, 44%) and compound **2** (21.2 mg, 45%) was observed under these conditions. The products are yellow solids.

^{1}H NMR (500 MHz, $CDCl_3$) δ = 6.62 (d, J = 2.1 Hz, 1H), 6.53 (d, J = 2.1 Hz, 1H), 3.84 (s, 3H), 3.73 (s, 3H), 3.39 (s, 6H), 3.26 (s, 6H), 1.32 (s, 9H) ppm; ^{13}C{H} NMR (126 MHz, $CDCl_3$) δ = 168.9, 166.9, 163.8, 160.9, 150.7, 146.9, 124.1, 115.0, 113.6, 111.1, 103.3, 52.5, 51.9, 44.8, 41.0, 32.4, 32.2 ppm. HRMS (ESI$^+$): m/z: [M + H]$^+$, calculated for $[C_{21}H_{30}N_3O_4]^+$: 388.2231, found: 388.2246.

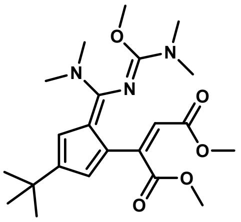

Fulvene F1 The product was prepared according to **GP** from azapentalene **1** (30.0 mg, 122 µmol) and DMAD (26.1 mg, 22.5 µL, 183 µmol) in MeOH. (3.0 mL). The reaction mixture was stirred at rt for 4.5 hours. After the rotary evaporation, the crude product was purified by column chromatography [silica, DCM → DCM/methanol (10%)]. Compound **F1** was obtained as a dark yellow solid (34.3 mg, 67%).

NMR measurements were carried out at 50 °C: ^{1}H NMR (400 MHz, $CDCl_3$) δ = 6.16 (d, J = 2.3 Hz, 1H), 5.98 (d, J = 2.3 Hz, 1H), 5.24 (s, 1H), 3.91 (s, 3H), 3.77 (s, 3H), 3.65 (s, 3H), 3.28 (brs, 6H), 2.74 (s, 6H), 1.17 (s, 9H) ppm; ^{13}C{H} NMR (101 MHz, $CDCl_3$) δ = 170.9, 167.8, 165.9, 162.7, 148.7, 141.0, 119.4, 118.9, 117.2, 109.8, 100.6, 56.3, 52.1, 50.9, 40.5 (brs), 38.3, 32.2, 31.7 ppm. HRMS (ESI$^+$): m/z: [M + H]$^+$, calculated for $[C_{22}H_{34}N_3O_5]^+$: 420.2493, found: 420.2491.

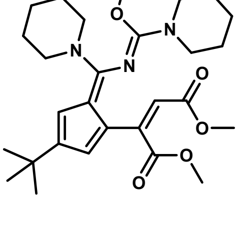

Fulvene F2 The product was prepared according to **GP** from azapentalene **S2** (25.9 mg, 79.6 µmol) and DMAD (17.0 mg, 14.7 µL, 119 µmol) in MeOH (3.0 mL). The reaction mixture was stirred at rt for 5 hours. After the rotary evaporation, the crude product was purified by column chromatography [silica, DCM → DCM/methanol (10%)]. Compound **F2** was obtained as a dark yellow solid (21.2 mg, 53%).

NMR measurements were carried out at 50 °C: ^{1}H NMR (400 MHz, $CDCl_3$) δ = 6.15 (d, J = 2.3 Hz, 1H), 5.96 (d, J = 2.4 Hz, 1H), 5.30 (s, 1H), 3.90 (s, 3H), 3.81 (s, 3H), 3.79 (brs, 4H), 3.66 (s, 3H), 3.28 – 3.20 (m, 4H), 1.71 – 1.62 (m, 6H), 1.46 – 1.41 (m, 2H), 1.35 – 1.28 (m, 4H), 1.17 (s, 9H) ppm; ^{13}C {H} NMR (101 MHz, $CDCl_3$) δ = 171.0, 167.9, 164.4, 161.0, 148.8, 140.7, 119.7, 119.3, 116.5, 110.0, 100.0, 56.1, 52.0, 50.9, 49.6 (brs), 47.2, 32.3, 31.7, 26.2, 25.3, 24.7, 23.9 ppm. HRMS (ESI^{+}): m/z: [M + H]$^{+}$, calculated for $[C_{28}H_{42}N_3O_5]^{+}$: 500.3119, found: 500.3116.

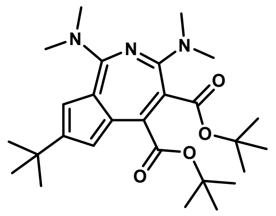

7

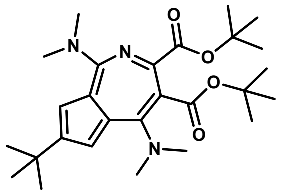

8

Azaazulenes 7 and 8 These products were prepared according to **GP** from azapentalene **1** (30.0 mg, 122 µmol) and DTBAD (41.5 mg, 183 µmol) in DMF (3.0 mL). The reaction was stirred for 30 min at rt and then at 80 °C for 16 hours. After purification, the products were obtained as yellow solids (**7**: 12.9 mg, 22%; **8**: 9.6 mg, 17%).

Product 7:

^{1}H NMR (500 MHz, $CDCl_3$) δ = 6.66 (d, J = 2.1 Hz, 1H), 6.64 (d, J = 2.1 Hz, 1H), 3.31 (s, 6H), 3.06 (s, 6H), 1.62 (s, 9H), 1.51 (s, 9H), 1.29 (s, 9H) ppm; ^{13}C{H} NMR (126 MHz, $CDCl_3$) δ = 168.2, 165.2, 157.9, 156.5, 147.6, 146.6, 121.7, 116.3, 114.8, 114.7, 99.0, 81.8, 80.3, 40.9, 39.3, 32.3, 32.1, 28.6, 28.3 ppm. HRMS (ESI^{+}): m/z: [M + H]$^{+}$, calculated for $[C_{27}H_{42}N_3O_4]^{+}$: 472.3170, found: 472.3189; [M – ^{t}Bu + H]$^{+}$, calculated for $[C_{23}H_{34}N_3O_4]^{+}$: 416.2544, found: 416.2562; [M – 2 ^{t}Bu + H]$^{+}$, calculated for $[C_{19}H_{26}N_3O_4]^{+}$: 360.1918, found: 360.1931.

*Product **8***:

^{1}H NMR (500 MHz, $CDCl_3$) δ = 6.63 (d, J = 2.2 Hz, 1H), 6.51 (d, J = 2.1 Hz, 1H), 3.40 (s, 6H), 3.24 (s, 6H), 1.53 (s, 9H), 1.50 (s, 9H), 1.35 (s, 9H) ppm; ^{13}C{H} NMR (126 MHz, $CDCl_3$) δ = 167.3, 166.0, 164.1, 160.7, 150.9, 146.3, 124.2, 114.2, 114.1, 110.7, 105.7, 81.4, 80.9, 44.7, 41.0, 32.4, 32.3, 28.5, 28.2 ppm. HRMS (ESI^{+}): m/z: [M + H]$^{+}$, calculated for $[C_{27}H_{42}N_3O_4]^{+}$: 472.3170, found: 472.3167; [M – ^{t}Bu + H]$^{+}$, calculated for $[C_{23}H_{34}N_3O_4]^{+}$: 416.2544, found: 416.2539; [M – 2 ^{t}Bu + H]$^{+}$, calculated for $[C_{19}H_{26}N_3O_4]^{+}$: 360.1918, found: 360.1933.

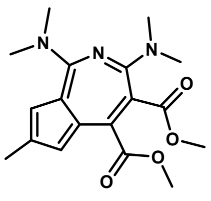

Azaazulene 9 The product was prepared according to **GP** from azapentalene **S1** (15.5 mg, 76.2 µmol) and DMAD (16.3 mg, 14.1 µL, 114 µmol) in DMF (2.0 mL). The reaction was stirred for 30 min at rt and then at 50 °C for 3 h. After purification, the product was obtained as a yellow solid (9.8 mg, 37%).

^{1}H NMR (400 MHz, $CDCl_3$) δ = 6.57 (dd, J = 1.9, 0.6 Hz, 1H), 6.42 (dq, J = 1.7, 0.8 Hz, 1H), 3.96 (s, 3H), 3.78 (s, 3H), 3.30 (s, 6H), 3.08 (brs, 6H), 2.27 (brs, 3H) ppm; $^{13}C\{H\}$ NMR (101 MHz, $CDCl_3$) δ = 169.9, 165.9, 157.9, 156.3, 146.5, 131.8, 121.9, 121.2, 118.2, 115.4, 96.3, 52.6, 51.8, 40.7, 39.4 (brs), 15.3 ppm. HRMS (ESI^+): m/z: $[M + H]^+$, calculated for $[C_{18}H_{24}N_3O_4]^+$: 346.1761, found: 346.1760.

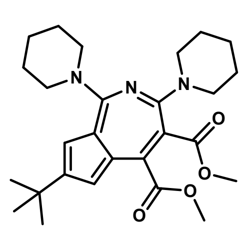

Azaazulene 10 The product was prepared according to **GP** from azapentalene **S2** (30.0 mg, 92.2 µmol) and DMAD (19.6 mg, 17.1 µL, 138 µmol) in DMF (3.0 mL). The reaction was stirred at rt for 30 min and then at 50 °C for 2 h. After purification, the product was obtained as a yellow solid (35.7 mg, 83%).

NMR measurements were carried out at 50 °C: ^{1}H NMR (500 MHz, $CDCl_3$) δ = 6.64 (d, J = 2.1 Hz, 1H), 6.46 (d, J = 2.1 Hz, 1H), 3.96 (s, 3H), 3.84 – 3.79 (m, 4H), 3.76 (s, 3H), 3.56 (brs, 4H), 1.76 – 1.65 (m, 6H), 1.65 – 1.59 (m, 2H), 1.56 (brs, 4H), 1.29 (s, 9H) ppm; $^{13}C\{H\}$ NMR (126 MHz, $CDCl_3$) δ = 170.1, 166.3, 157.2, 156.3, 147.8, 146.8, 122.0, 117.0, 115.4, 115.1, 96.3, 52.5, 51.6, 49.6, 48.1, 32.3, 32.0, 26.6, 25.8, 25.0, 24.6 ppm. HRMS (ESI^+): m/z: $[M + H]^+$, calculated for $[C_{27}H_{38}N_3O_4]^+$: 468.2857, found: 468.2861.

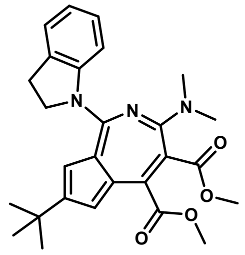

11

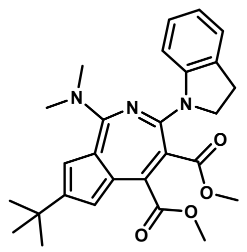

12

Azaazulenes 11 and 12 These products were prepared according to **GP** from azapentalene **S3** (45.4 mg, 142 µmol) and DMAD (30.3 mg, 26.1 µL, 213 µmol). The reaction was carried out in DMF (4.5 mL) with K_2CO_3 (27.5 mg, 199 µmol) additive. The reaction was stirred at rt for 30 min and then at 60 °C for 2 days. After purification, the products were obtained as yellow solids (**11**: 19.3 mg, 29%; **12**: 23.4 mg, 36%).

*Product **11***:

^{1}H NMR (400 MHz, $CDCl_3$) δ = 7.19 (dt, J = 6.9, 1.3 Hz, 1H), 6.96 (td, J = 7.6, 1.4 Hz, 1H), 6.92 – 6.86 (m, 2H), 6.80 (d, J = 2.0 Hz, 1H), 6.64 (d, J = 2.0 Hz, 1H), 4.37 (t, J = 8.0 Hz, 2H), 4.02 (s, 3H), 3.79 (s, 3H), 3.15 (s, 6H), 3.13 (t, J = 8.2 Hz, 2H), 1.27 (s, 9H) ppm; ^{13}C{H} NMR (101 MHz, $CDCl_3$) δ = 169.9, 166.1, 159.0, 151.2, 149.5, 145.9, 144.6, 133.4, 126.2, 124.9, 122.6, 122.1, 119.7, 116.4, 116.2, 115.4, 96.1, 53.9, 52.8, 52.0, 39.7, 32.5, 31.9, 28.3 ppm. HRMS (ESI$^+$): m/z: [M + H]$^+$, calculated for $[C_{27}H_{32}N_3O_4]^+$: 462.2387, found: 462.2367.

*Product **12***:

^{1}H NMR (400 MHz, $CDCl_3$) δ = 7.30 – 7.24 (m, 1H), 7.18 – 7.13 (m, 1H), 7.09 (t, J = 7.7 Hz, 1H), 6.93 – 6.86 (m, 1H), 6.80 (d, J = 2.0 Hz, 1H), 6.61 (d, J = 2.0 Hz, 1H), 4.26 (brs, 2H), 4.04 (s, 3H), 3.40 (s, 6H), 3.30 (s, 3H), 3.09 (t, J = 7.5 Hz, 2H), 1.31 (s, 9H) ppm; ^{13}C{H} NMR (101 MHz, $CDCl_3$) δ = 170.1, 166.5, 156.8, 151.4, 149.8, 147.1, 143.6, 133.5, 127.6, 125.2, 123.2, 122.9, 118.9, 116.0, 115.3, 113.6, 97.6, 52.8, 52.6, 51.6, 41.3, 32.5, 31.9, 27.8 ppm. HRMS (ESI$^+$): m/z: [M + H]$^+$, calculated for $[C_{27}H_{32}N_3O_4]^+$: 462.2387, found: 462.2368.

S3 Scope and limitations and attempted post-synthetic modifications

Substituted azapentalenes and different acetylene reagents that did not yield azaazulene products are shown on Figure S1. In these cases, neither higher temperature (up to 80°C) nor the addition of K_2CO_3 (1.4 eq) led to product formation.

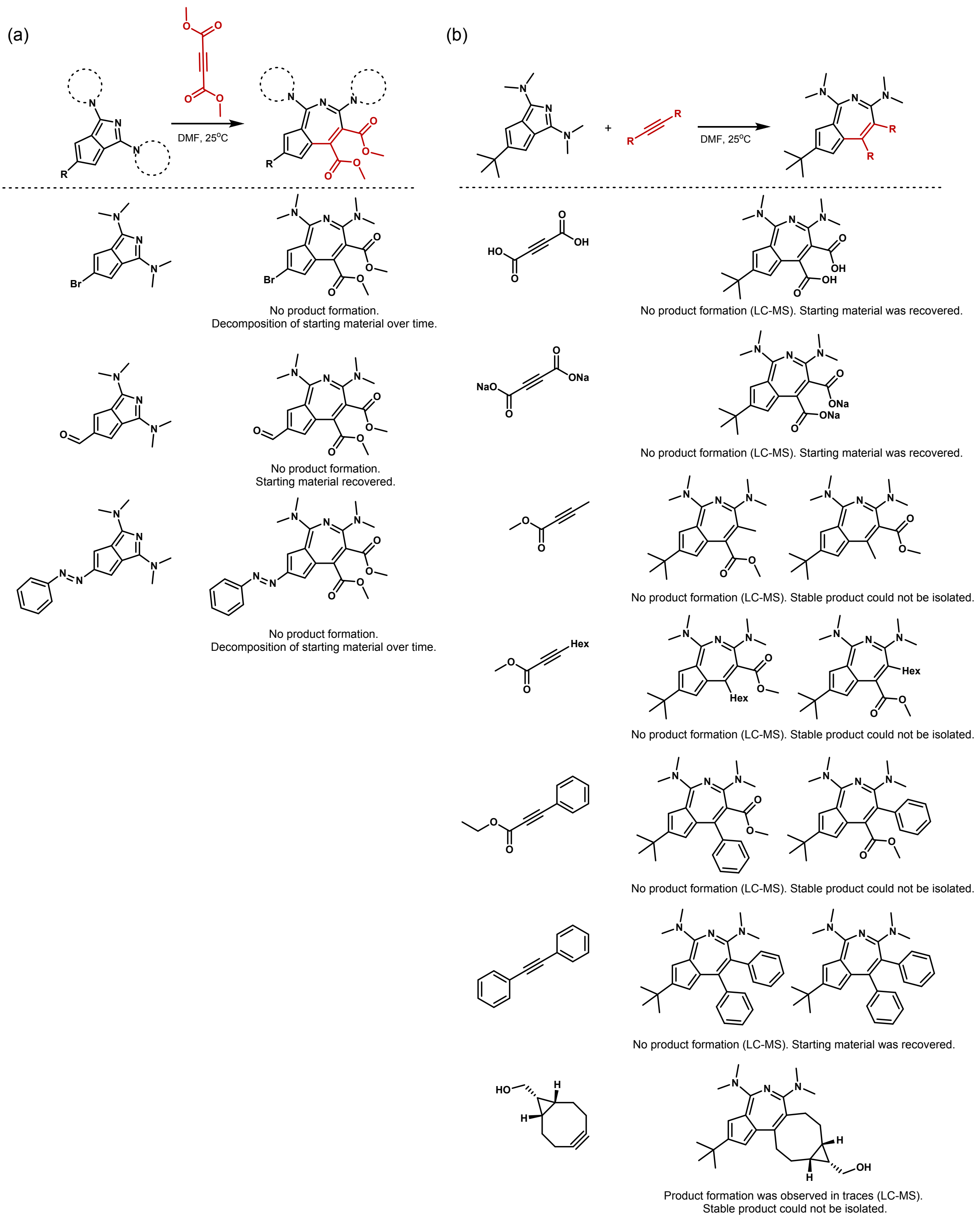

Figure S1. (a) The reactions of different azapentalene derivatives with DMAD. (b) The reactions of azapentalene **1** with different acetylene reagents.

Previously, azapentalene **S3** was reported to react with DMAD at position C5 (Figure S2).

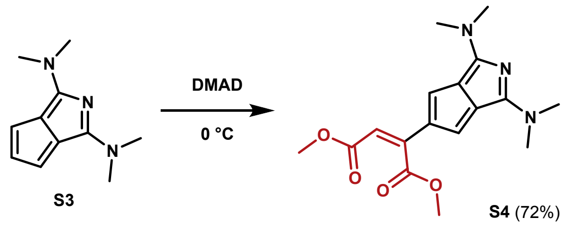

Figure S2. The reaction of **S3** with DMAD, as reported by Hafner and coworkers previously.

We tested the reactivity of **S3** in the presence of excess DMAD to probe whether **S4** undergoes ring expansion (Figure S3). In the presence of 10 equiv. DMAD we found the rapid formation of **S4** (based on LC-MS analysis), which reacted further and after 2h at rt a mixture of products was detected. At this point (rt, 2h) compounds with *m/z* values of 332 (10%), 474 (10%), 616 (40%) and 758 (40%) could be detected (based on this data their structures could not be identified unambiguously; possible isomers are suggested on Figure S3). As an attempt to push the reaction towards the more substituted products (*m/z* 758) we added an addition 10 equiv. of DMAD a heated the mixture to 60°C. After 24 h under these conditions *m/z* 332 was absent and the composition slightly changed (m/z 474 – 10%, 616 – 45%, 758 – 45%). After 36 h at 60°C *m/z* 474 almost disappeared (2%) and 616 (49%) and 758 (49%) somewhat increased. Attempts to isolate any of the products by column chromatography failed due to decomposition.

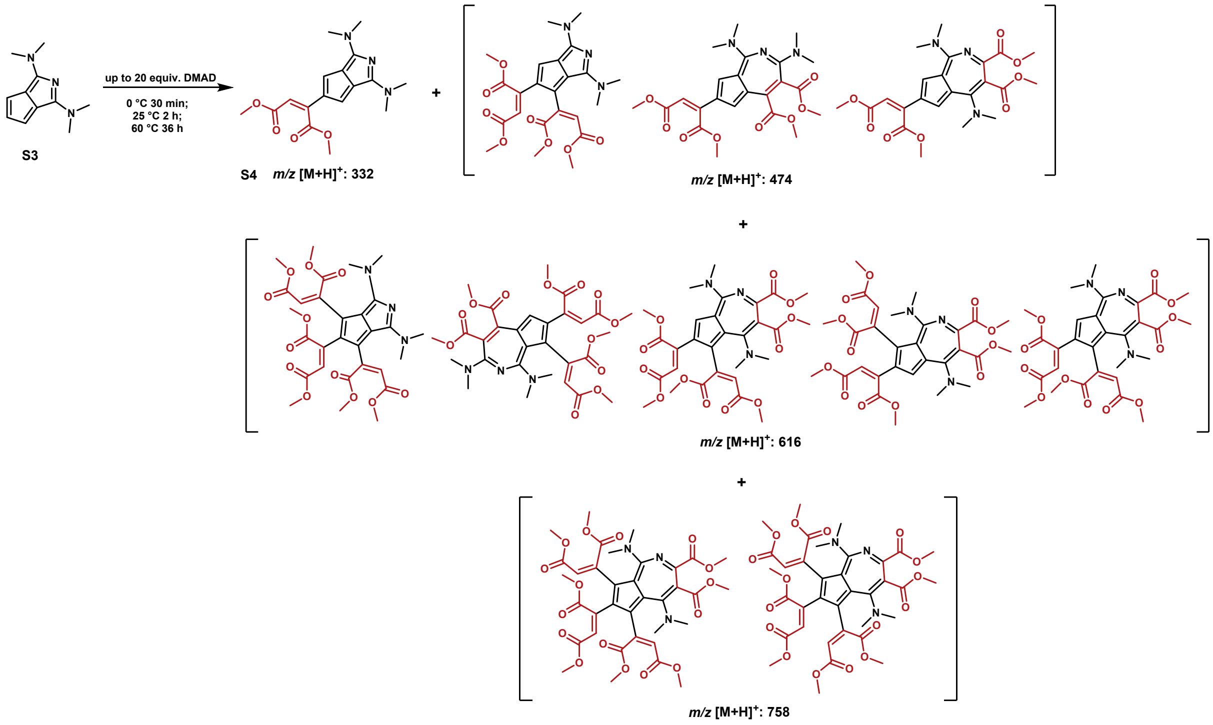

Figure S3. The reaction of S3 with excess DMAD.

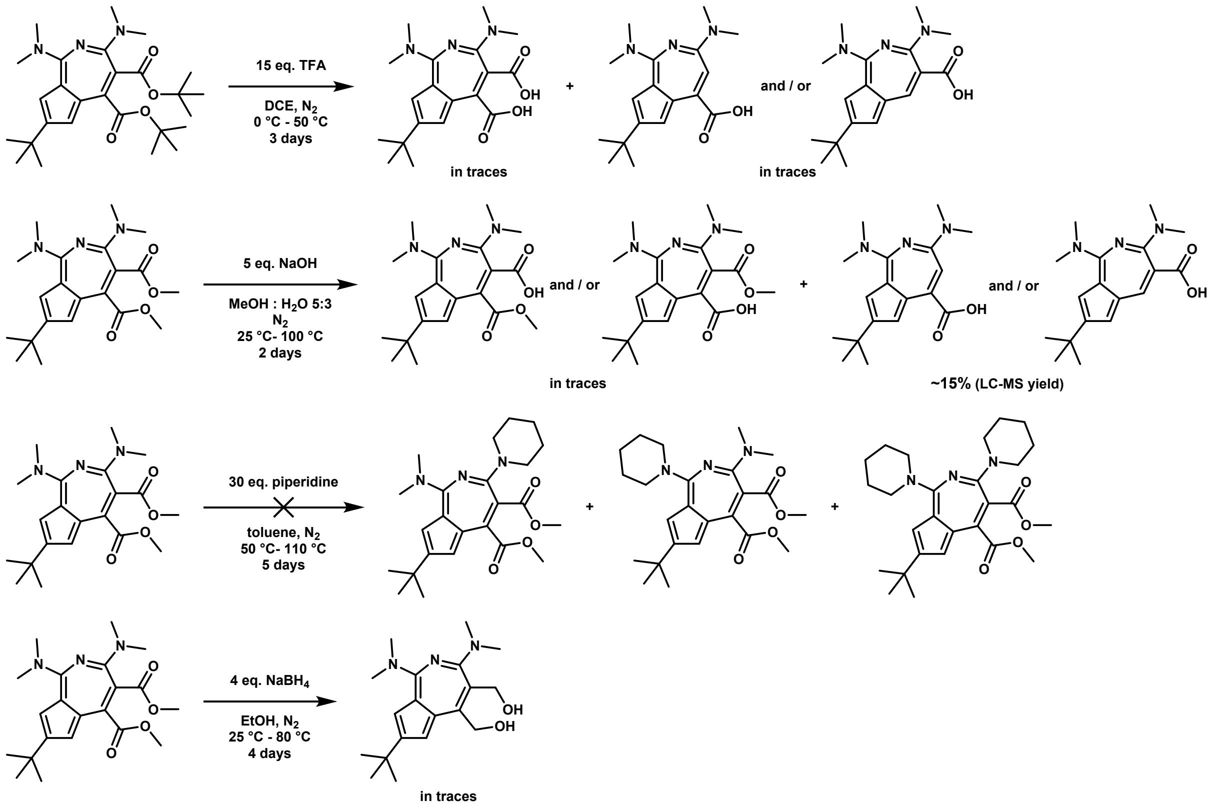

Figure S4. Attempted post-synthetic modifications of azaazulenes.

S4 Effect of bases

In case of the indoline-substituted azaazulenes **11** and **12**, the addition of K_2CO_3 base to the reaction mixture was found beneficial, although considerably slowed down product formation. Without the presence of the base the formation of these compounds could be tracked by LC-MS, however, their isolation was problematic in reasonable yields. It is likely that under the reaction conditions partial hydrolysis of DMAD occurs and acidic species lower the yield. Based on this finding we tested the effect of different bases on the reaction of azapentalene **1** and DMAD. We found that solid carbonate bases (K_2CO_3, Cs_2CO_3) slowed down the reaction considerable, but otherwise had no detrimental effect was observed. Other bases either decreased the yield or completely prevented the reaction.

Table S1. The effect of bases on the transformation of azapentalene **1** and DMAD under different conditions.

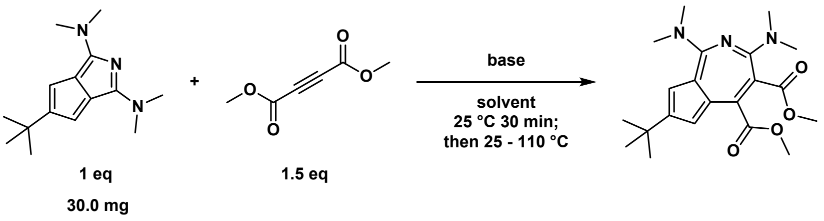

Base	Equiv.	Solvent	Temperature (°C)	Reaction time (h)	Isolated yield (%)
K_2CO_3	1.4	DMF	0 → 25	48	89
K_2CO_3	1.4	toluene	25 → 80	32	33
Cs_2CO_3	1.4	DMF	0 → 25	5.5	44
Cs_2CO_3	1.4	toluene	25 → 80	32	46
KOH	1.4	DMF	0 → 25	16	19
DBU	1.4	DMF	0 → 25	1.5	0[a]
DIPA	1.4	toluene	25 → 110	22	0[a]
pyrrolidine	1.4	toluene	25 → 110	22	0[a]
NaOMe	1.4	methanol	0 → 80	24	0[a]
TEA	1.4	DMF	25 → 50	5.5	74-78[b]
TEA	1.4	toluene	25 → 50	6.5	38-49[b]
TEA	176	(TEA)	25 → 50	22	15
TEA	0.2	toluene	25 → 50	6.5	30

[a] Loss of starting material was observed.
[b] Dependent on the scale (30-50 mg of **1**).

S5 Electrochemistry

Cyclic voltammetry (CV) experiments were performed in a standard three-electrode setup from PINE Research Instrumentation (Figure S5). Glassy carbon wires were used as the working electrodes (WE, 3 mm disk diameter), platinum as the counter electrodes (CE) and an Ag/AgCl wire as the pseudo-reference electrodes (RE). The recorded currents were normalized to the geometric surface area (7.1 mm^2) of the glassy carbon working electrode. We used IUPAC convention to plot CV.

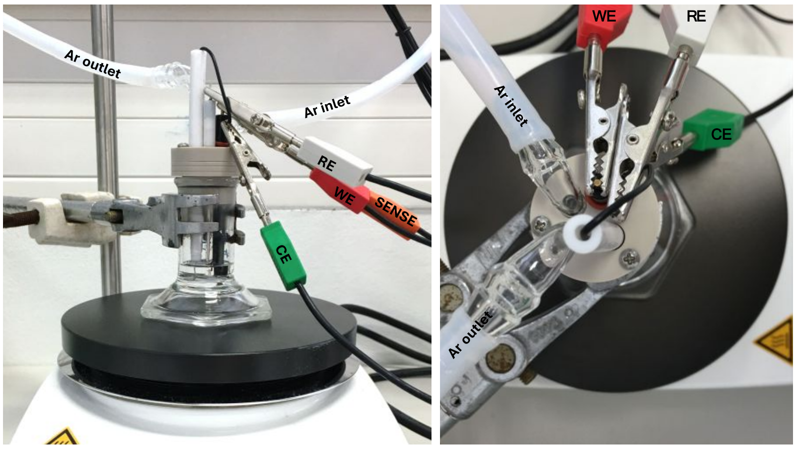

Figure S5. The electrochemical cell used for the measurements.

Polishing materials and methods

Before starting the measurements, the working electrode was first polished with a 5 µm alumina solution on 600-grit Silicon Carbide Paper, then switched to a 0.3 µm alumina suspension and Nylon Polishing Cloth, and finally, a 0.05 µm alumina suspension was applied with a MicroCloth Polishing Cloth. Between two consecutive measurements and after the measurements were completed, only the 0.05 um alumina suspension polishing was performed on the MicroCloth Polishing Cloth. All polishing materials were purchased from PINE Research Instrumentation.

General considerations for sample preparation

A solution (5 mL in HPLC grade THF) containing 0.1 M Bu_4NPF_6 (Fluorochem) as electrolyte was purged with Ar (saturated with THF vapor throughout the measurements) for 10 min then the measurement setup was tested (capacitive charging current was measured at 500 mV/s

sweep rate between -1400 mV and +1400 mV). We recorded all the measurements at rt from 0.00 V, and the direction of the initial scan was oxidative. The electroactive species was added (0.001 M) followed by 10 min purging with Ar and the voltammograms were measured. Measurements were also repeated after the addition of ferrocene (0.001 M, Acros Organics) as an internal standard. The headspace above the solution was gently purged with Ar (saturated with THF vapor) during each data acquisition. Cyclic voltammograms were measured at 25 mV/s, 100 mV/s and 250 mV/s sweep rates in the absence and presence of ferrocene. Ferrocene was used as an internal standard to interpret the measured data.

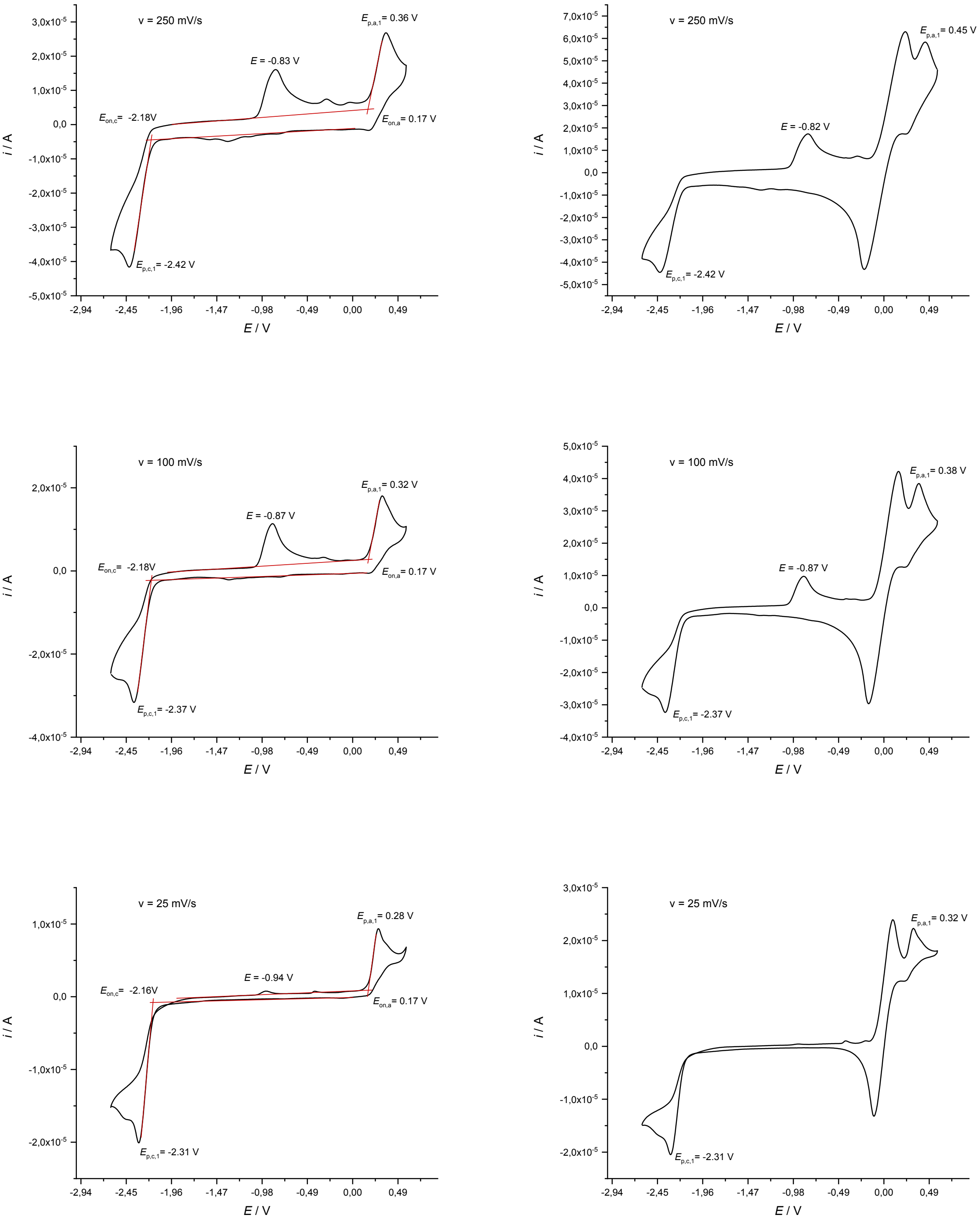

Figure S6. Cyclic voltammograms of compound **2** at different sweep rates with ferrocene (right column) and without ferrocene (left column).

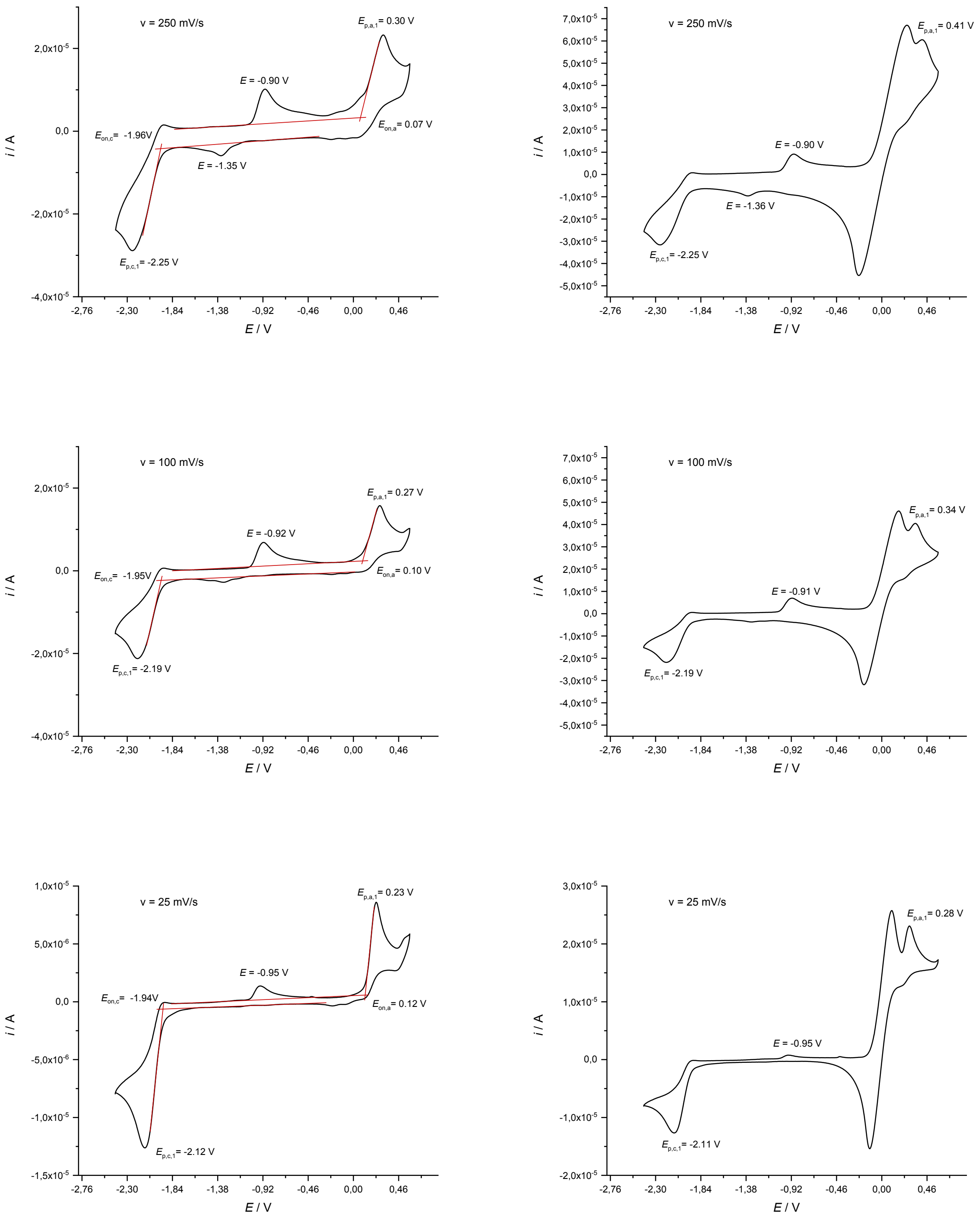

Figure S7. Cyclic voltammograms of compound **6** at different sweep rates with ferrocene (right column) and without ferrocene (left column).

S6 Computational details

All DFT calculations were performed using the ORCA 6.0 software.[4] The geometries were optimized with the modified version of the ωB97M-V functional with DFT-D4 correction ("wB97M-D4rev" keyword)[5] together with the def2-SVP basis set[6] and the corresponding def2/J auxiliary basis.[7] The solvent effect of DMF was included via the implicit Solvation Model Based on Density (SMD).[8] Frequency calculations were carried out to confirm the nature of the optimized stationary points (local energy minimum or transition state) and to obtain Gibbs energy correction based on the harmonic oscillator, rigid rotor, ideal gas approximations at 25 °C. To improve the electronic energies, single point calculations on the optimized geometries were performed using the larger def2-TZVPP basis set.[6] In several cases where molecular flexibility was expected to have large influence on the structures, globally optimal geometries were searched using the GOAT conformational analysis module of ORCA. These calculations involved GFN2-xTB energies[9] and ALPB solvation for DMF.[10]

For the excited state studies, the SCS-PBE-QIDH functional[11] and the def2-TZVP(-f) basis set[6] was chosen together with the def2/J[7] and def2-TZVP/C[12] auxiliary basis functions. The double hybrid functional includes doubles correction that improves the description of charge transfer states which are important for molecules with inverted singlet-triplet gaps.[13] The TDA approximation was used to calculate excitations as it reduces the triplet instability exhibited by full TD-DFT that usually leads to too low triplet state energies.[14] Vertical absorption energies for the first 60 singlet and triplet states were calculated together with oscillator strengths for the singlets. Solvation was also included via the linear response formalism based on the SMD solvent model for acetonitrile. The change in solvent from DMF meant that the azaazulene structures taken from the mechanistic studies were reoptimized at the ωB97M-V/def2-SVP level in acetonitrile and then the TDA-DFT calculations were performed on them. The hole-electron analysis was done with the Multiwfn software.[15]

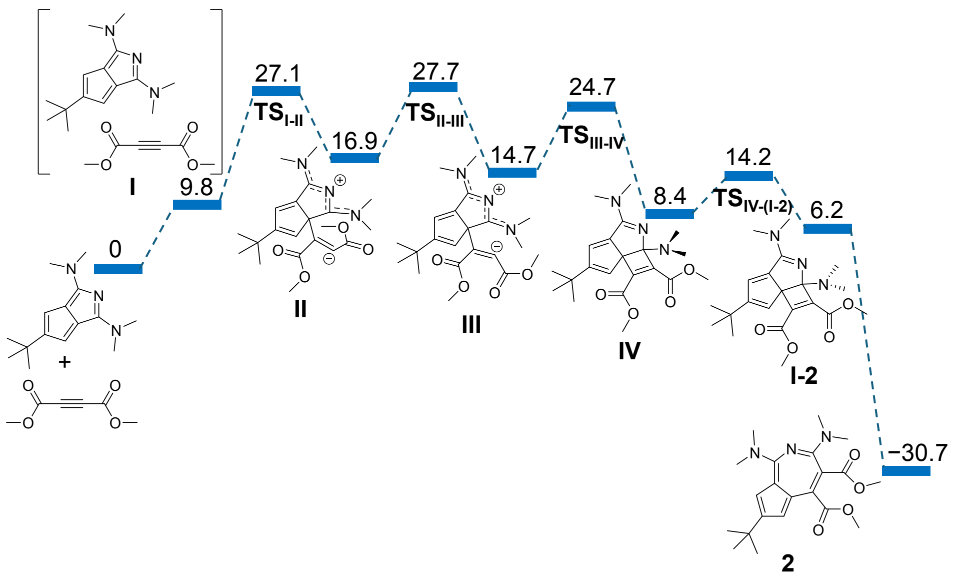

Figure S8. Free energy profile of the reaction between azapentalene **1** and DMAD leading to **2** (values are given in kcal/mol).

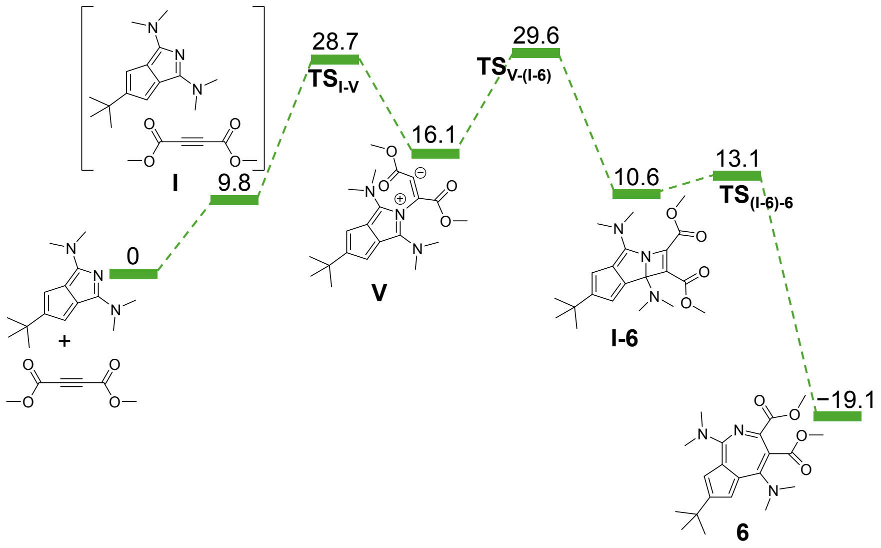

Figure S9. Free energy profile of the reaction between azapentalene **1** and DMAD leading to **6** (values are given in kcal/mol).

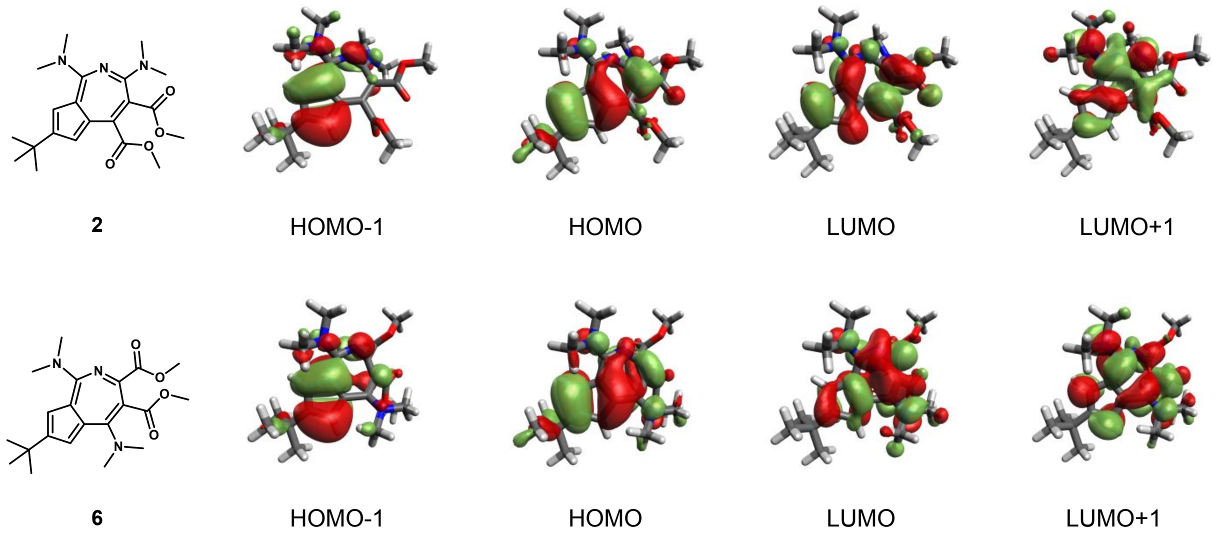

Figure S10. Molecular orbital depictions of compound **2** and **6**.

Table S2. Computational characterization of the excitation of **2**. (S_r – hole-electron overlap; D index – hole-electron distance)

Excitation	ΔE (eV)	λ (nm)	f	ΔΔE (kcal/mol)	S_r (a.u.)	D index (Å)	Hole (MO %)	Electron (MO %)
S0 → T1	2.571749	482	0	0.0	0.66112	1.176	103 (9%), 104 (85%)	105 (94%)
S0 → T2	3.012353	412	0	10.2	0.60745	1.624	103 (76%), 104 (14%)	105 (82%) 106 (6%)
S0 → S1	3.096861	400	0.3557	12.1	0.71829	1.286	103 (13%), 104 (81%)	105 (77%) 106 (18%)
S0 → T3	3.263108	380	0	15.9	0.60431	1.481	103 (10%), 104 (78%)	106 (87%)
S0 → S3	3.59374	345	0.1690	23.6	0.71534	1.65	103 (46%), 104 (50%)	105 (47%) 106 (49%)
S0 → S2	3.60706	344	0.0375	23.9	0.72862	1.602	103 (52%), 104 (45%)	105 (54%) 106 (43%)
S0 → T4	3.633738	341	0	24.5	0.56409	0.422	102 (37%), 103 (45%)	106 (87%)
S0 → S4	3.925777	316	0.8541	31.2	0.58695	1.926	103 (75%), 104 (15%)	105 (12%) 106 (80%)

Table S3. Computational characterization of the excitation of **6**. (S_r – hole-electron overlap; D index – hole-electron distance).

Excitation	ΔE (eV)	λ (nm)	f	ΔΔE (kcal/mol)	S_r (a.u.)	D index (Å)	Hole (MO %)	Electron (MO %)
$S_0 \rightarrow T_1$	2.762658	449	0	0.0	0.61882	1.678	102 (6%) 104 (87%)	105 (75%) 106 (18%)
$S_0 \rightarrow S_1$	2.927895	424	0.0530	3.8	0.6433	1.436	103 (27%) 104 (65%)	105 (83%) 106 (12%)
$S_0 \rightarrow T_3$	3.015051	411	0	5.8	0.60551	1.213	102 (18%) 103 (68%)	105 (67%) 106 (22%)
$S_0 \rightarrow T_2$	3.065833	404	0	7.0	0.67488	0.964	102 (18%) 103 (20%) 104 (49%)	105 (24%) 106 (64%)
$S_0 \rightarrow S_2$	3.341639	371	0.0634	13.4	0.59455	2.061	103 (75%) 104 (22%)	105 (86%) 106 (11%)
$S_0 \rightarrow T_4$	3.540976	350	0	17.9	0.58595	1.828	103 (66%) 104 (22%)	105 (20%) 106 (63%) 107 (5%)
$S_0 \rightarrow T_5$	3.626521	342	0	19.9	0.611	1.545	102 (39%) 103 (28%) 104 (18%)	105 (64%) 106 (17%) 107 (6%)
$S_0 \rightarrow S_3$	3.784811	328	0.4440	23.6	0.65111	1.585	103 (18%) 104 (76%)	105 (7%) 106 (87%)
$S_0 \rightarrow S_4$	3.840226	323	0.3995	24.9	0.5945	2.07	103 (63%) 104 (25%)	105 (21%) 106 (72%)

Table S4. Computational characterization of the excitation of **11**. (S_r – hole-electron overlap; D index – hole-electron distance).

Excitation	ΔE (eV)	λ (nm)	f	ΔΔE (kcal/mol)	S_r (a.u.)	D index (Å)	Hole (MO %)	Electron (MO %)
$S_0 \rightarrow T_1$	2.537678	489	0	0.0	0.73745	0.614	122 (16%) 123 (78%)	124 (85%) 125 (10%)
$S_0 \rightarrow T_2$	2.92485	424	0	8.9	0.77268	1.142	121 (13%) 122 (58%) 123 (25%)	124 (70%) 125 (19%)
$S_0 \rightarrow S_1$	2.981449	416	0.2395	10.2	0.7928	0.993	121 (7%) 122 (24%) 123 (66%)	124 (51%) 125 (45%)
$S_0 \rightarrow T_3$	3.00482	413	0	10.8	0.71836	1.056	122 (18%) 123 (69%)	124 (23%) 125 (64%)
$S_0 \rightarrow T_5$	3.276226	378	0	17.0	0.61063	1.092	119 (5%) 121 (32%) 122 (47%) 123 (10%)	124 (6%) 125 (87%)
$S_0 \rightarrow S_2$	3.317961	374	0.1104	18.0	0.66362	1.521	122 (8%) 123 (87%)	124 (46%) 125 (50%)
$S_0 \rightarrow S_3$	3.472087	357	0.2759	21.5	0.70853	1.394	121 (10%) 122 (76%) 123 (10%)	124 (75%) 125 (20%)
$S_0 \rightarrow S_4$	3.481876	356	0.1802	21.8	0.66734	1.691	121 (10%) 122 (70%) 123 (17%)	124 (19%) 125 (77%)
$S_0 \rightarrow T_4$	3.509268	353	0	22.4	0.85905	0.244	120 (17%) 121 (46%) 122 (16%) 123 (9%)	124 (23%) 125 (30%) 126 (16%) 127 (22%)

Table S5. Computational characterization of the excitation of **12**. (S_r – hole-electron overlap; D index – hole-electron distance).

Excitation	ΔE (eV)	λ (nm)	f	ΔΔE (kcal/mol)	S_r (a.u.)	D index (Å)	Hole (MO %)	Electron (MO %)
$S_0 \rightarrow T_1$	2.520719	492	0	0.0	0.72599	0.583	122 (18%) 123 (79%)	124 (95%)
$S_0 \rightarrow T_2$	2.920947	425	0	9.2	0.76479	1.096	121 (10%) 122 (62%) 123 (23%)	124 (77%) 125 (11%)
$S_0 \rightarrow S_1$	2.979335	416	0.3279	10.6	0.77886	0.799	122 (19%) 123 (76%)	124 (71%) 125 (24%)
$S_0 \rightarrow T_3$	3.144411	394	0	14.4	0.76235	0.645	120 (7%) 121 (15%) 122 (14%) 123 (56%)	124 (10%) 125 (67%) 126 (7%) 127 (10%)
$S_0 \rightarrow S_2$	3.315452	374	0.0727	18.3	0.66446	1.362	122 (6%) 123 (89%)	124 (12%) 125 (83%)
$S_0 \rightarrow T_4$	3.425690	362	0	20.9	0.79153	0.46	120 (12%) 121 (36%) 122 (12%) 123 (30%)	125 (62%) 126 (12%) 127 (17%)
$S_0 \rightarrow S_3$	3.540358	350	0.1471	23.5	0.65383	1.593	121 (8%) 122 (84%) 123 (6%)	124 (88%) 125 (8%)
$S_0 \rightarrow S_4$	3.647643	340	1.0581	26.0	0.68372	0.657	121 (10%), 122 (68%), 123 (17%)	124 (16%), 125 (76%)
$S_0 \rightarrow T_5$	3.662019	339	0	26.3	0.69621	0.9	120 (13%), 121 (9%), 122 (61%)	125 (60%), 126 (16%), 127 (12%)

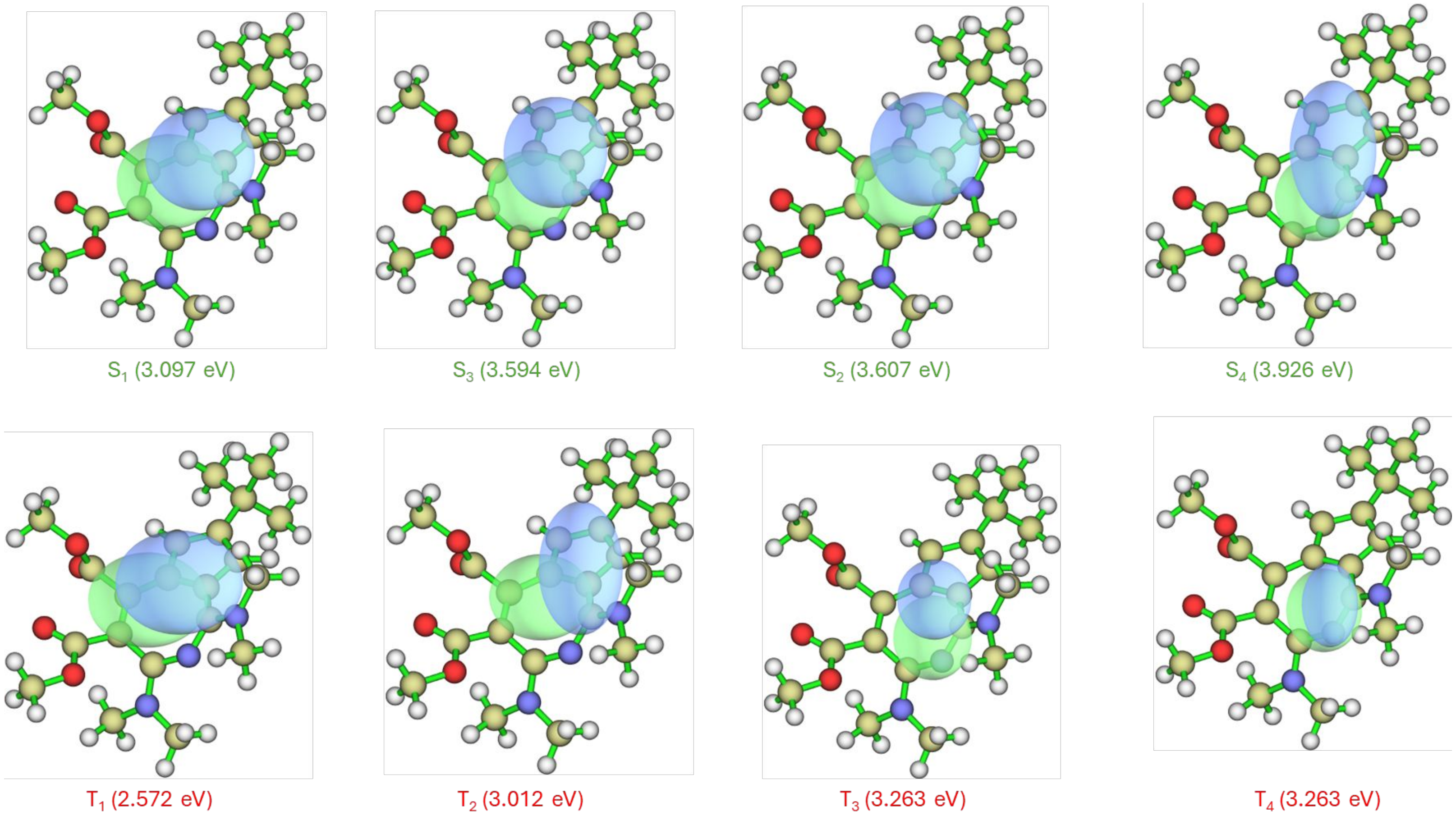

Figure S11. Isosurfaces calculated for the C_{ele} (green) and C_{hole} (blue) functions of the first 4 singlet and triplet excitations of **2**. An isovalue of 0.002 electrons/bohr^3 was used for the surfaces.

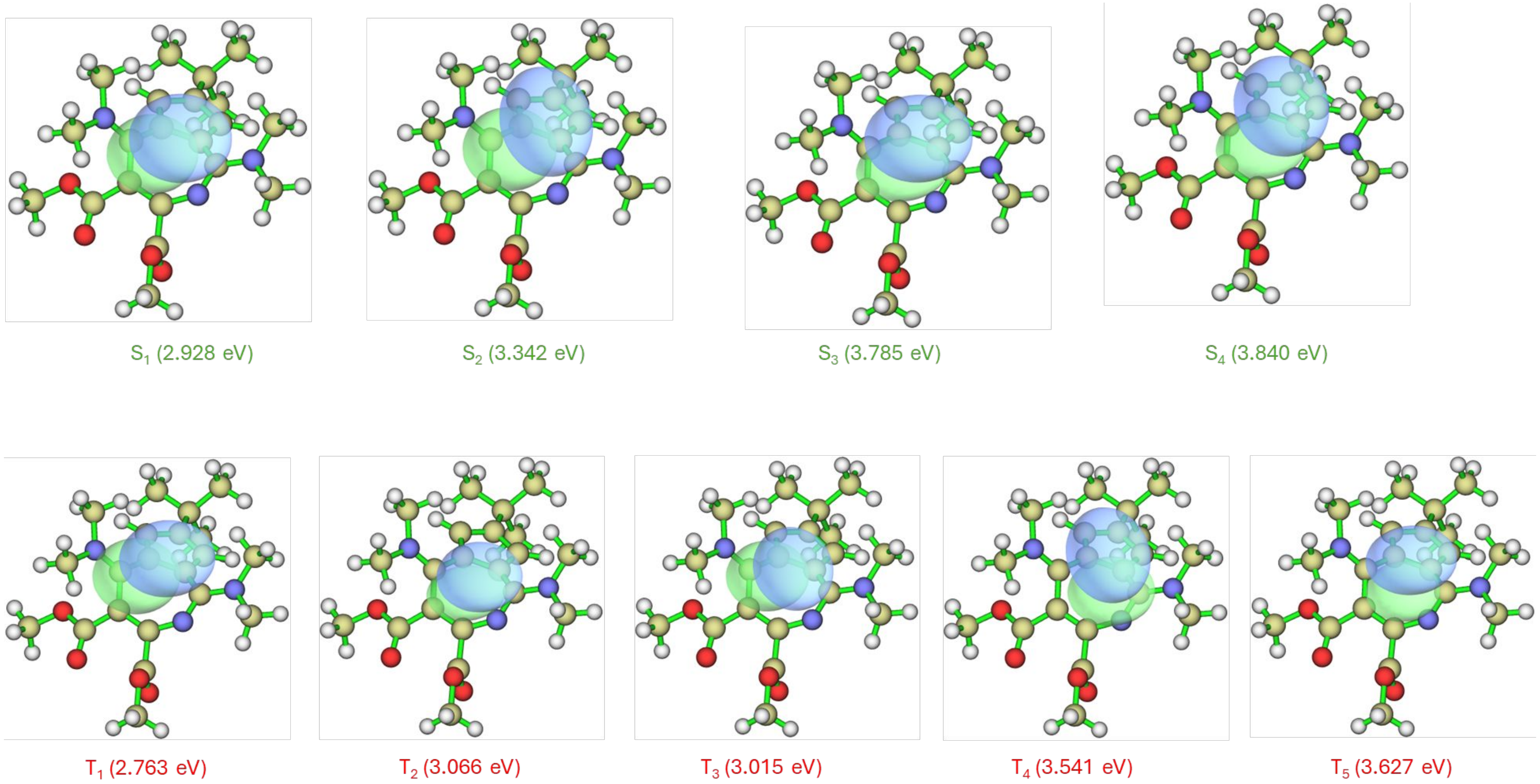

Figure S12. Isosurfaces calculated for the C_{ele} (green) and C_{hole} (blue) functions of the first 4 singlet and triplet excitations of **6**. An isovalue of 0.002 electrons/bohr^3 was used for the surfaces.

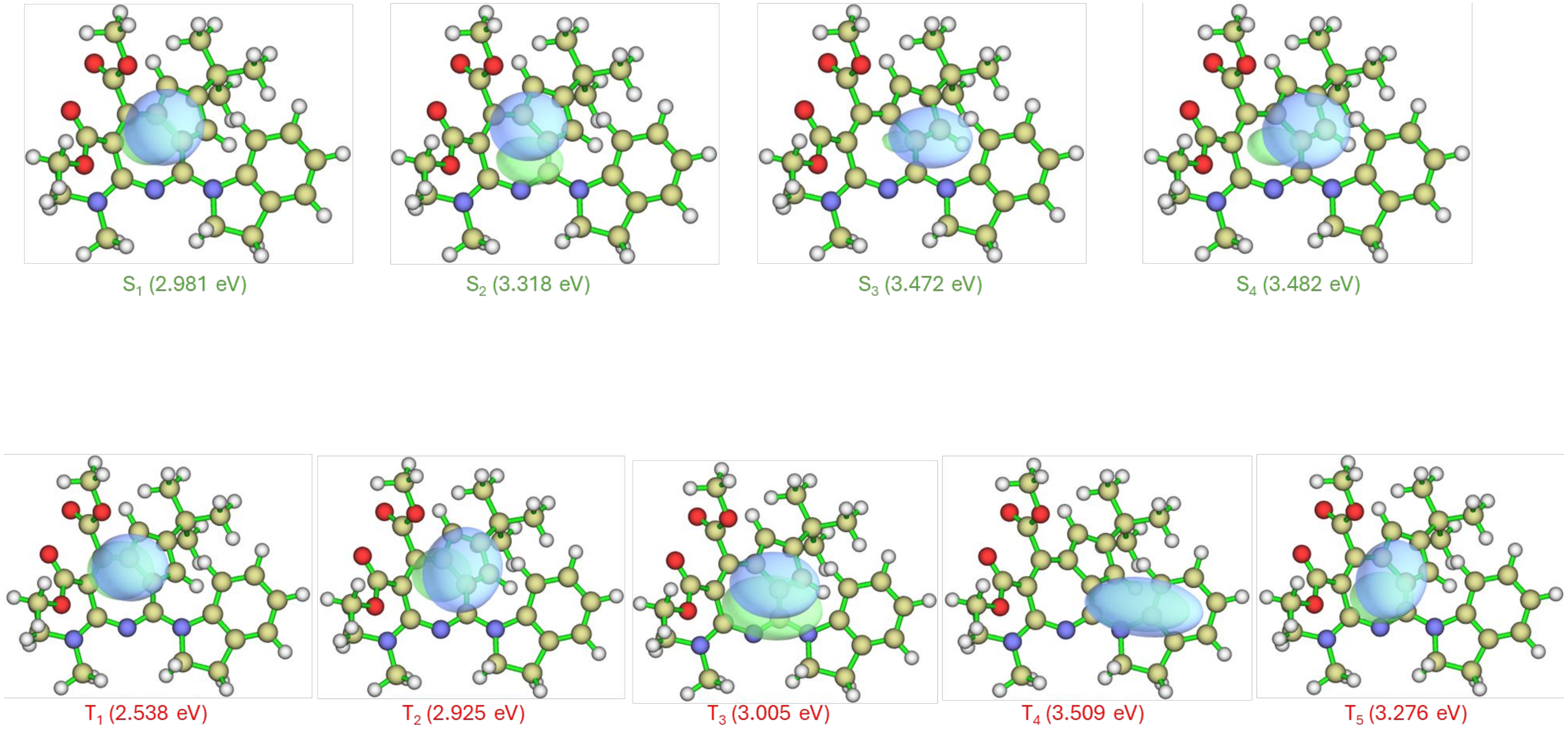

Figure S13. Isosurfaces calculated for the C_{ele} (green) and C_{hole} (blue) functions of the first 4 singlet and triplet excitations of **11**. An isovalue of 0.002 electrons/bohr^3 was used for the surfaces.

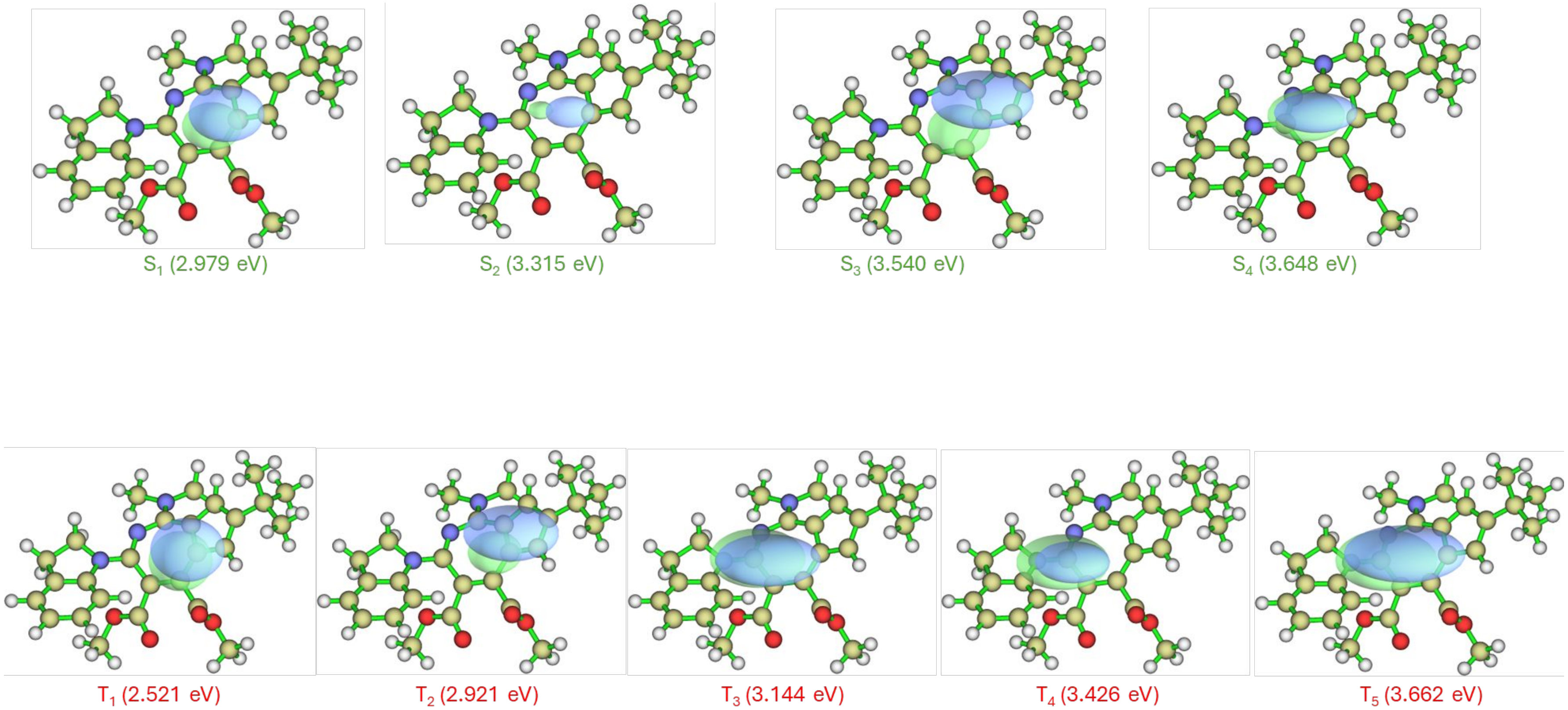

Figure S14. Isosurfaces calculated for the C_{ele} (green) and C_{hole} (blue) functions of the first 4 singlet and triplet excitations of **12**. An isovalue of 0.002 electrons/bohr^3 was used for the surfaces.

S7 NMR spectra

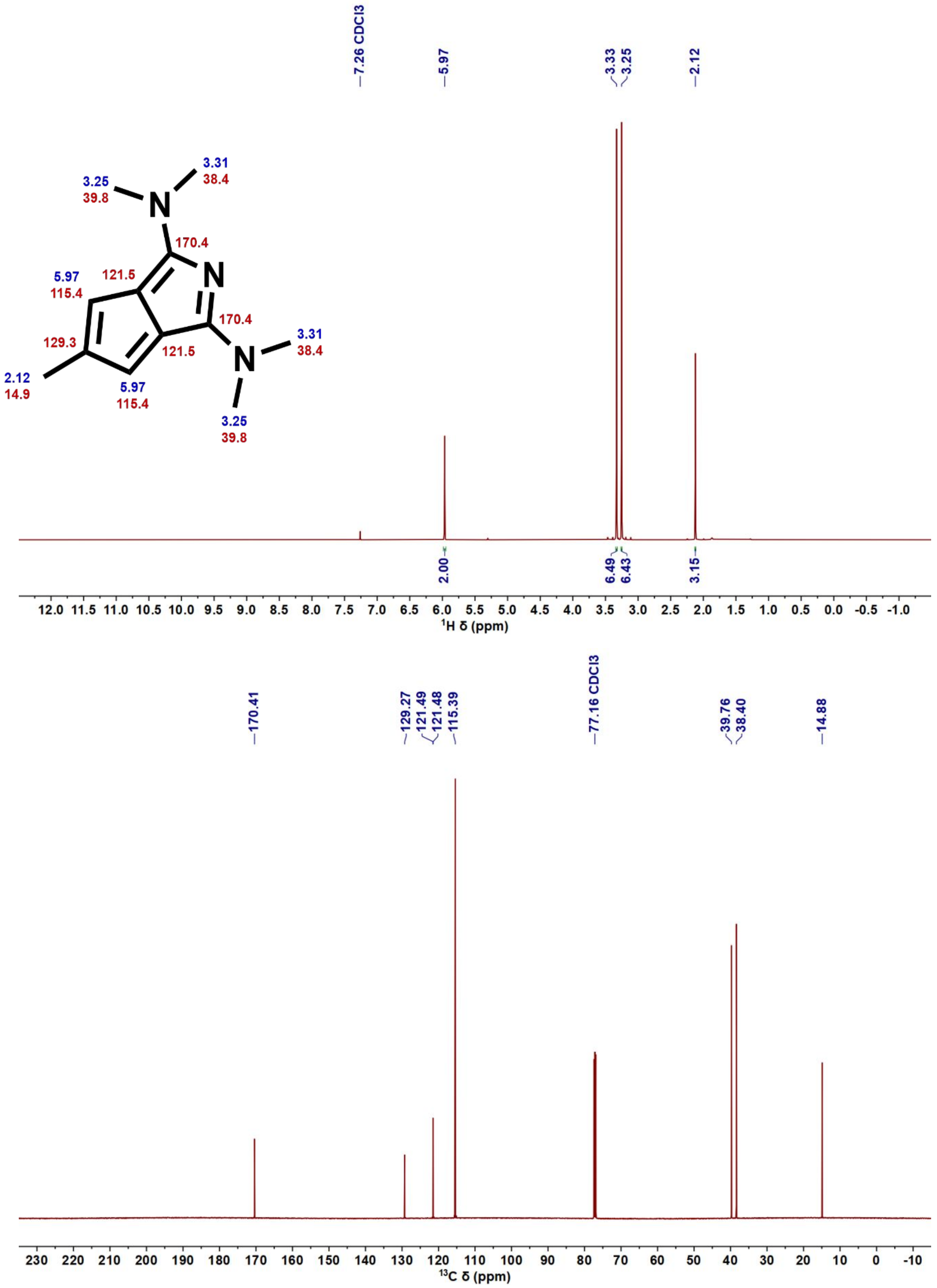

Figure S15. ^{1}H (500 MHz, top) and $^{13}C\{^{1}H\}$ (126 MHz, bottom) NMR spectra and assignments of **S1** in $CDCl_3$.

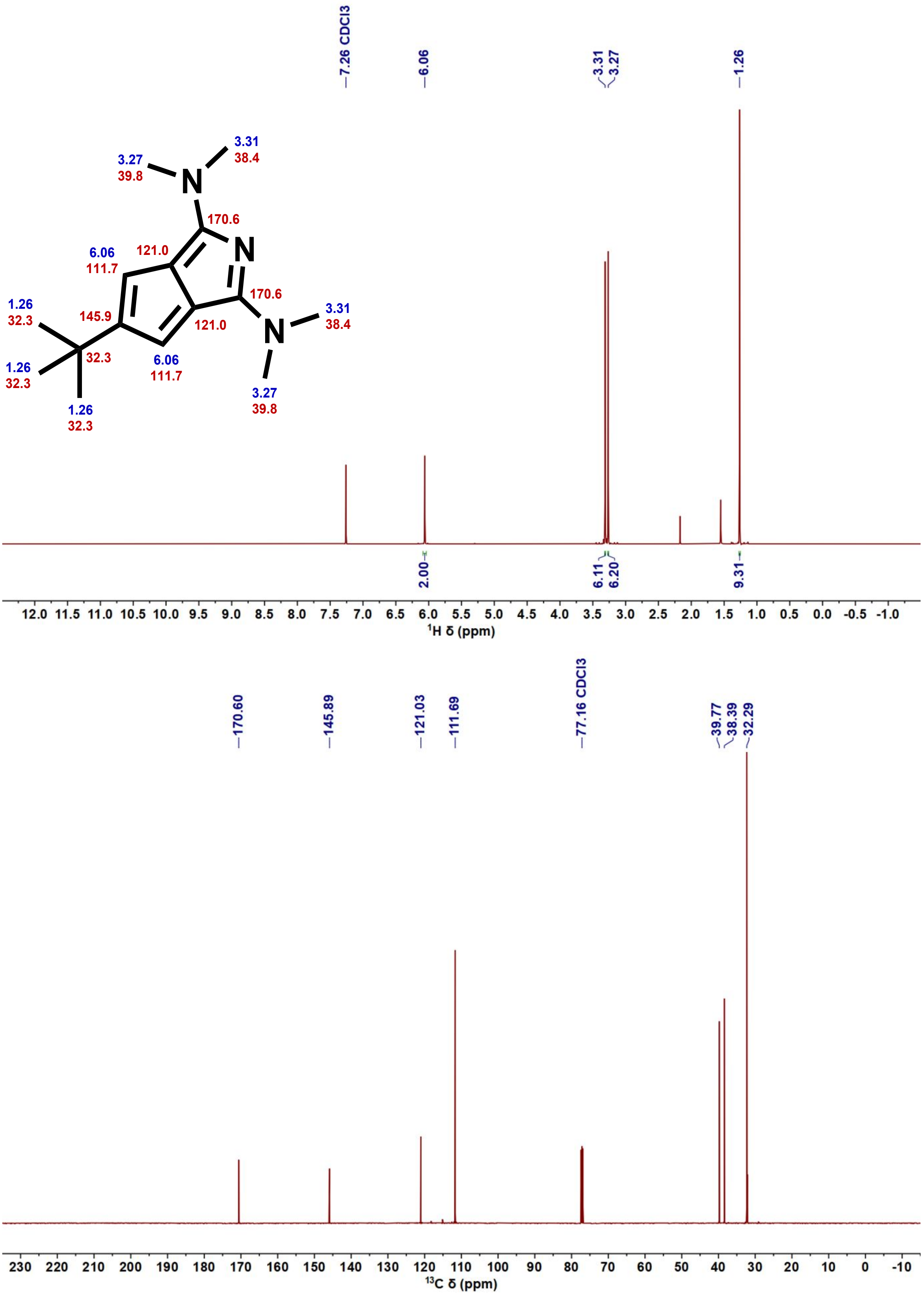

Figure S16. ^{1}H (500 MHz, top) and $^{13}C\{^{1}H\}$ (126 MHz, bottom) NMR spectra and assignments of **1** in $CDCl_3$.

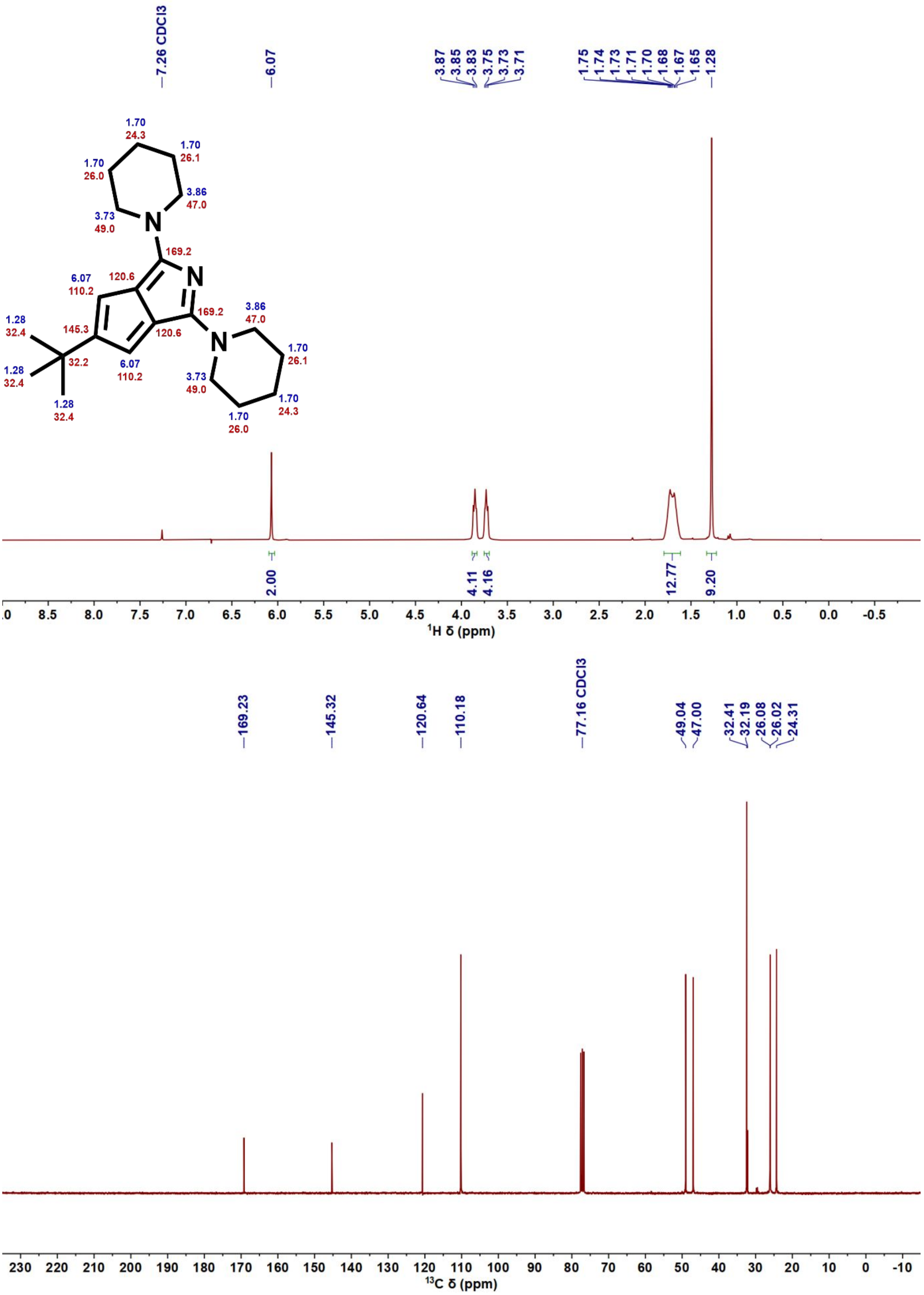

Figure S17. ^{1}H (300 MHz, top) and $^{13}C\{^{1}H\}$ (75 MHz, bottom) NMR spectra and assignments of **S2** in $CDCl_3$.

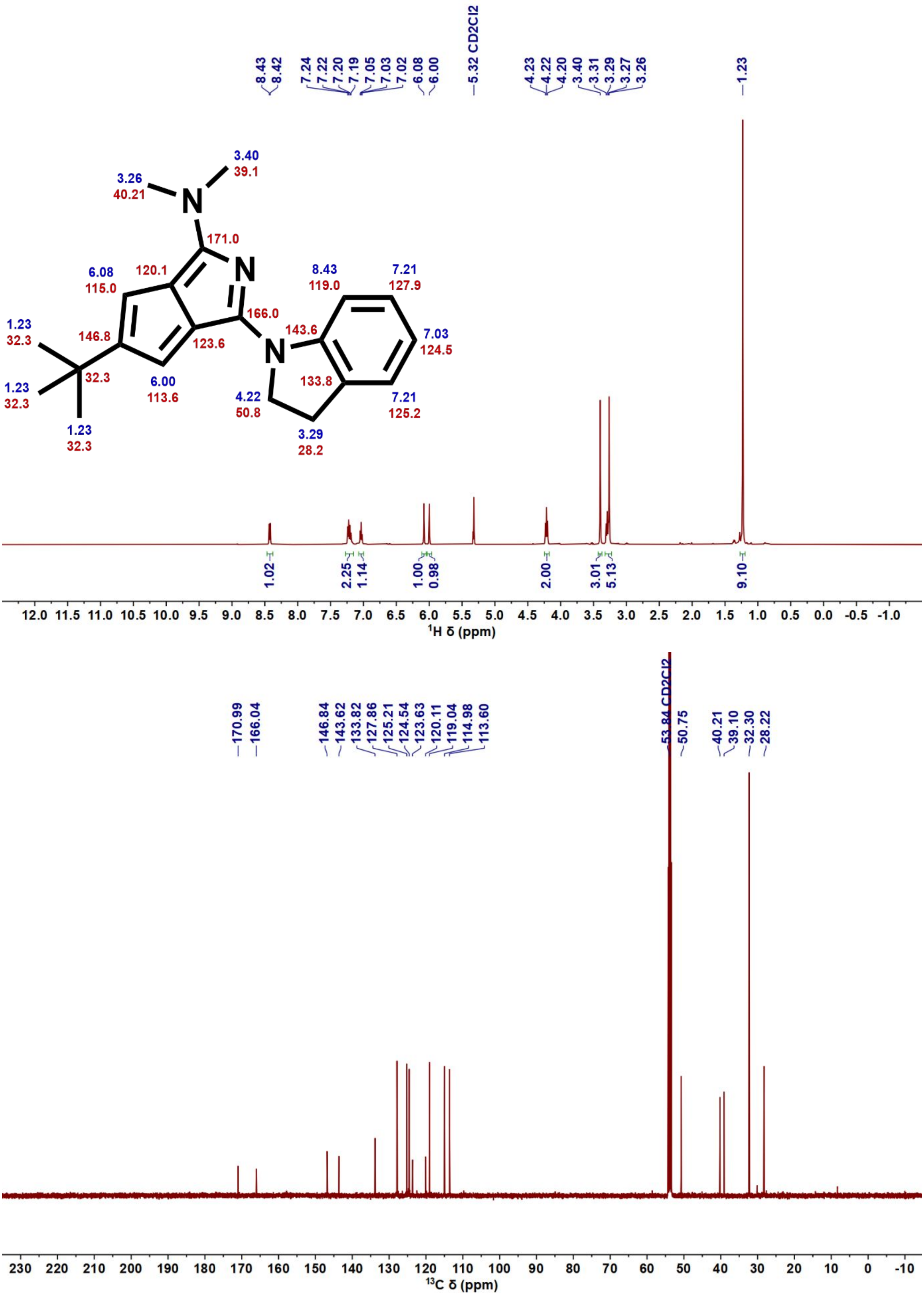

Figure S18. ^{1}H (500 MHz, top) and $^{13}C\{^{1}H\}$ (126 MHz, bottom) NMR spectra and assignments of **S3** in CD_2Cl_2.

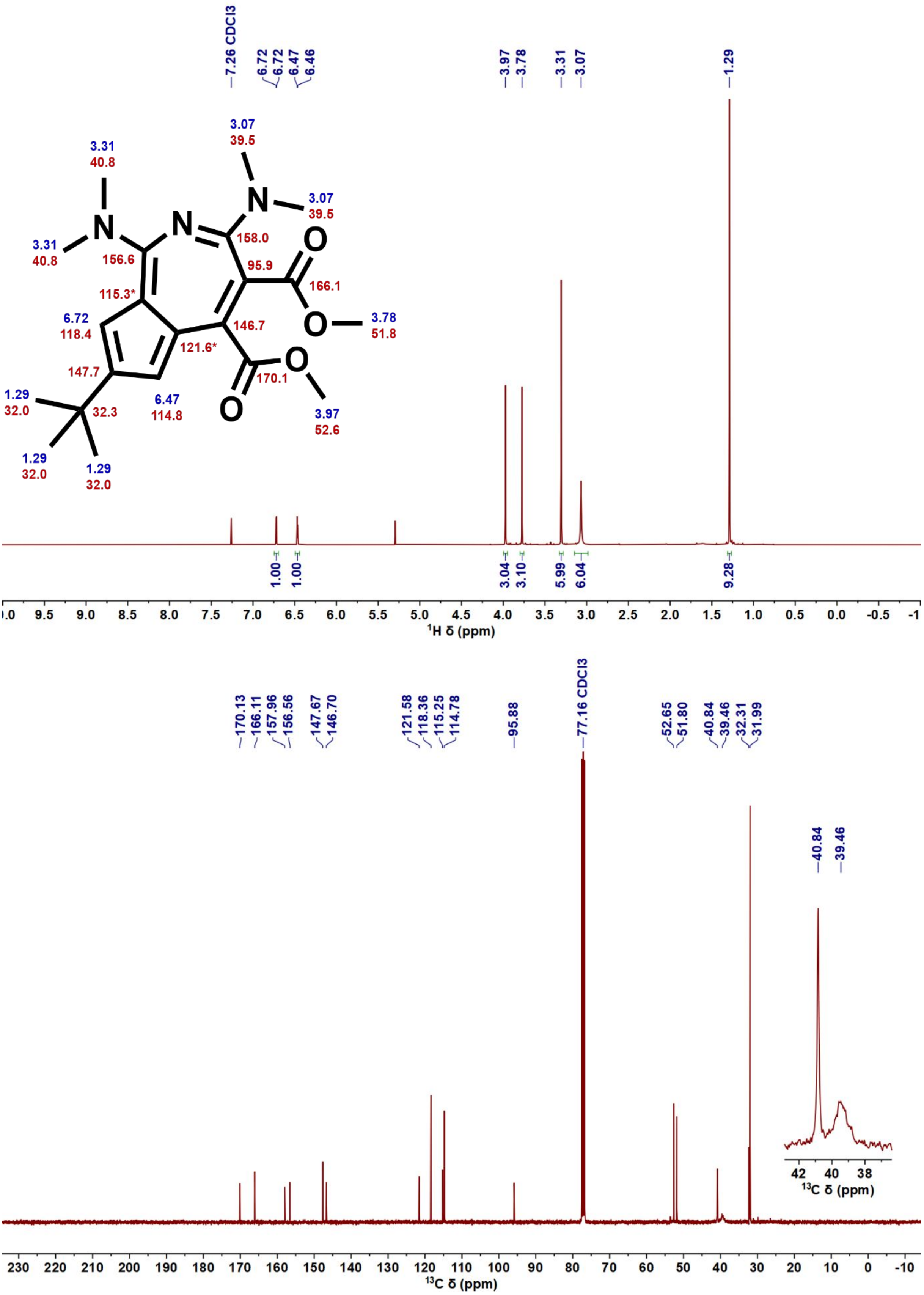

Figure S19. ^{1}H (400 MHz, top) and $^{13}C\{^{1}H\}$ (101 MHz, bottom) NMR spectra and assignments of **2** in $CDCl_3$.

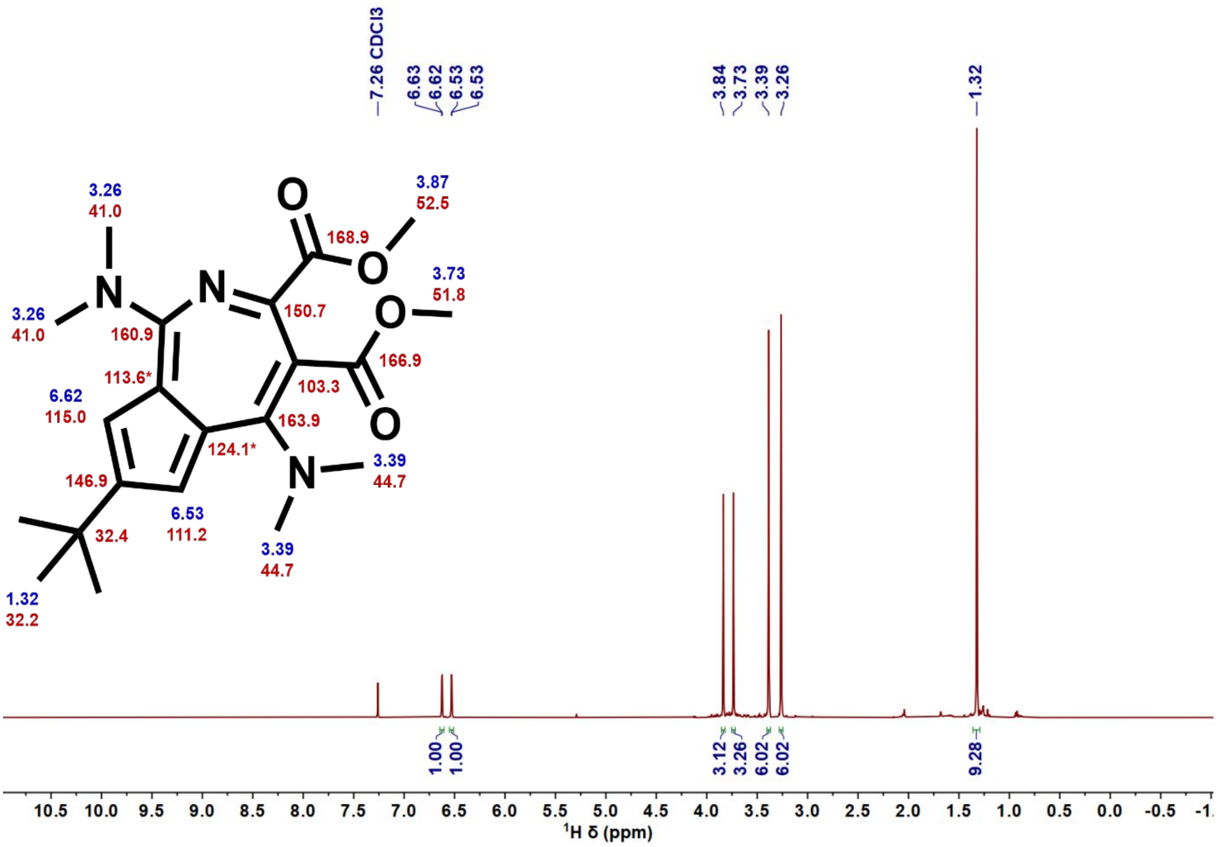

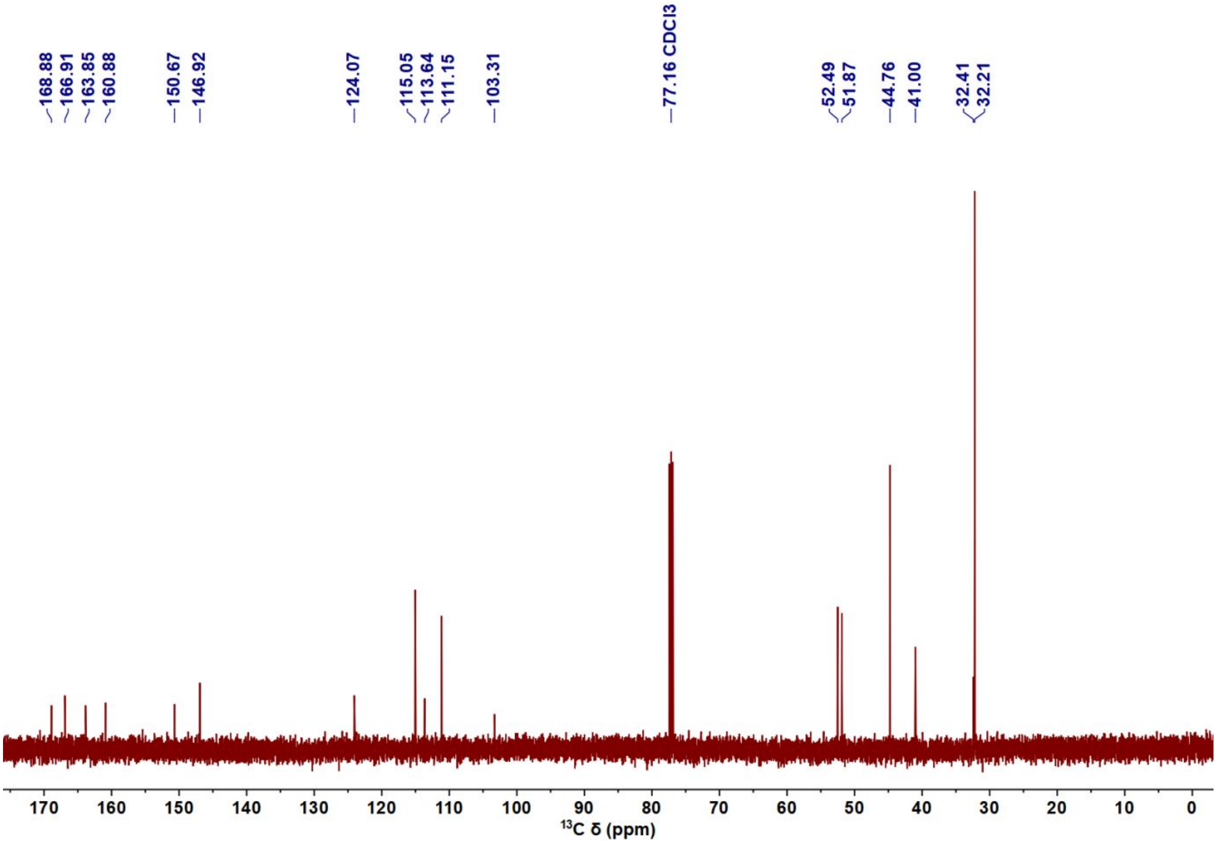

Figure S20. ^{1}H (500 MHz, top) and ^{13}C{^{1}H} (126 MHz, bottom) NMR spectra and assignments of **6** in $CDCl_3$.

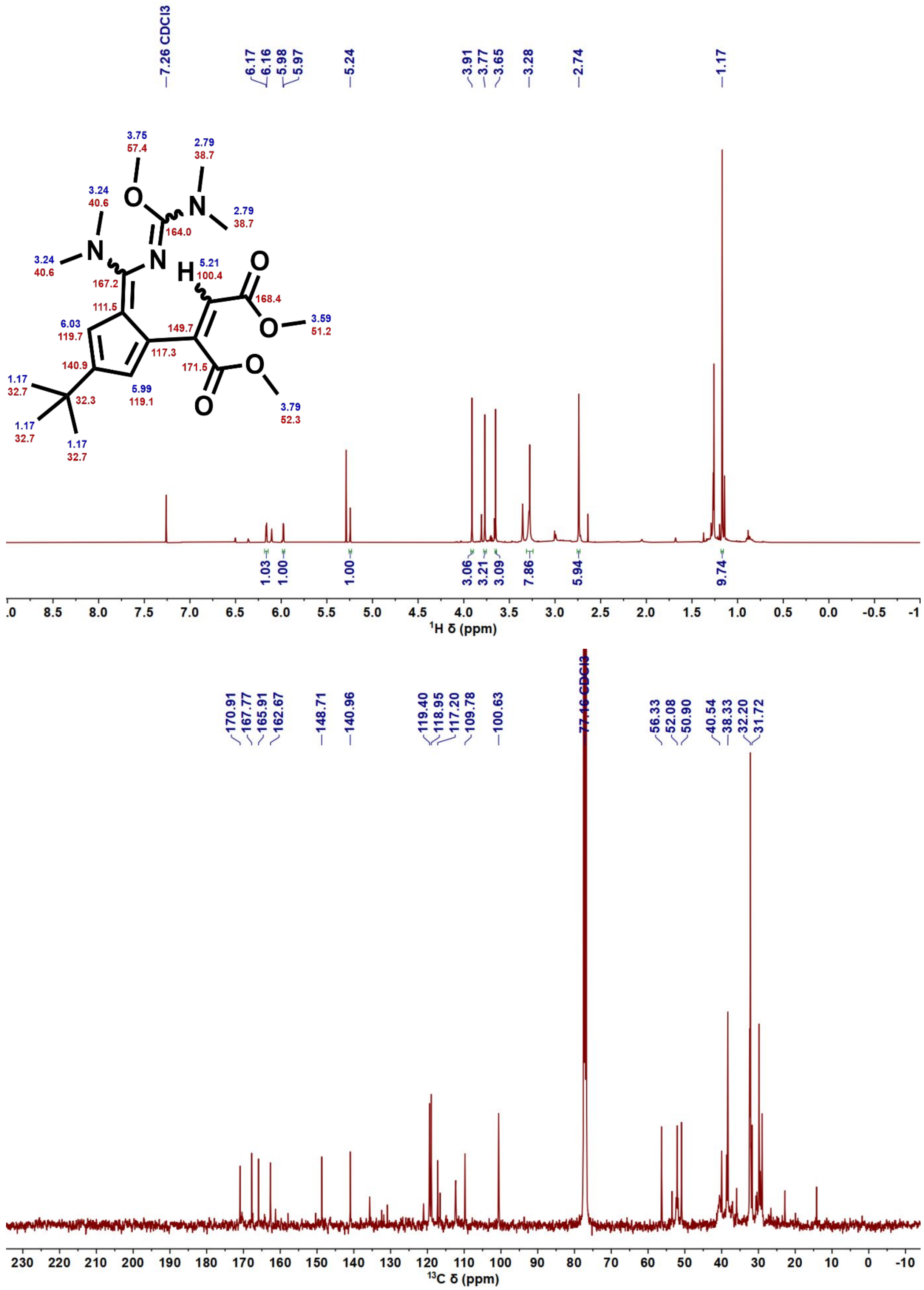

Figure S21. ^{1}H (400 MHz, top) and $^{13}C\{^{1}H\}$ (101 MHz, bottom) NMR spectra and assignments of **F1** in $CDCl_3$ at 50 °C.

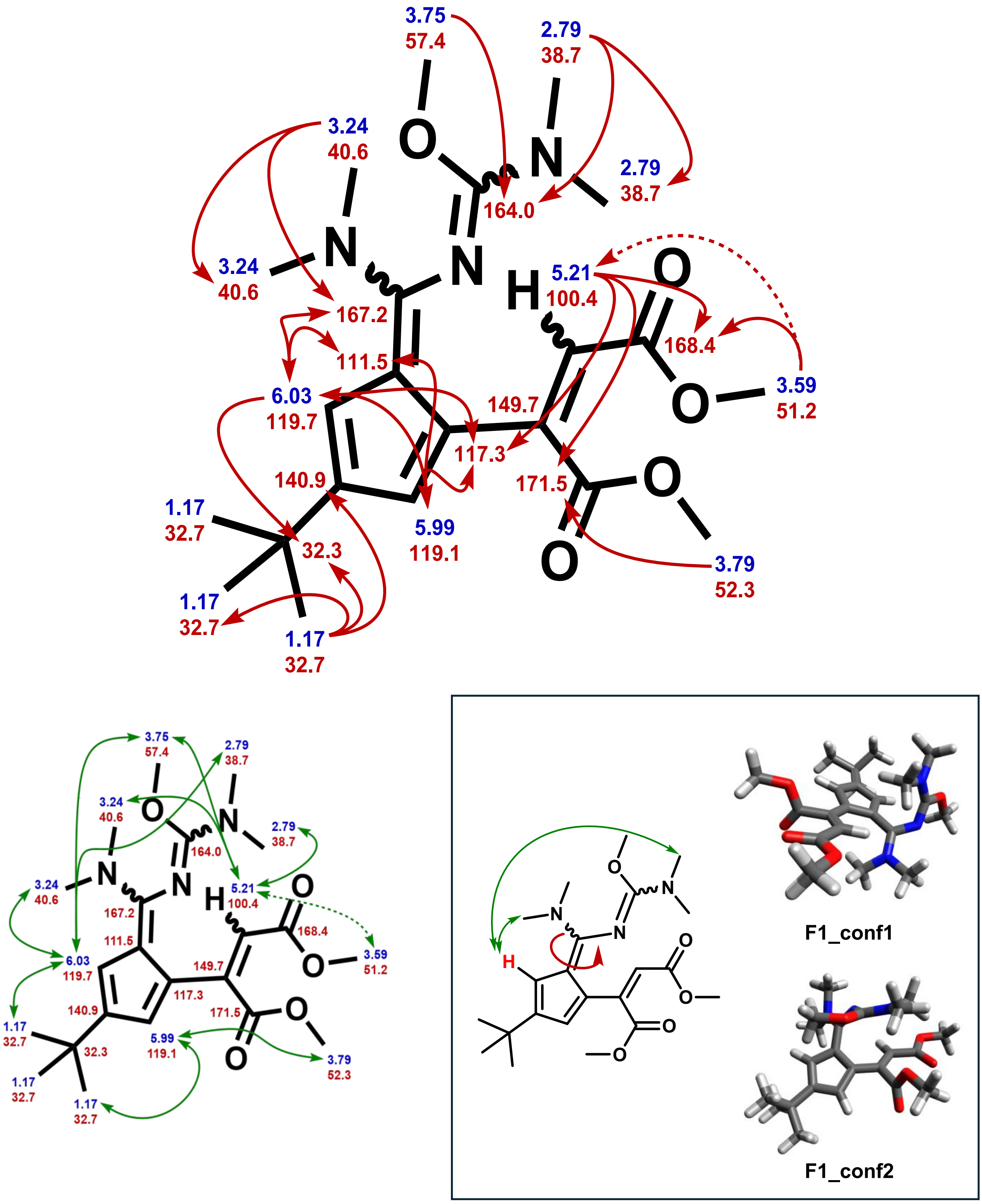

Figure S22. ^{1}H and $^{13}C\{^{1}H\}$ assignments of **F1**, indicating HMBC (top, maroon) and NOESY (bottom, green) correlations with arrows. Dashed arrows indicate weak correlations. Experiments indicate that both types of $NC\mathit{H}_3$ protons can be in contact with the highlighted CH proton of the cyclopentadiene. This suggests the presence of at least two distinct conformers, which is confirmed by DFT calculations: the rotation of the bond indicated in the figure requires only 4.8 kcal/mol activation free energy (i.e., single-bond-like rotation which

correlates with the C-C bond distance of ~1.5 Å) and leads to the two conformers shown with 2.5 kcal/mol energy difference between them. Note that we have performed conformational analysis and found numerous other structures within a few kcal/mol but did not explore other rotations that connect them to the structures shown.

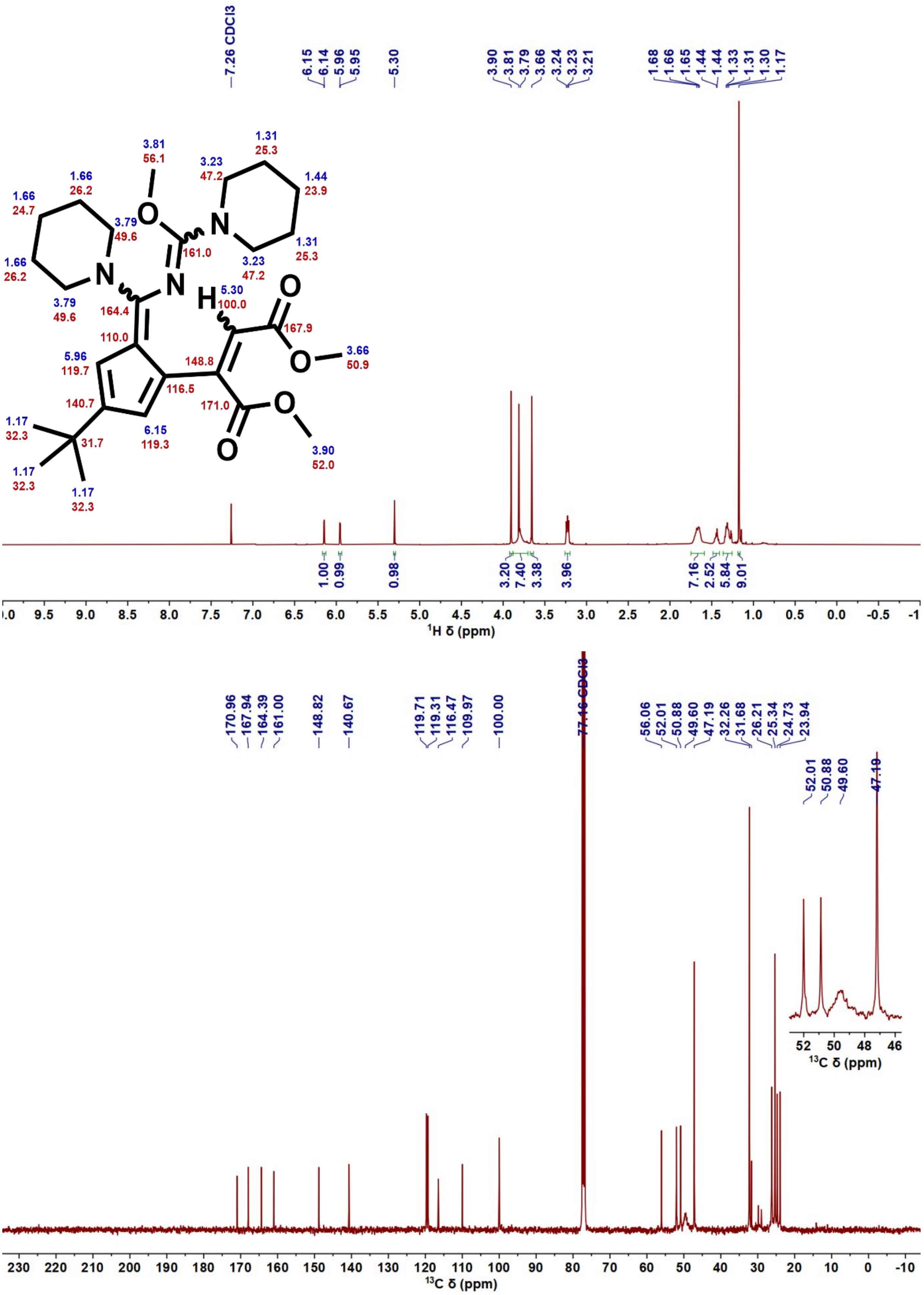

Figure S23. ^{1}H (400 MHz, top) and ^{13}C{^{1}H} (101 MHz, bottom) NMR spectra and assignments of **F2** in $CDCl_3$ at 50 °C.

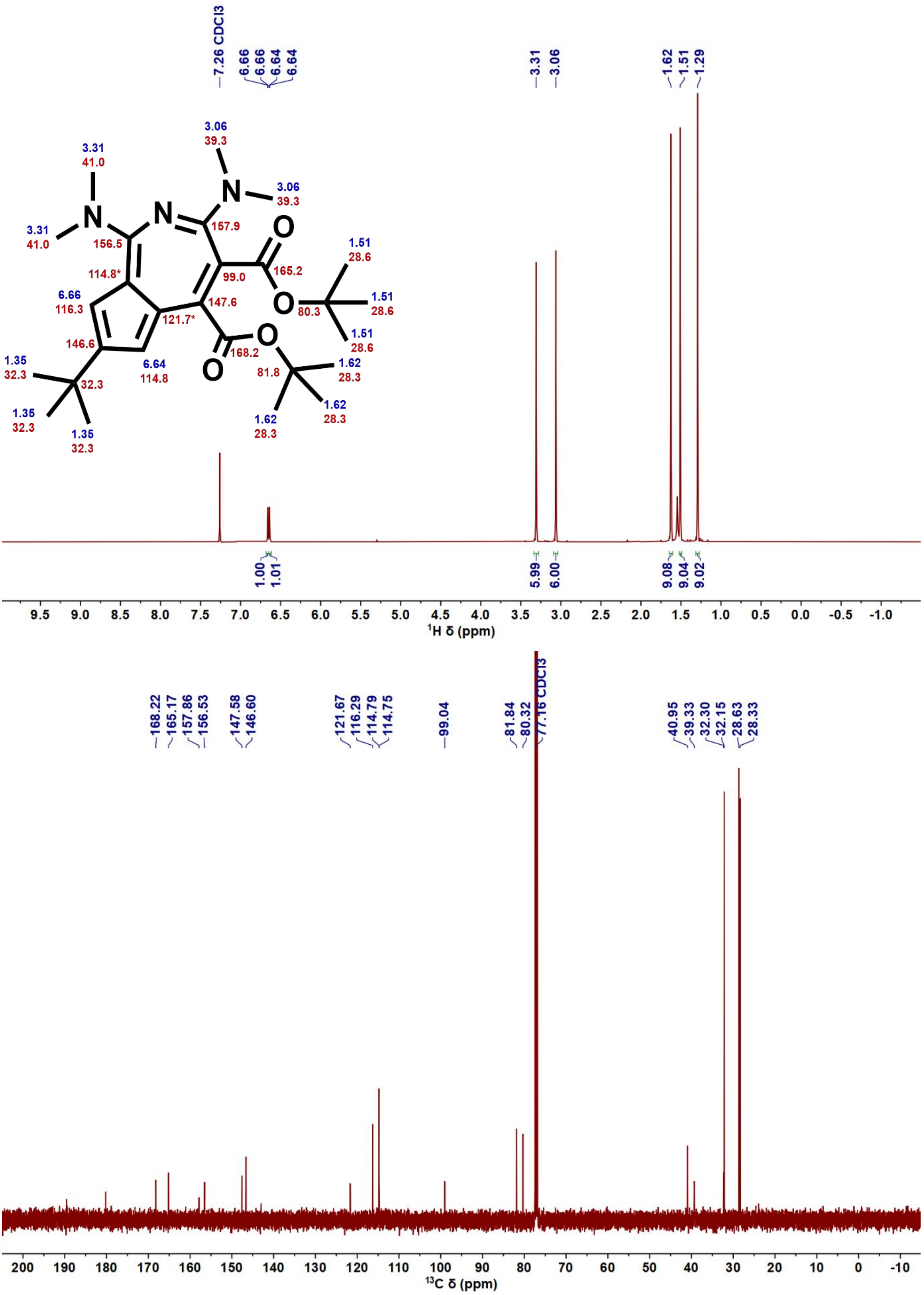

Figure S24. ^{1}H (500 MHz, top) and $^{13}C\{^{1}H\}$ (126 MHz, bottom) NMR spectra and assignments of **7** in $CDCl_3$.

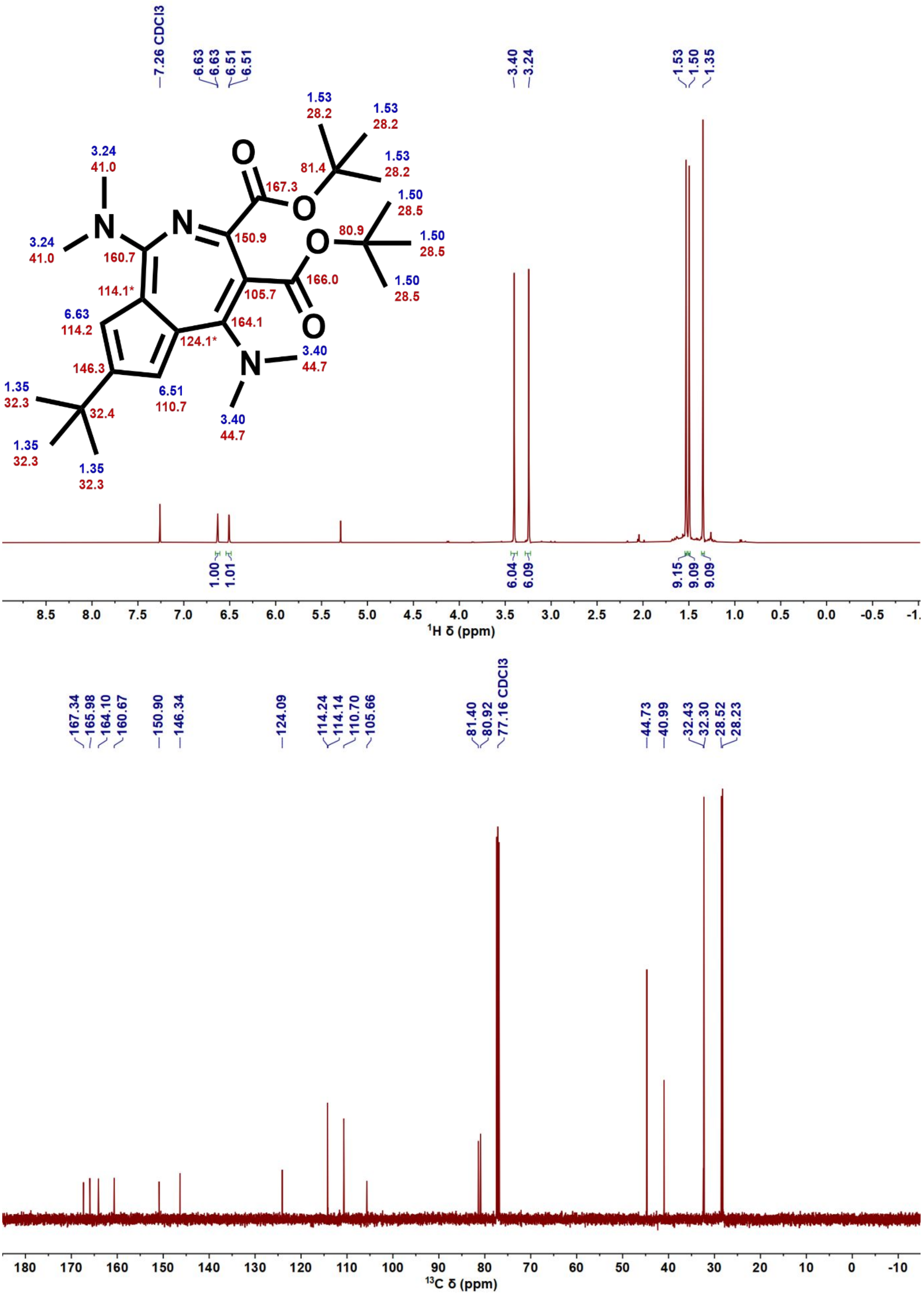

Figure S25. ^{1}H (500 MHZ, top) and $^{13}C\{^1H\}$ (126 MHz, bottom) NMR spectra and assignments of **8** in $CDCl_3$.

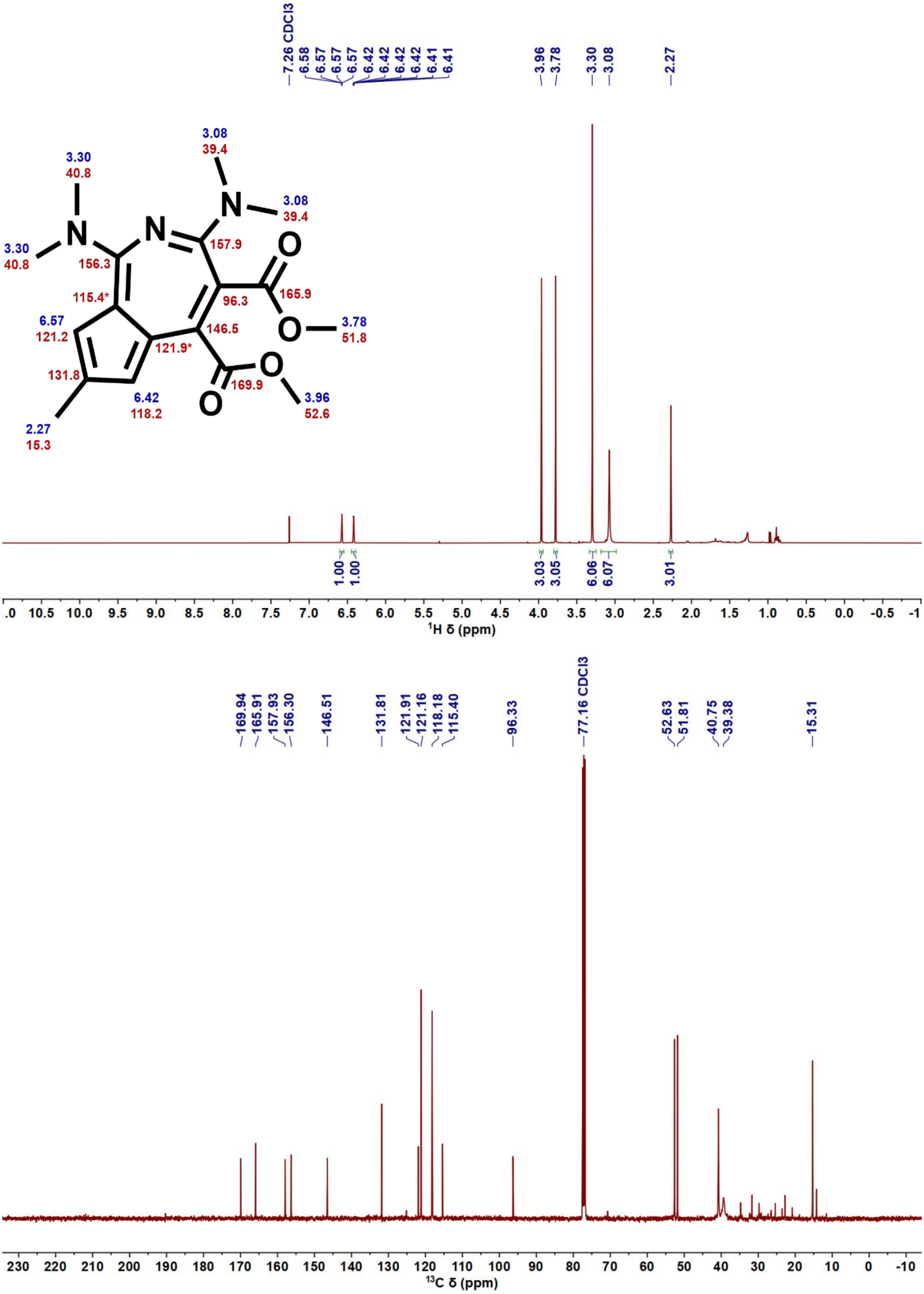

Figure S26. ^{1}H (400 MHz, top) and $^{13}C\{^{1}H\}$ (101 MHz, bottom) spectra and assignments of **9** in $CDCl_3$.

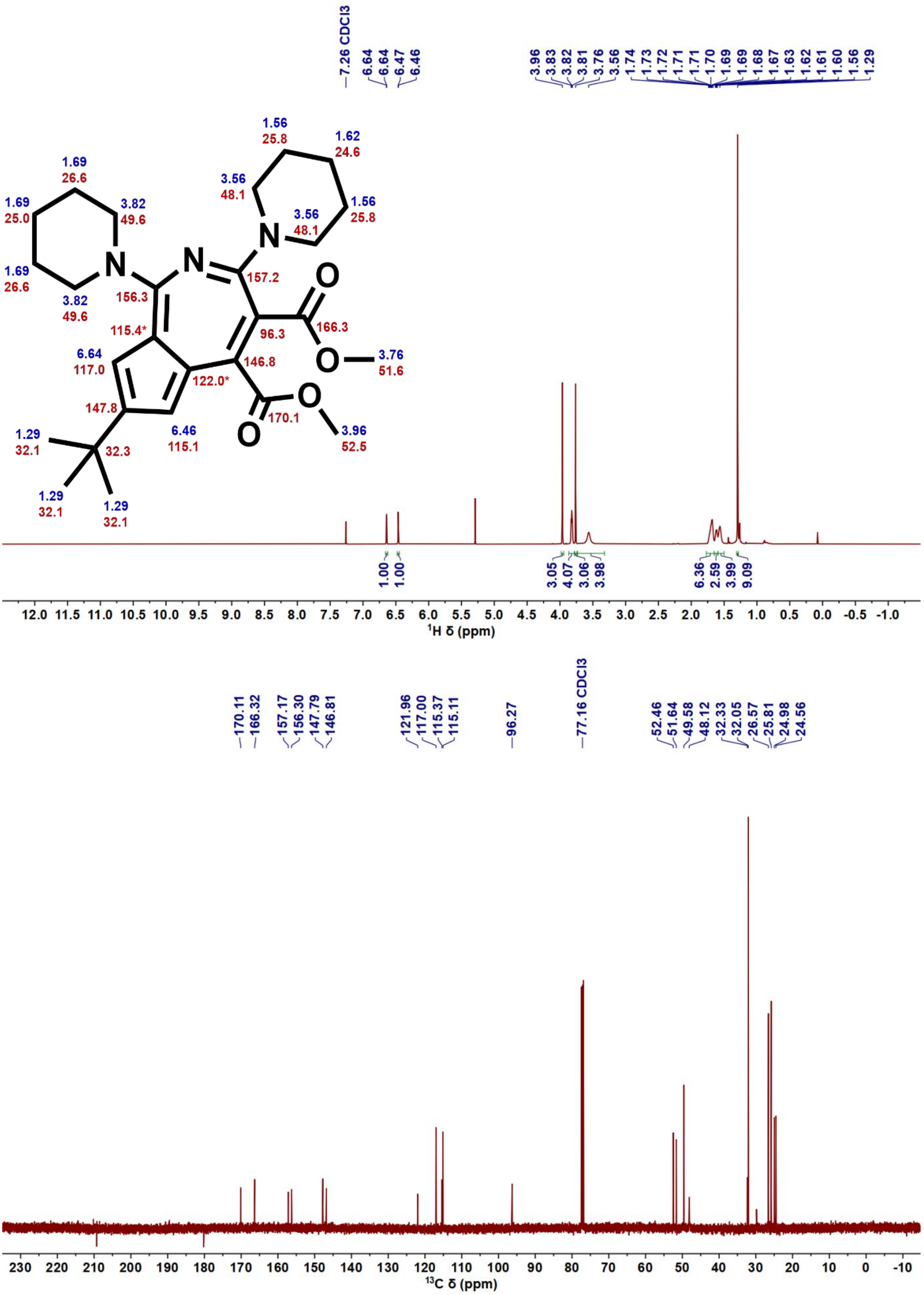

Figure S27. ^{1}H (500 MHz, top) and $^{13}C\{^{1}H\}$ (126 MHz, bottom) NMR spectra and assignments of **10** in $CDCl_3$ at 50 °C.

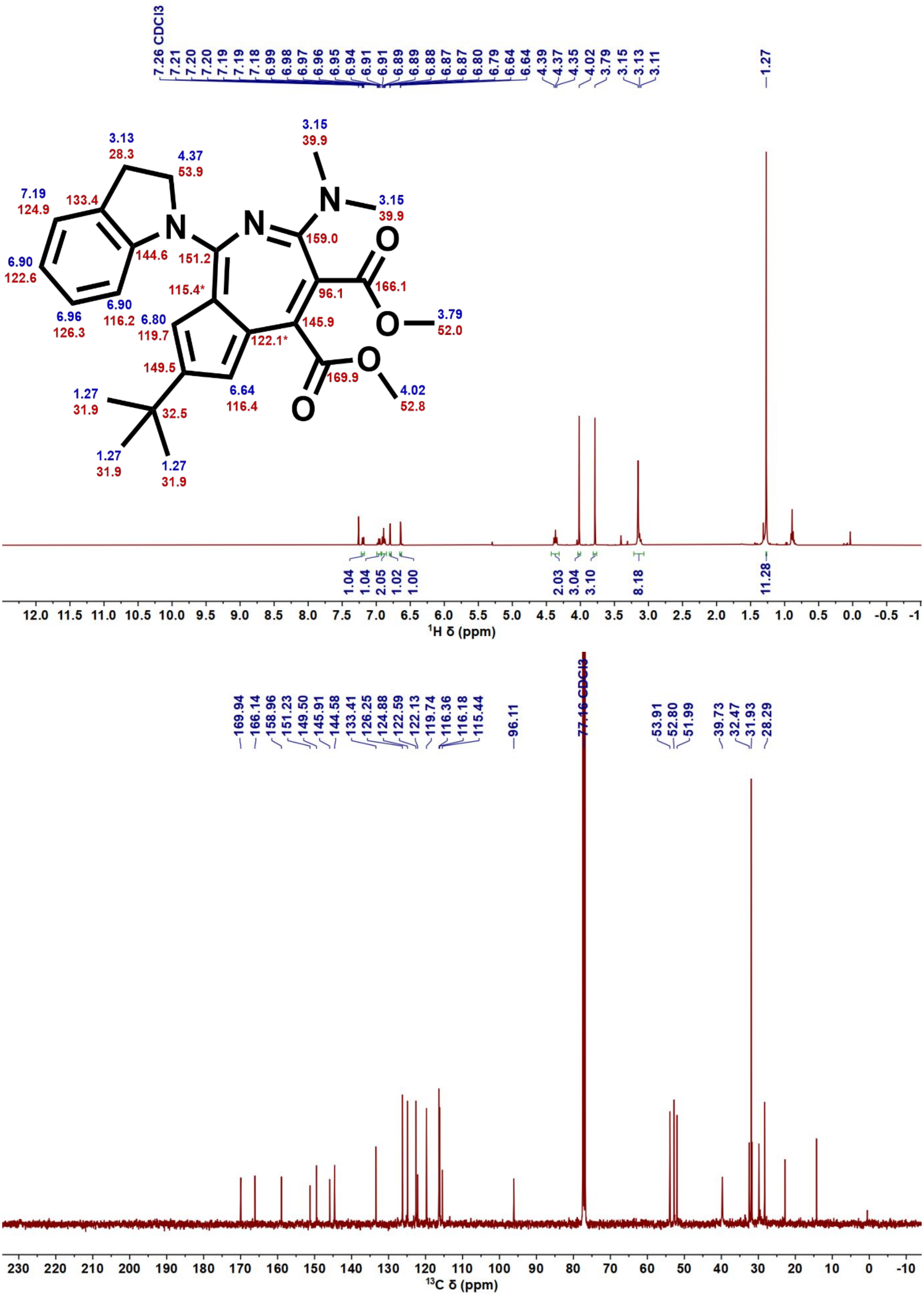

Figure S28. ^{1}H (400 MHz, top) and ^{13}C{^{1}H} (101 MHz, bottom) NMR spectra and assignments of **11** in $CDCl_3$.

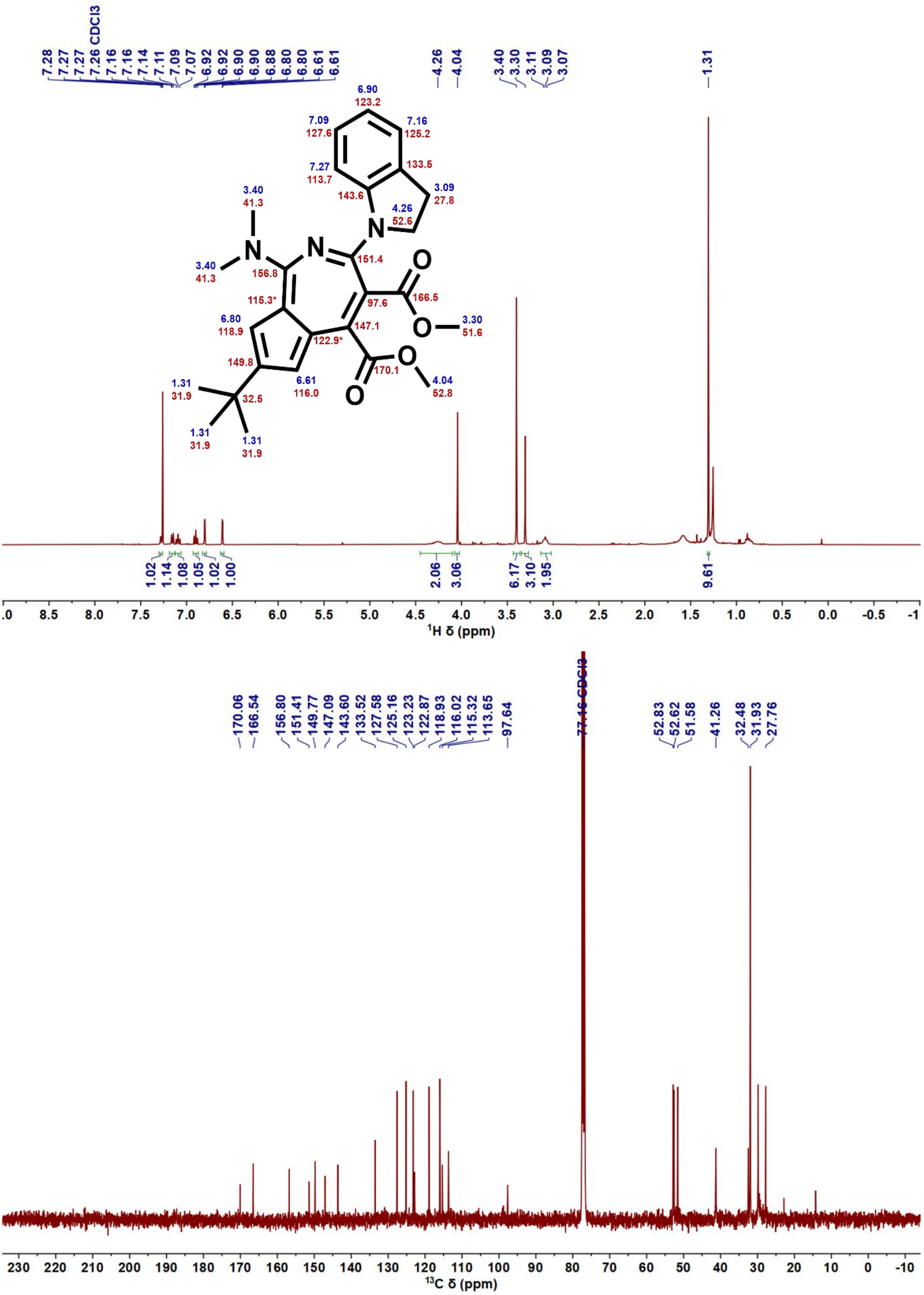

Figure S29. ^{1}H (400 MHz, top) and $^{13}C\{^{1}H\}$ (101 MHz, bottom) spectra and assignments of **12** in $CDCl_3$.

S8 HRMS spectra

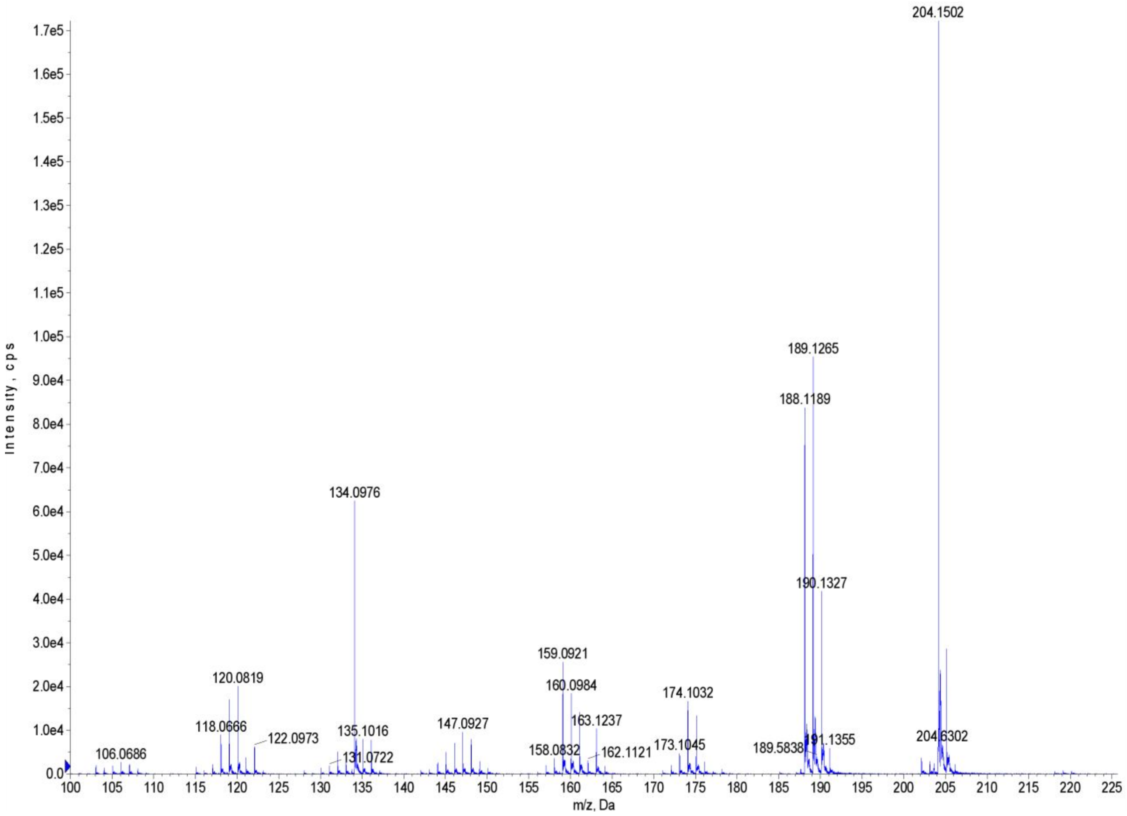

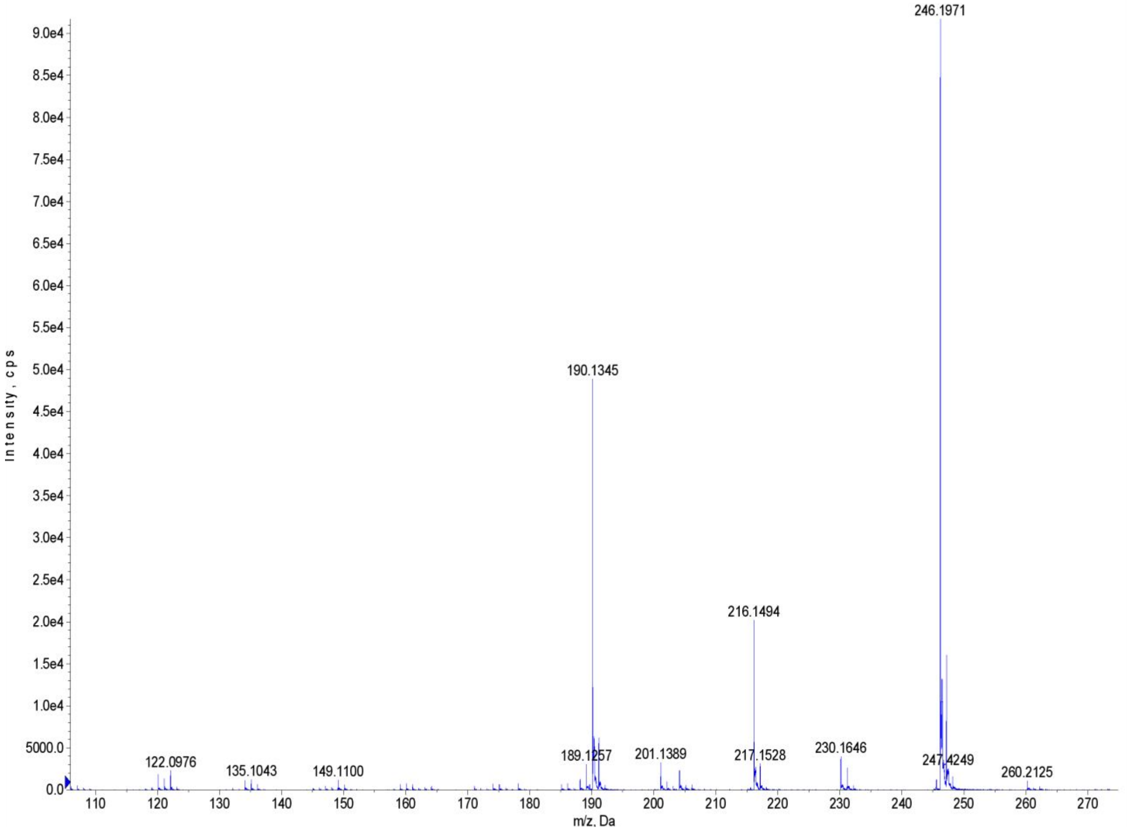

Figure S30. HRMS spectra of **S1** (top) and **1** (bottom).

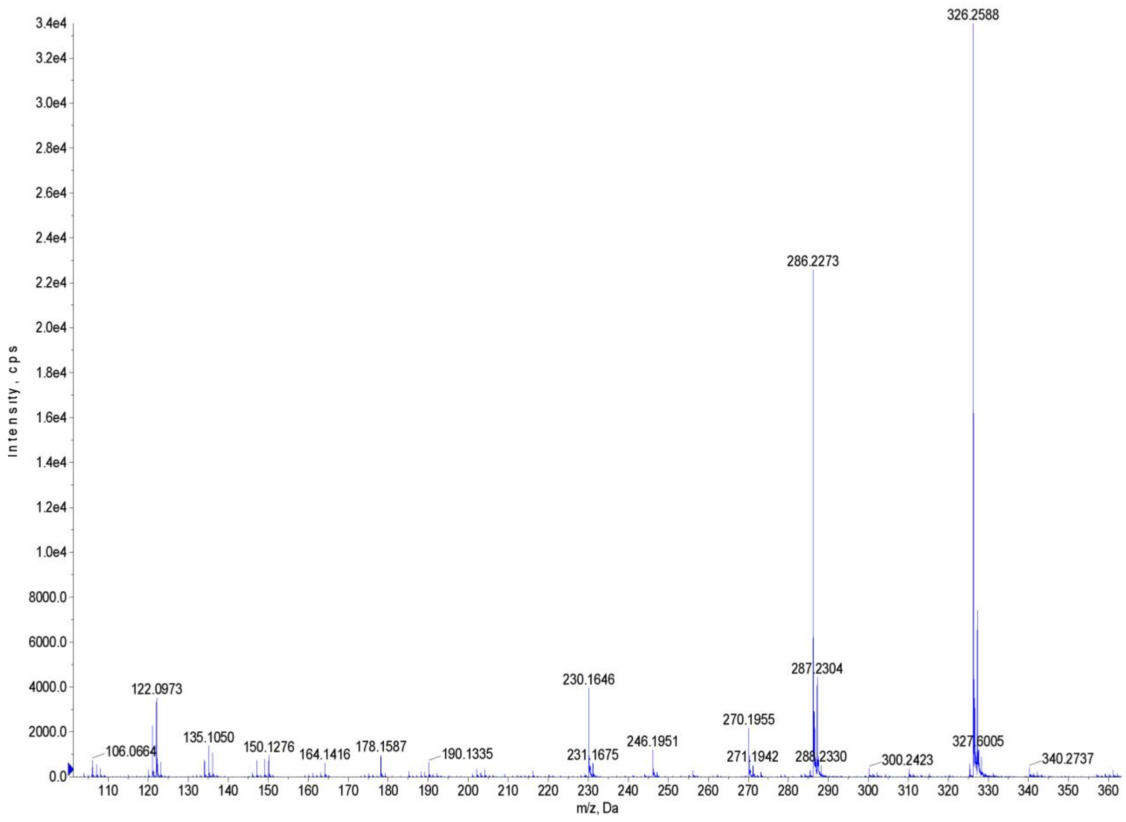

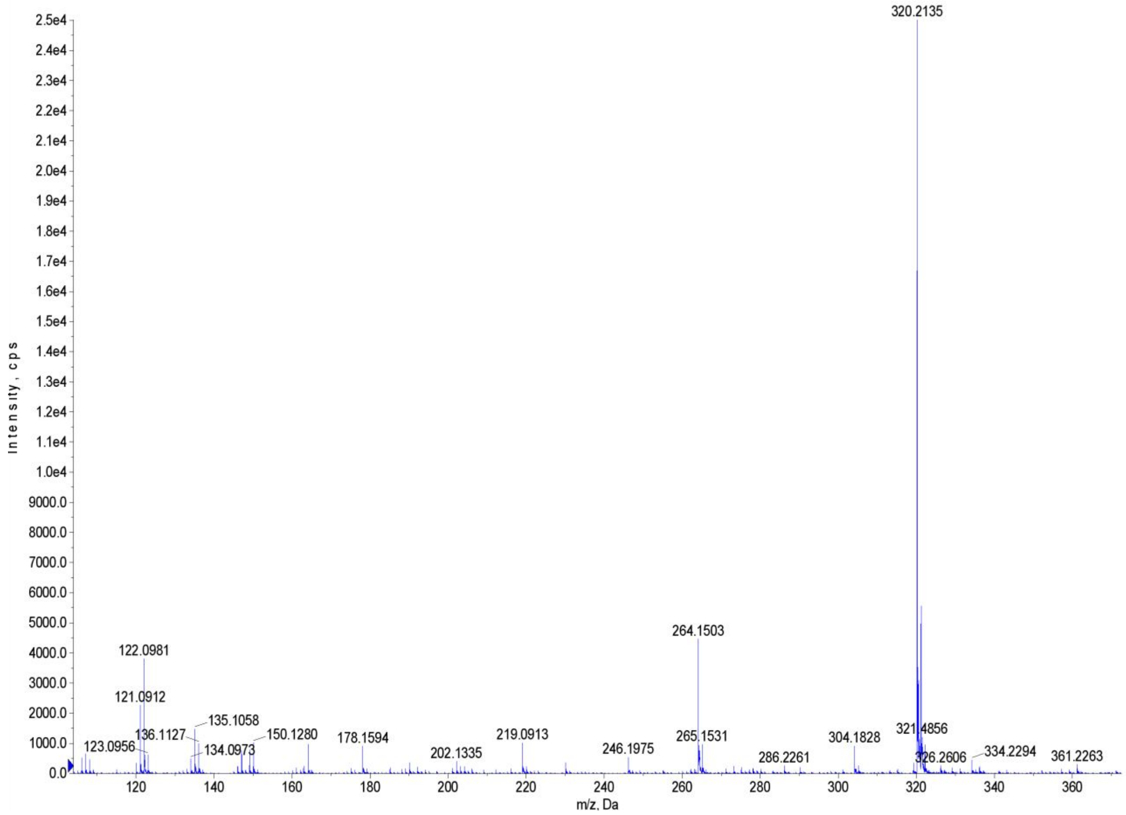

Figure S31. HRMS spectra of **S2** (top) and **S3** (bottom).

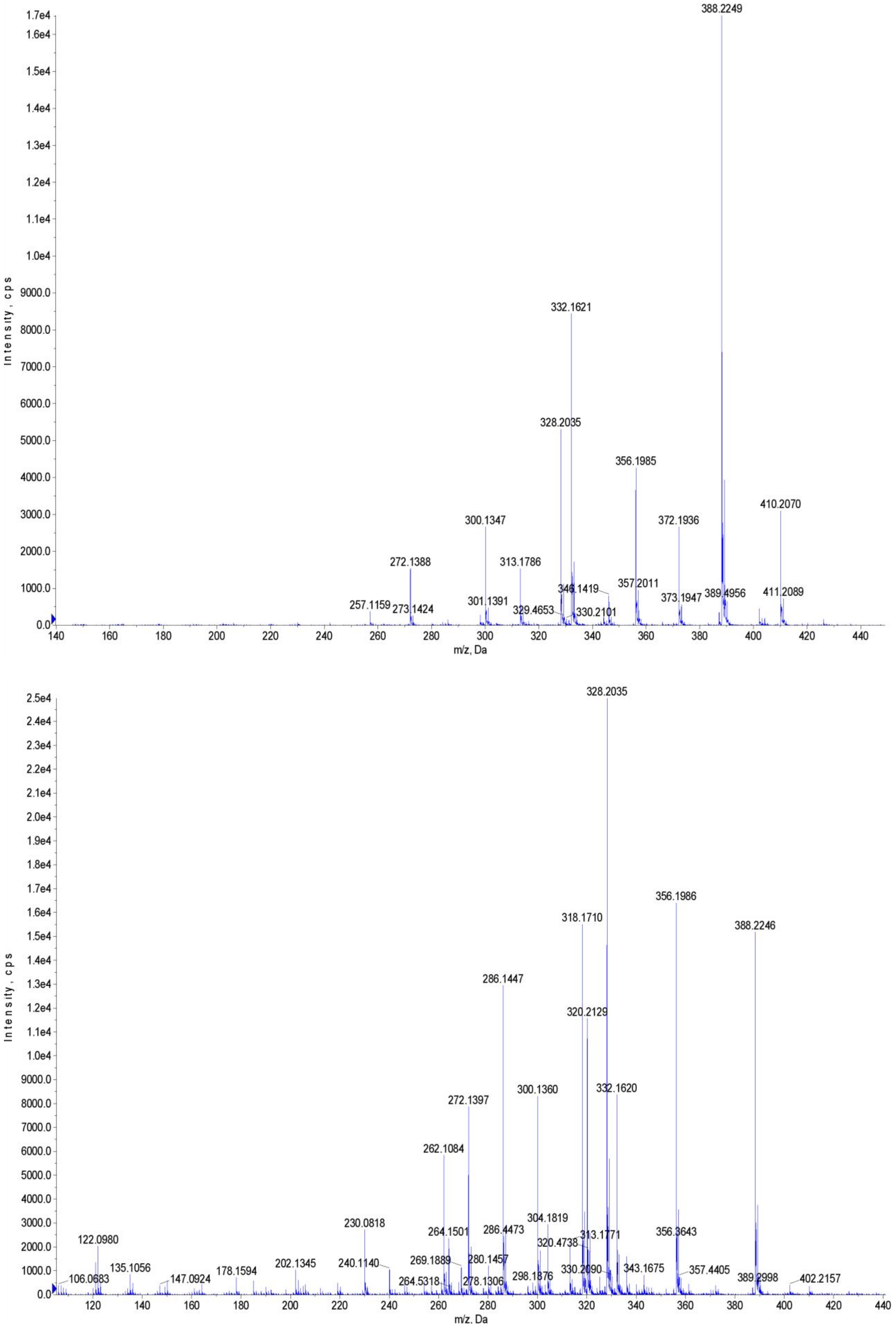

Figure S32. HRMS spectra of **2** (top) and **6** (bottom).

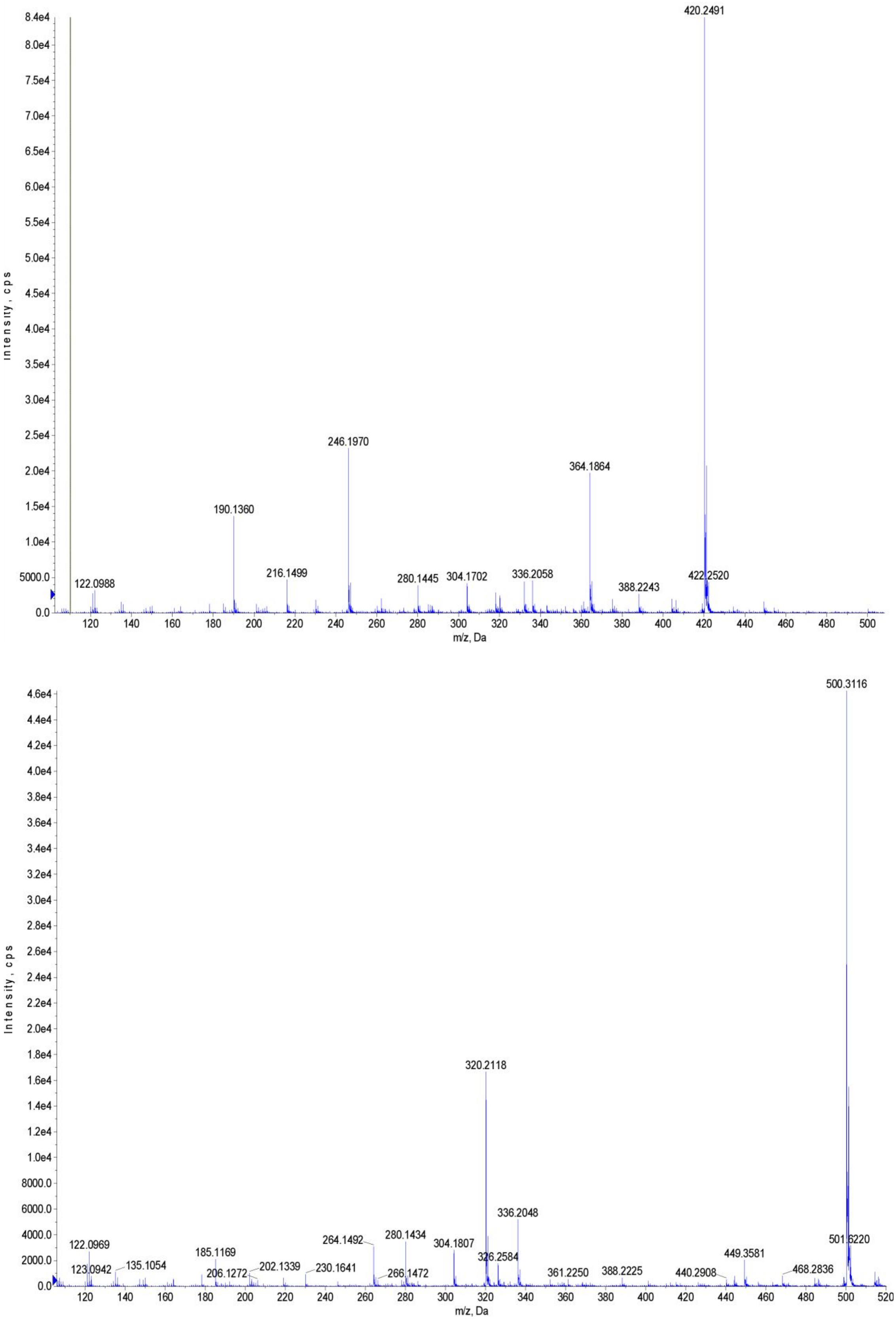

Figure S33. HRMS spectra of **F1** (top) and **F2** (bottom).

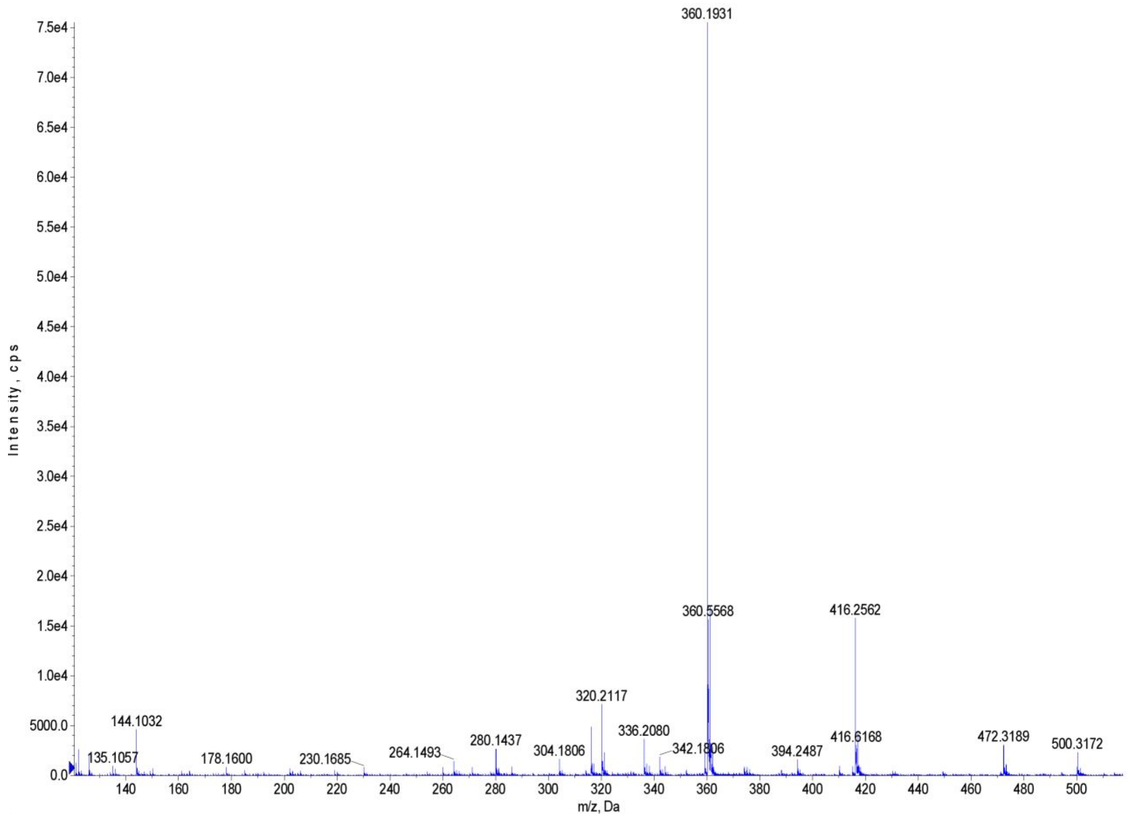

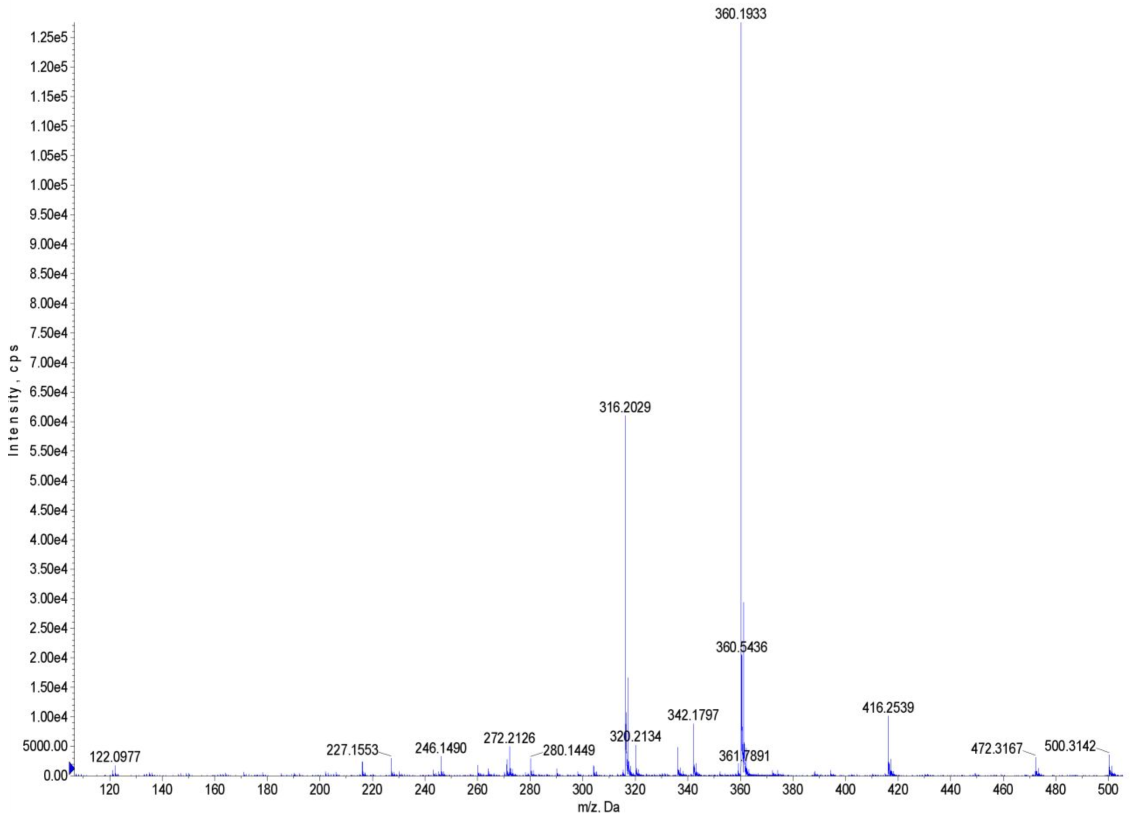

Figure S34. HRMS spectra of **7** (top) and **8** (bottom).

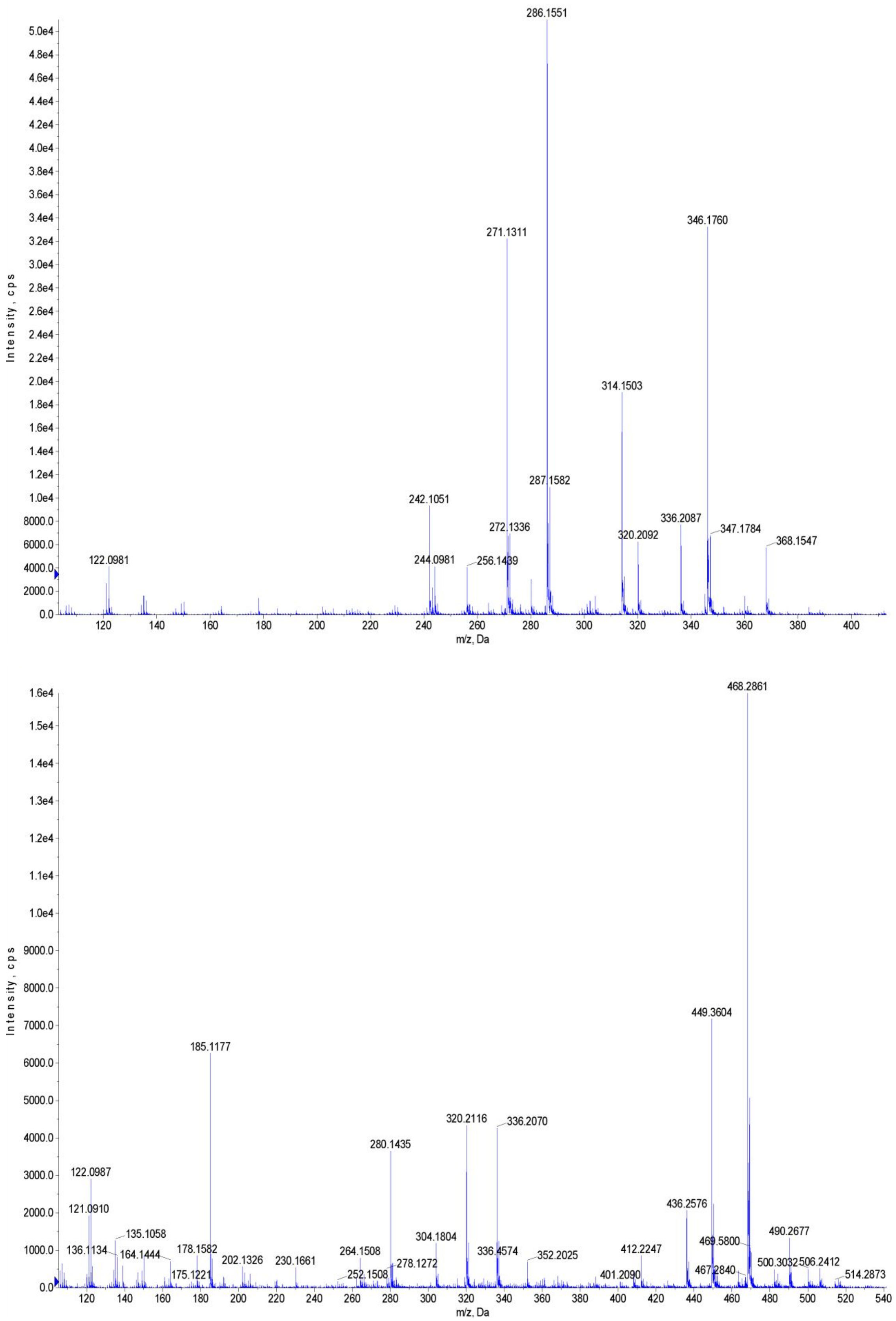

Figure S35. HRMS spectra of **9** (top) and **10** (bottom).

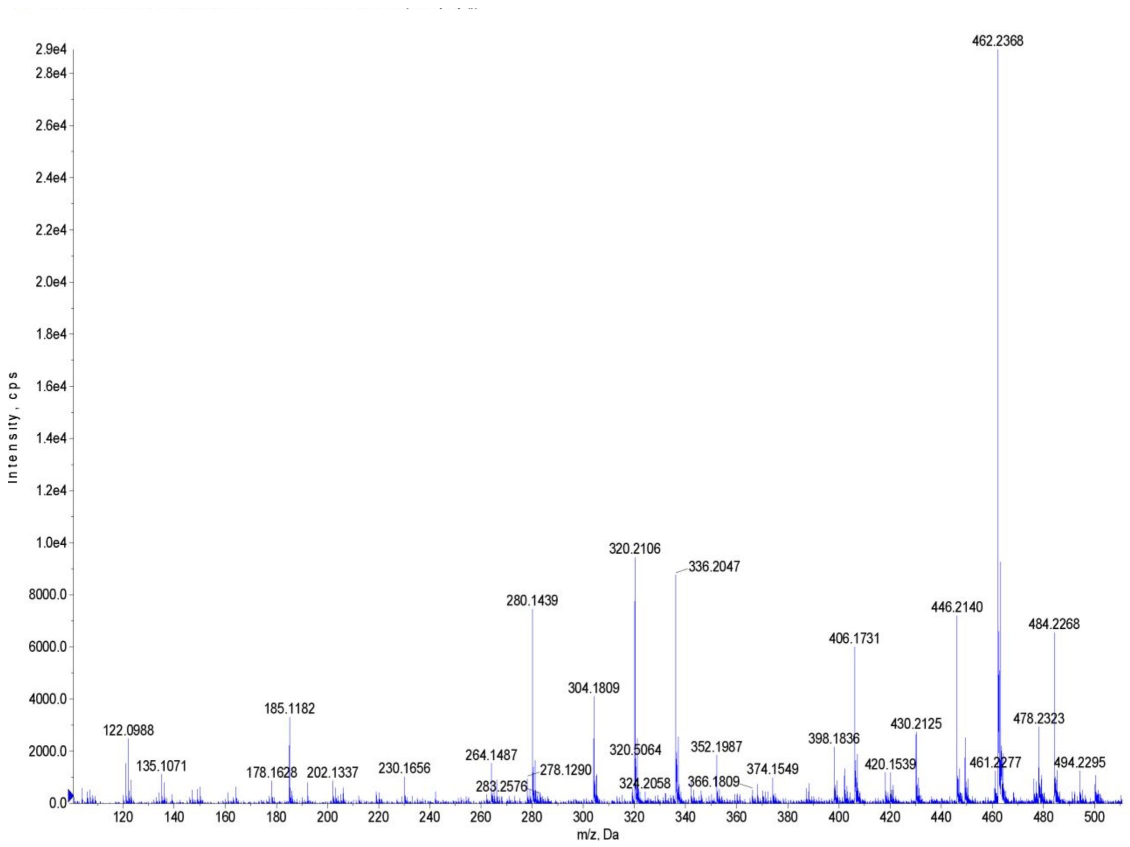

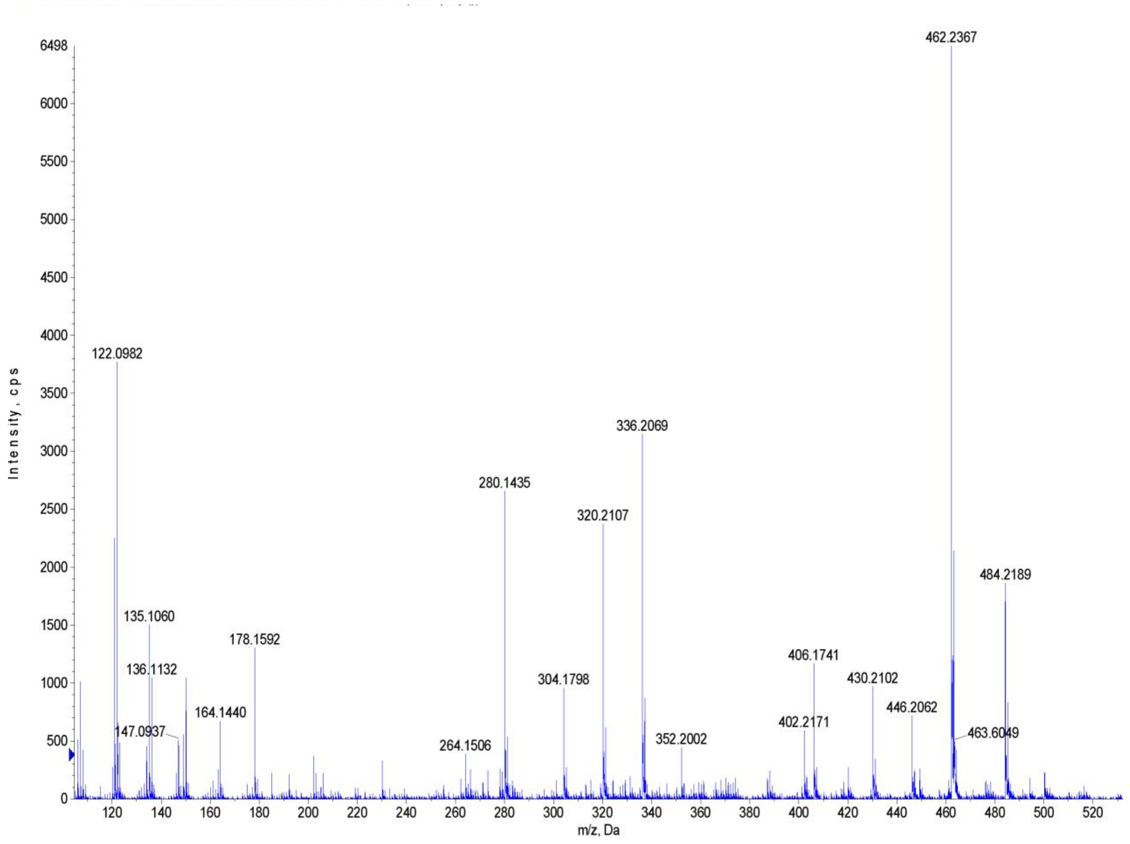

Figure S36. HRMS spectra of **11** (top) and **12** (bottom).

S9 Cartesian coordinates and free energies in XYZ format

All coordinates are given in Angstroms, while the free energies are in Hartrees.

```
41
1, G = -749.881172 a.u.
C      -0.614175     -0.922427     -0.350471
C       1.185792      0.228667      0.015481
C       0.112381      1.212195     -0.054101
C      -1.059369      0.458651     -0.291883
C      -0.229062      2.576118     -0.029894
C      -1.631998      2.660881     -0.251688
C      -2.145528      1.350546     -0.418420
H      -3.185548      1.096808     -0.611314
C      -2.424112      3.962981     -0.290603
C      -3.903200      3.694464     -0.587209
H      -4.466361      4.639343     -0.618055
H      -4.037598      3.195348     -1.558029
H      -4.362779      3.059361      0.184112
C      -1.860729      4.884793     -1.384824
H      -1.930558      4.411470     -2.375770
H      -2.414638      5.836009     -1.424581
H      -0.802800      5.123594     -1.202729
C      -2.314793      4.677917      1.066802
H      -2.716801      4.051010      1.876913
H      -1.268458      4.910874      1.313080
H      -2.875639      5.626044      1.062564
N       0.728694     -1.034229     -0.166743
H       0.437936      3.423424      0.123993
N       2.466690      0.492237      0.229506
N      -1.383561     -1.981788     -0.556212
C      -0.841256     -3.327413     -0.646493
H      -1.057332     -3.756126     -1.635877
H       0.240084     -3.293392     -0.495644
H      -1.296612     -3.970785      0.119847
C      -2.814360     -1.811091     -0.748828
H      -3.033293     -1.325905     -1.711608
H      -3.295019     -2.794846     -0.736576
H      -3.244678     -1.203759      0.058101
C       3.479379     -0.550628      0.239303
H       4.010788     -0.551361      1.201565
H       3.001915     -1.521450      0.088080
H       4.210476     -0.376185     -0.563530
C       2.903887      1.866995      0.413013
H       2.818492      2.441333     -0.521222
H       2.306667      2.364201      1.188403
H       3.952132      1.867395      0.729033

57
2, G = -1283.299474 a.u.
C      -0.729208     -0.004399     -0.158767
C       1.069565      1.112266      0.981478
C      -1.804913      0.998175     -0.076566
C       0.970074      2.424222      0.375350
C      -1.531437      2.343267     -0.187314
C      -0.251116      2.959989     -0.186155
C       1.956108      3.412549      0.249949
H       2.993734      3.315771      0.563557
C       1.391691      4.551723     -0.378363
C       0.038437      4.283940     -0.612126
H      -0.680057      4.954263     -1.078886
C       2.155611      5.823083     -0.718144
C       1.236561      6.849305     -1.387649
H       1.796563      7.764763     -1.630247
H       0.811160      6.460929     -2.324509
H       0.401187      7.134476     -0.731591
C       3.311269      5.488858     -1.675497
H       2.935299      5.050545     -2.612078
H       3.884171      6.393824     -1.931297
H       4.009532      4.768440     -1.225225
C       2.732624      6.434677      0.569254
H       3.297908      7.353720      0.349043
H       1.931723      6.692029      1.278556
H       3.415435      5.736065      1.074305
N       0.403208      0.066464      0.505485
N       1.929559      0.851249      1.982850
N      -0.915232     -1.102279     -0.911719
C       2.574349      1.868732      2.799255
H       2.529278      1.552823      3.849861
H       3.631013      2.004817      2.524971
H       2.056345      2.826510      2.706258
C       2.147043     -0.528421      2.395609
H       3.059787     -0.573690      2.999846
H       1.306582     -0.914216      2.992780
H       2.268847     -1.174724      1.518757
C       0.066743     -2.174767     -0.853396
H       0.290337     -2.431408      0.189638
H      -0.345255     -3.056067     -1.356394
H       1.008987     -1.891216     -1.345385
C      -2.003967     -1.269116     -1.859833
H      -1.595270     -1.656921     -2.802394
H      -2.761539     -1.974796     -1.490435
H      -2.481949     -0.309299     -2.081007
C      -3.202466      0.563922      0.146881
O      -4.188465      1.223909     -0.101709
O      -3.269416     -0.650813      0.706917
C      -2.676675      3.319720     -0.339188
O      -3.033819      3.769684     -1.397321
O      -3.148347      3.694352      0.846685
C      -4.269325      4.577092      0.818059
H      -5.110318      4.112778      0.286542
H      -4.543889      4.761100      1.861746
H      -4.011658      5.525578      0.328220
C      -4.578171     -1.173591      0.927616
H      -4.441501     -2.182558      1.329923
H      -5.129265     -0.556660      1.649852
H      -5.148245     -1.219207     -0.009787

57
6, G = -1283.280986 a.u.
C      -0.793374      0.101797     -0.278068
C       1.316422      1.019089      0.327085
C      -1.745780      1.072238     -0.038990
C       0.999494      2.408349      0.054170
C      -1.464846      2.321274      0.687864
C      -0.270565      3.036233      0.340630
C       1.875840      3.419990     -0.370750
H       2.921498      3.272395     -0.635457
C       1.184436      4.651944     -0.392568
C      -0.116272      4.422191      0.082936
H      -0.907168      5.163634      0.155171
C       1.775051      5.978629     -0.848929
C       3.009249      6.312741      0.005042
H       3.781522      5.534880     -0.083527
H       3.459006      7.267169     -0.310374
H       2.742386      6.400032      1.069011
C       0.752783      7.110699     -0.706268
H       1.190549      8.066136     -1.031891
H      -0.141439      6.929028     -1.320143
H       0.426484      7.232805      0.336997
C       2.199705      5.873615     -2.322796
H       1.338690      5.640299     -2.966924
H       2.638863      6.820109     -2.675154
H       2.950080      5.082783     -2.468050
N       0.484397      0.009431      0.095373
N       2.551162      0.667519      0.754627
C       2.931354     -0.735978      0.790262
H       2.562311     -1.237335      1.699714
H       2.525001     -1.265089     -0.077568
H       4.026115     -0.803594      0.774176
C       3.479223      1.571862      1.415501
H       3.861003      1.082399      2.322890
```

H	4.338004	1.821406	0.774363
H	2.979019	2.497579	1.710695
C	-3.061464	0.913695	-0.699909
O	-3.540376	-0.134307	-1.075237
O	-3.666261	2.094254	-0.895849
N	-2.338580	2.768291	1.579221
C	-2.173905	4.045303	2.264475
H	-1.111279	4.267764	2.402004
H	-2.638960	4.859543	1.692425
H	-2.663258	3.982107	3.242094
C	-3.534315	2.030010	1.973177
H	-3.629510	2.092758	3.063634
H	-4.434296	2.462433	1.516924
H	-3.452926	0.974049	1.700725
C	-1.168556	-1.138218	-1.073752
O	-1.016485	-1.225584	-2.264957
O	-1.533565	-2.136836	-0.276399
C	-4.960965	2.050523	-1.493560
H	-4.905895	1.655345	-2.516553
H	-5.646353	1.425561	-0.905853
H	-5.324076	3.083129	-1.514198
C	-1.862264	-3.362608	-0.928416
H	-2.139427	-4.068507	-0.138686
H	-2.707884	-3.221427	-1.614551
H	-1.003772	-3.753100	-1.491159

65

11

C	-1.280801	-0.014706	0.061808
C	0.932034	0.858288	-0.177325
C	-1.778022	0.712508	1.239440
C	0.661523	2.257577	0.006767
C	-1.414323	2.019916	1.486427
C	-0.432017	2.778948	0.799349
C	1.427334	3.345384	-0.426962
H	2.325766	3.276439	-1.038073
C	0.855247	4.537370	0.077585
C	-0.262593	4.186623	0.848343
H	-0.928429	4.873397	1.366204
C	1.401957	5.934311	-0.173616
C	1.429805	6.214999	-1.684441
H	1.832251	7.219274	-1.889083
H	2.060632	5.488034	-2.216256
H	0.419301	6.161363	-2.116476
C	2.833911	6.022716	0.381029
H	3.261213	7.023058	0.209973
H	2.850925	5.828168	1.463953
H	3.496990	5.288924	-0.100008
C	0.534754	6.992565	0.514984
H	-0.499039	6.974507	0.140306
H	0.501140	6.842494	1.603913
H	0.939114	7.998894	0.330200
N	-0.002822	-0.061693	-0.285684
N	-2.125565	-0.745773	-0.672286
C	-1.600130	-1.599092	-1.730334
H	-2.388943	-2.289420	-2.046682
H	-1.272701	-1.008350	-2.598070
H	-0.743253	-2.176187	-1.362468
C	-3.572885	-0.700995	-0.536813
H	-3.958821	-1.601940	-0.040302
H	-3.886386	0.186019	0.023044
H	-4.015818	-0.634615	-1.538949
C	-2.668524	0.021188	2.201140
O	-3.402938	0.571589	2.992317
O	-2.546552	-1.309848	2.131555
C	-2.113059	2.785820	2.588464
O	-3.007952	3.567101	2.394271
O	-1.531334	2.567358	3.764065
C	-2.139160	3.206374	4.886412
H	-3.179594	2.874924	4.999971
H	-1.555526	2.909401	5.763649
H	-2.117311	4.298747	4.776975
C	-3.396658	-2.068489	2.990832
H	-3.209507	-3.121108	2.755580
H	-3.159225	-1.874449	4.045214
H	-4.452872	-1.827262	2.812567
C	3.422061	0.935890	0.128766
C	2.480924	-0.875792	-1.028786
C	4.492922	0.238244	-0.446626
C	3.637828	1.890759	1.121253
C	3.960245	-0.782104	-1.421040
H	2.305427	-1.693614	-0.312285
H	1.805128	-1.017575	-1.878552
C	5.799364	0.531337	-0.079370
C	4.957531	2.175660	1.483089
H	2.812046	2.402665	1.609169
H	4.470700	-1.750002	-1.349304
C	6.032496	1.515739	0.886460
H	6.631810	-0.010065	-0.532893
H	5.142241	2.924589	2.255505
H	7.053939	1.754673	1.186871
N	2.206484	0.422012	-0.386700
H	4.073615	-0.420967	-2.455166

65

12

C	-0.549130	-0.208704	0.360849
C	0.413220	1.308004	1.957146
C	-1.425708	0.673266	-0.408424
C	0.701059	2.442409	1.109358
C	-1.058044	1.972530	-0.700516
C	0.021599	2.701586	-0.145440
C	1.620984	3.479175	1.307332
H	2.303141	3.568875	2.150220
C	1.544926	4.380857	0.214262
C	0.548479	3.920927	-0.650639
H	0.238076	4.390299	-1.581700
C	2.410414	5.621247	0.052129
C	2.203257	6.552172	1.258069
H	1.152653	6.869254	1.337454
H	2.475608	6.055547	2.200664
H	2.823952	7.456962	1.165833
C	2.044889	6.377382	-1.228870
H	0.995390	6.705893	-1.219782
H	2.673902	7.273446	-1.336634
H	2.195323	5.755415	-2.123225
C	3.889915	5.208643	-0.015660
H	4.538486	6.091131	-0.129680
H	4.201789	4.681165	0.897518
H	4.075629	4.539875	-0.869367
N	0.112870	0.113133	1.439767
N	0.554332	1.345989	3.289141
C	0.693320	2.570767	4.063950
H	0.341512	3.432643	3.491369
H	0.078263	2.480664	4.968500
H	1.735990	2.742306	4.368753
C	0.455843	0.112158	4.059779
H	1.008933	-0.692877	3.563091
H	0.889138	0.284518	5.050840
H	-0.590105	-0.207762	4.180039
C	-2.722071	0.162571	-0.913727
O	-3.354645	0.654861	-1.822988
O	-3.158277	-0.897694	-0.223661
C	-1.834420	2.735889	-1.750887
O	-1.490003	2.807508	-2.902969
O	-2.856939	3.394869	-1.216839
C	-3.683884	4.109301	-2.135171
H	-3.113445	4.893680	-2.650164
H	-4.111378	3.424802	-2.879395
H	-4.484432	4.561881	-1.541344
C	-4.319890	-1.547984	-0.732371
H	-4.490172	-2.418286	-0.090344
H	-5.192844	-0.882792	-0.697496
H	-4.160077	-1.875805	-1.768824
C	-0.775640	-2.094753	-1.266930
C	-0.003124	-2.563173	0.903231
C	-0.892990	-3.481211	-1.104459
C	-0.909673	-1.501059	-2.520721

C	-0.636279	-3.842029	0.336784
H	1.095975	-2.622941	0.912844
H	-0.337004	-2.318100	1.916696
C	-1.202720	-4.288183	-2.189868
C	-1.223563	-2.325210	-3.606046
H	-0.772370	-0.431984	-2.668913
H	0.021025	-4.712989	0.446853
C	-1.378713	-3.702082	-3.448402
H	-1.300014	-5.367975	-2.062345
H	-1.337135	-1.876037	-4.594229
H	-1.620986	-4.325281	-4.310638
H	-1.583998	-4.074200	0.846743
N	-0.441024	-1.507588	-0.025048

16
DMAD, G = -533.3693582 a.u.

C	-0.428354	0.565699	-0.735656
C	0.710448	0.898334	-0.504190
C	2.064496	1.372495	-0.205570
O	2.351197	2.538227	-0.127277
O	2.896474	0.355214	-0.039084
C	-1.783751	0.131777	-1.085036
O	-2.147405	-0.041519	-2.218612
O	-2.521938	-0.037792	0.001805
C	4.257162	0.698079	0.260371
H	4.691346	1.296005	-0.551037
H	4.314609	1.261197	1.200666
H	4.793348	-0.250496	0.356883
C	-3.873143	-0.468222	-0.216752
H	-4.326125	-0.555690	0.775128
H	-4.420068	0.269717	-0.817494
H	-3.890257	-1.440092	-0.726427

63
F1_conf1, G = -1399.526103 a.u.

C	-0.884823	0.717017	0.279840
C	-2.103967	0.183731	-0.185370
H	-2.769745	0.681178	-0.891606
C	-2.281957	-1.112595	0.342502
C	-0.282003	-0.271702	1.135426
C	-1.164290	-1.383453	1.148684
H	-1.009266	-2.284826	1.740782
C	-0.450571	2.060268	-0.086649
N	0.818671	2.348656	-0.402165
N	-1.335321	3.053888	-0.196720
C	-2.509290	3.078577	0.405688
N	-2.746149	2.860183	1.697359
O	-3.580259	3.451300	-0.278147
C	-3.459124	3.607182	-1.695452
H	-4.467284	3.838812	-2.053483
H	-2.776266	4.430916	-1.936582
H	-3.099335	2.683334	-2.165300
C	-3.477342	-2.014196	0.070038
C	-3.352988	-3.331754	0.841679
H	-4.220502	-3.978564	0.641696
H	-2.449650	-3.887785	0.550873
H	-3.307636	-3.162066	1.927458
C	-3.563079	-2.324915	-1.433322
H	-3.676945	-1.405419	-2.026040
H	-2.654566	-2.837692	-1.783340
H	-4.424665	-2.974199	-1.655562
C	-4.768335	-1.301641	0.506651
H	-4.906042	-0.355009	-0.036417
H	-5.653145	-1.928798	0.314136
H	-4.746692	-1.068715	1.582008
C	0.878389	-0.185698	1.994442
C	1.529487	0.940015	2.382651
H	1.192517	1.922436	2.062151
C	1.397427	-1.531779	2.447137
O	2.093081	-2.241724	1.766447
O	0.918292	-1.871280	3.643968
C	1.380440	-3.114939	4.164917
H	0.896303	-3.242238	5.138795
H	1.107434	-3.947821	3.502889
H	2.471181	-3.099042	4.290713
C	2.725726	0.905599	3.226616
O	3.193831	2.146129	3.458087
O	3.262948	-0.085688	3.679557
C	4.358928	2.233394	4.270648
H	4.176919	1.820324	5.272546
H	5.201861	1.694585	3.816187
H	4.602731	3.298298	4.350412
C	1.213317	3.731942	-0.616004
H	2.297403	3.814283	-0.472892
H	0.964436	4.067708	-1.635136
H	0.708821	4.390236	0.098888
C	1.807286	1.353849	-0.782239
H	1.376166	0.350526	-0.748461
H	2.149372	1.560388	-1.807509
H	2.675681	1.391766	-0.110745
C	-4.106477	2.832170	2.221861
H	-4.805895	2.474933	1.460649
H	-4.134456	2.142960	3.074002
H	-4.423655	3.829644	2.561226
C	-1.670378	2.692643	2.661322
H	-1.949477	3.213434	3.586191
H	-1.491445	1.632401	2.885081
H	-0.745816	3.140812	2.283894

63
F1_conf2, G = -1399.52213 a.u.

C	-0.791520	0.530867	-0.108644
C	-1.601967	-0.137859	-1.055920
H	-1.941104	0.294800	-1.997760
C	-2.009146	-1.376032	-0.521806
C	-0.675575	-0.328234	1.035956
C	-1.437582	-1.486755	0.763719
H	-1.487763	-2.355671	1.417569
C	-0.474918	1.949957	-0.171981
N	-0.048191	2.507679	-1.314603
N	-0.590038	2.763141	0.882017
C	-1.408860	2.590591	1.901492
N	-1.039366	2.857372	3.153671
O	-2.692200	2.267346	1.815933
C	-3.392792	2.248919	0.571531
H	-4.422940	2.544423	0.800749
H	-2.962368	2.964446	-0.140557
H	-3.381525	1.241328	0.143735
C	-2.929506	-2.372801	-1.213555
C	-3.044313	-3.664691	-0.397647
H	-3.705661	-4.383409	-0.904658
H	-2.065048	-4.147828	-0.265332
H	-3.463379	-3.476665	0.601579
C	-2.380767	-2.712871	-2.608065
H	-2.302548	-1.815249	-3.238622
H	-1.378156	-3.161518	-2.540800
H	-3.037103	-3.428411	-3.127925
C	-4.331507	-1.757721	-1.365565
H	-4.299339	-0.832319	-1.959375
H	-5.019675	-2.454767	-1.869870
H	-4.762320	-1.510635	-0.383554
C	0.230301	-0.184299	2.160831
C	1.394584	0.504737	2.129316
H	1.728534	0.978213	1.207280
C	-0.189112	-0.913167	3.412509
O	-0.021736	-2.092081	3.600153
O	-0.853585	-0.109113	4.244129
C	-1.258378	-0.690694	5.481248
H	-1.755657	0.102792	6.048315
H	-1.955391	-1.523486	5.317272
H	-0.386465	-1.056024	6.039563
C	2.257824	0.654488	3.303949
O	3.320665	1.431502	3.033352
O	2.074057	0.157580	4.397328
C	4.232910	1.644274	4.105813
H	3.744883	2.156878	4.946351
H	4.650097	0.693982	4.466060
H	5.036531	2.273357	3.708338

C	0.052276	3.954288	-1.426421
H	-0.019279	4.233603	-2.484524
H	-0.759071	4.439487	-0.874353
H	1.012129	4.321255	-1.029333
C	0.494665	1.751371	-2.431979
H	0.675536	0.714071	-2.138749
H	-0.187286	1.764097	-3.294452
H	1.448069	2.204650	-2.738890
C	-2.020949	2.853599	4.230400
H	-2.599854	1.924743	4.228242
H	-1.485333	2.931880	5.181732
H	-2.711799	3.705288	4.145317
C	0.312307	3.270337	3.484749
H	0.313009	4.316401	3.826820
H	0.700779	2.637492	4.292458
H	0.955104	3.183681	2.606692

63

F1_rotation_TS, G = -1399.518493 a.u.

C	-0.884789	0.619495	0.013585
C	-1.928360	0.037679	-0.710906
H	-2.462597	0.510243	-1.535388
C	-2.174618	-1.270032	-0.195376
C	-0.453352	-0.320392	1.004927
C	-1.280247	-1.476957	0.855263
H	-1.181539	-2.384789	1.448912
C	-0.440325	2.030513	-0.113136
N	0.551132	2.358965	-0.947637
N	-0.993114	2.993830	0.595028
C	-2.050505	2.803108	1.386839
N	-1.968902	2.612924	2.692221
O	-3.283600	2.913670	0.932694
C	-3.503359	3.263198	-0.439864
H	-4.584144	3.406276	-0.533679
H	-2.978228	4.193969	-0.686678
H	-3.180997	2.459753	-1.109272
C	-3.234315	-2.227049	-0.722097
C	-3.216777	-3.543585	0.061077
H	-3.987078	-4.230572	-0.320815
H	-2.245246	-4.051891	-0.025018
H	-3.416649	-3.381082	1.130365
C	-2.972415	-2.529643	-2.206723
H	-2.994980	-1.612052	-2.812702
H	-1.986373	-2.997753	-2.346755
H	-3.732925	-3.215521	-2.612471
C	-4.625205	-1.585230	-0.583122
H	-4.690374	-0.644694	-1.149634
H	-5.412605	-2.257093	-0.960139
H	-4.851164	-1.356545	0.469383
C	0.614724	-0.178469	1.956832
C	1.604084	0.755858	1.931293
H	1.686806	1.462229	1.109296
C	0.575916	-1.186565	3.080917
O	1.055719	-2.290022	3.028492
O	-0.148566	-0.732854	4.108199
C	-0.211378	-1.583156	5.250651
H	-0.820008	-1.057806	5.993949
H	-0.678542	-2.545320	5.001395
H	0.794026	-1.764242	5.652980
C	2.557309	0.916472	3.024400
O	3.380456	1.961906	2.808383
O	2.633933	0.228997	4.025129
C	4.350857	2.212612	3.817780
H	3.874236	2.428286	4.784164
H	5.025973	1.354643	3.943180
H	4.924138	3.085880	3.487892
C	0.976344	3.746656	-1.033055
H	1.699252	3.849073	-1.848962
H	0.119710	4.404357	-1.228852
H	1.448536	4.072962	-0.093682
C	1.302901	1.381854	-1.717727
H	0.921025	0.376170	-1.522985
H	1.213450	1.594876	-2.792495
H	2.366581	1.419027	-1.441536
C	-3.174144	2.496147	3.508137
H	-3.939009	1.912223	2.987539
H	-2.907702	1.980516	4.436792
H	-3.580897	3.488168	3.752961
C	-0.700026	2.661222	3.408680
H	-0.786593	3.399500	4.217777
H	-0.465563	1.679323	3.836366
H	0.101601	2.963033	2.731320

57

I, G = -1283.234882 a.u.

C	-2.151755	-1.056008	-0.311419
C	-0.343481	-0.288142	0.605399
C	-1.084729	0.890549	0.203984
C	-2.243493	0.393728	-0.435848
C	-1.022197	2.283194	-0.003016
C	-2.158191	2.643794	-0.768697
C	-2.910137	1.468880	-1.049583
H	-3.825099	1.431117	-1.639562
C	-2.533120	4.045838	-1.234548
C	-2.597467	4.077119	-2.770676
H	-1.635833	3.778766	-3.211512
H	-3.364723	3.387620	-3.152399
H	-2.843649	5.085826	-3.138518
C	-1.495586	5.068941	-0.759884
H	-1.748252	6.074587	-1.128385
H	-1.451278	5.118553	0.338054
H	-0.487284	4.822370	-1.120980
C	-3.908480	4.437251	-0.670096
H	-4.690565	3.739194	-1.002815
H	-3.900923	4.430494	0.430287
H	-4.199909	5.447209	-0.999668
N	-1.011584	-1.439055	0.314584
H	-0.250422	2.964686	0.343840
N	0.871225	-0.297686	1.133447
N	-3.035171	-1.935199	-0.760084
C	-2.804058	-3.366721	-0.670994
H	-3.719167	-3.865431	-0.324887
H	-2.529758	-3.772782	-1.655468
H	-1.995605	-3.561503	0.037324
C	-4.190855	-1.484058	-1.515144
H	-4.666123	-0.629590	-1.018480
H	-3.910055	-1.192036	-2.538505
H	-4.920330	-2.299325	-1.571854
C	1.578721	-1.531764	1.431314
H	1.878079	-1.545664	2.488926
H	0.924703	-2.382842	1.229933
H	2.482211	-1.610593	0.810170
C	1.561753	0.960978	1.367830
H	0.968137	1.616602	2.019561
H	2.517447	0.755832	1.860978
H	1.755901	1.488818	0.422619
C	0.593032	-0.044158	-2.515496
C	0.836123	1.137466	-2.580853
C	1.203772	2.554095	-2.601473
O	1.798734	3.098349	-1.706076
O	0.821345	3.130361	-3.732747
C	0.277954	-1.474976	-2.457340
O	-0.722548	-1.955223	-2.924536
O	1.251864	-2.139365	-1.853928
C	1.169567	4.513370	-3.890441
H	0.688946	5.125988	-3.117850
H	2.258225	4.642093	-3.836915
H	0.804461	4.804405	-4.879745
C	1.048088	-3.548119	-1.698017
H	0.143997	-3.738001	-1.108320
H	0.957250	-4.034577	-2.677890
H	1.930367	-3.927115	-1.174267

57

I-2, G = -1283.240692 a.u.

C	-1.981894	-0.839359	-0.603681
C	0.197281	-0.308730	-0.909607
C	-0.640827	1.095271	-1.073671

C	-1.975409	0.641148	-0.589846
C	-0.483343	2.441637	-0.453215
C	-1.610947	2.757524	0.234439
C	-2.521526	1.596215	0.197256
H	-3.417802	1.498619	0.808890
C	-1.940538	4.045986	0.960639
C	-3.213941	4.644603	0.341682
H	-3.486355	5.582597	0.848349
H	-3.067868	4.863685	-0.726432
H	-4.067725	3.957004	0.430788
C	-0.792887	5.050755	0.835910
H	-1.040836	5.979012	1.370995
H	0.138266	4.654951	1.266681
H	-0.596458	5.312513	-0.213762
C	-2.186931	3.739642	2.446916
H	-3.021596	3.037590	2.585264
H	-1.293425	3.299715	2.914310
H	-2.435333	4.662217	2.992759
N	-0.785607	-1.351027	-0.677564
H	0.349161	3.109547	-0.665713
N	1.326975	-0.397526	-0.049926
N	-3.097836	-1.598280	-0.539194
C	-2.951463	-3.036228	-0.433382
H	-2.693804	-3.351268	0.592651
H	-3.894496	-3.517621	-0.719439
H	-2.153920	-3.382126	-1.101319
C	-4.398057	-1.023302	-0.258565
H	-4.481141	-0.026394	-0.705212
H	-5.175794	-1.656650	-0.704361
H	-4.598936	-0.944922	0.823313
C	0.976521	-0.294474	1.353608
H	1.859154	-0.511298	1.970983
H	0.606980	0.713478	1.629281
H	0.197660	-1.026394	1.600394
C	2.442022	0.450836	-0.419783
H	2.756293	0.237949	-1.449490
H	2.220023	1.532622	-0.334423
H	3.292987	0.234826	0.241089
C	0.439074	-0.186628	-2.411814
C	-0.283254	0.933551	-2.536978
C	-0.690906	1.744465	-3.704226
O	-1.554991	2.586940	-3.678008
O	0.024821	1.429859	-4.785922
C	1.102008	-1.113677	-3.366326
O	2.276808	-1.389628	-3.319482
O	0.247242	-1.609244	-4.254046
C	-0.308222	2.122027	-5.989781
H	-1.353023	1.935207	-6.271620
H	-0.156143	3.203319	-5.873273
H	0.361342	1.732810	-6.763184
C	0.799197	-2.490633	-5.235956
H	-0.036314	-2.805335	-5.868814
H	1.552699	-1.970670	-5.842149
H	1.260045	-3.366338	-4.760611

57

I-6, G = -1283.233566 a.u.

C	-1.943723	-0.571161	-0.895001
C	0.122004	0.528114	-0.332763
C	-0.982726	1.506900	-0.593620
C	-2.202702	0.801963	-0.868541
C	-1.241727	2.857878	-0.617635
C	-2.653304	3.025034	-0.906799
C	-3.242455	1.776507	-1.050800
H	-4.287922	1.580090	-1.272120
C	-3.334442	4.380350	-1.021709
C	-4.825563	4.222041	-1.332724
H	-4.986959	3.695110	-2.284352
H	-5.346353	3.660504	-0.543559
H	-5.306868	5.208157	-1.412516
C	-2.672897	5.191844	-2.147931
H	-3.142607	6.183052	-2.244462
H	-1.601603	5.348257	-1.955639
H	-2.768376	4.676974	-3.115619
C	-3.179678	5.143748	0.304283
H	-3.643176	4.591178	1.135262
H	-2.121270	5.302540	0.556649
H	-3.660409	6.132697	0.245988
N	-0.570463	-0.812188	-0.633156
H	-0.531003	3.666299	-0.452779
N	0.693902	0.553591	0.979987
N	-2.756802	-1.586815	-1.162865
C	-2.421538	-2.976593	-0.883953
H	-3.176693	-3.397483	-0.204725
H	-2.415577	-3.569808	-1.808685
H	-1.440249	-3.038802	-0.408630
C	-4.129841	-1.308078	-1.552105
H	-4.741937	-1.020610	-0.683254
H	-4.159138	-0.501048	-2.293542
H	-4.559327	-2.208898	-2.003774
C	1.711556	-0.457702	1.201050
H	1.928620	-0.524123	2.276080
H	1.351602	-1.440614	0.872033
H	2.665230	-0.243085	0.679596
C	1.091612	1.858168	1.480616
H	0.247569	2.555831	1.462345
H	1.408192	1.748985	2.527095
H	1.930594	2.307162	0.917653
C	0.317443	-0.943779	-1.755951
C	1.017896	0.178008	-1.540702
C	2.238746	0.738665	-2.133704
O	2.930215	0.179313	-2.950404
O	2.485345	1.960571	-1.650006
C	0.358584	-2.028484	-2.778867
O	0.394872	-1.831043	-3.964445
O	0.361358	-3.223457	-2.198118
C	3.655641	2.611080	-2.148041
H	4.555939	2.026932	-1.915588
H	3.590129	2.752548	-3.234889
H	3.702579	3.583229	-1.647691
C	0.359638	-4.357033	-3.073324
H	-0.483194	-4.305765	-3.774080
H	1.298530	-4.406202	-3.640032
H	0.263759	-5.237469	-2.430989

57

II, G = -1283.223574 a.u.

C	-2.003025	-0.859194	-0.847909
C	-0.205538	-0.089384	0.123057
C	-0.529093	1.002194	-0.883099
C	-1.913233	0.542126	-1.259688
C	-0.786954	2.438547	-0.493632
C	-2.051460	2.785711	-0.830036
C	-2.780897	1.572463	-1.257175
H	-3.858858	1.509363	-1.403237
C	-2.682976	4.161520	-0.787481
C	-3.106626	4.535366	-2.217266
H	-2.239156	4.552778	-2.893673
H	-3.836411	3.819264	-2.622785
H	-3.572449	5.531932	-2.232363
C	-1.689120	5.199780	-0.261979
H	-2.158940	6.193545	-0.235383
H	-1.355954	4.959962	0.758122
H	-0.798773	5.267136	-0.903474
C	-3.918872	4.127850	0.125327
H	-4.669197	3.407023	-0.229671
H	-3.644266	3.853082	1.154361
H	-4.398133	5.117585	0.154703
N	-1.026583	-1.147656	0.036928
H	-0.010333	3.123820	-0.172061
N	0.830271	-0.071826	0.930777
N	-2.915398	-1.729975	-1.206912
C	-2.898385	-3.088630	-0.686262
H	-3.928224	-3.460782	-0.630334
H	-2.312693	-3.732435	-1.357561
H	-2.451788	-3.103464	0.311479
C	-3.867859	-1.449630	-2.268778
H	-4.879094	-1.315951	-1.859494

H	-3.573586	-0.550286	-2.817649
H	-3.878385	-2.295425	-2.968231
C	1.114854	-1.186973	1.822812
H	1.364575	-0.795875	2.817621
H	0.242825	-1.840619	1.894533
H	1.971882	-1.760223	1.441818
C	1.778460	1.032359	0.963590
H	1.574773	1.688881	1.822022
H	2.789628	0.620125	1.072383
H	1.745782	1.616476	0.039141
C	0.806969	-0.232120	-2.755918
C	0.434575	0.886117	-2.126269
C	1.017063	2.185608	-2.594149
O	1.665202	2.944160	-1.898459
O	0.793911	2.436521	-3.888427
C	0.454099	-1.580946	-2.429946
O	-0.476241	-2.224615	-2.903626
O	1.376033	-2.171485	-1.610806
C	1.414390	3.606338	-4.415838
H	1.052895	4.510150	-3.906433
H	2.507077	3.556070	-4.313635
H	1.143975	3.647485	-5.476455
C	1.134185	-3.527646	-1.283224
H	0.230344	-3.637560	-0.666949
H	1.020060	-4.150100	-2.182071
H	2.003944	-3.874255	-0.711905

57

III, G = -1283.22708 a.u.

C	-2.084179	-0.915263	-0.225275
C	0.000257	-0.400797	0.122863
C	-0.497129	0.790971	-0.687446
C	-1.972298	0.519503	-0.492315
C	-0.384411	2.210238	-0.190522
C	-1.609341	2.698910	0.111968
C	-2.599118	1.609980	-0.013223
H	-3.629124	1.657822	0.339395
C	-1.986210	4.116063	0.488891
C	-2.692443	4.113225	1.853554
H	-2.987962	5.135768	2.131991
H	-3.603447	3.497826	1.841727
H	-2.031249	3.725141	2.642359
C	-2.943129	4.653430	-0.588106
H	-3.233049	5.690727	-0.363002
H	-2.470413	4.630185	-1.579886
H	-3.863432	4.053457	-0.643140
C	-0.746209	5.009720	0.561515
H	-0.027773	4.644020	1.309549
H	-0.229733	5.064451	-0.407481
H	-1.032785	6.032819	0.844899
N	-0.903764	-1.394367	0.208281
H	0.531096	2.794266	-0.257082
N	1.177690	-0.491398	0.692872
N	-3.168750	-1.653555	-0.330310
C	-3.178686	-3.058197	0.052288
H	-3.929112	-3.219587	0.838798
H	-3.442565	-3.675533	-0.817470
H	-2.192259	-3.348024	0.421200
C	-4.435268	-1.098912	-0.783908
H	-5.104676	-0.914085	0.068483
H	-4.269429	-0.163671	-1.327351
H	-4.916864	-1.817727	-1.458800
C	1.540794	-1.668187	1.472194
H	2.055979	-2.400420	0.832828
H	2.214715	-1.362450	2.281018
H	0.645537	-2.129772	1.896982
C	2.221920	0.504510	0.501282
H	2.289263	1.167640	1.375546
H	3.180464	-0.014252	0.376293
H	2.037554	1.089530	-0.401812
C	0.829369	-0.410349	-2.468698
C	0.011047	0.607041	-2.143962
C	-0.578405	1.576909	-3.116359
O	-1.616182	2.186345	-2.967171
O	0.161096	1.706740	-4.230212
C	1.296594	-0.624819	-3.819277
O	2.414703	-0.393085	-4.245157
O	0.375335	-1.288427	-4.579294
C	-0.391036	2.510041	-5.263495
H	-0.536337	3.546455	-4.928581
H	0.327602	2.487498	-6.090050
H	-1.357271	2.112134	-5.604126
C	0.799953	-1.644209	-5.882288
H	-0.029375	-2.193123	-6.345297
H	1.030559	-0.758267	-6.492372
H	1.692841	-2.286431	-5.861877

57

IV, G = -1283.237193 a.u.

C	-2.045758	-0.728865	-0.487555
C	0.131480	-0.181711	-0.739699
C	-0.682901	1.182949	-0.956735
C	-2.043117	0.749970	-0.505731
C	-0.548343	2.551885	-0.369065
C	-1.706874	2.897681	0.242082
C	-2.623365	1.735073	0.210667
H	-3.552971	1.670290	0.774738
C	-2.080640	4.225974	0.866725
C	-3.295469	4.788507	0.110446
H	-3.599161	5.757706	0.533603
H	-3.061400	4.938413	-0.953893
H	-4.159543	4.110896	0.173070
C	-0.919266	5.218272	0.775387
H	-1.200219	6.174715	1.239692
H	-0.026180	4.842955	1.295661
H	-0.642924	5.422695	-0.268946
C	-2.448609	4.008216	2.343072
H	-3.298107	3.319423	2.454816
H	-1.599609	3.593672	2.906387
H	-2.732174	4.962811	2.810872
N	-0.846493	-1.233396	-0.538585
H	0.288902	3.217244	-0.569854
N	1.195714	-0.210229	0.219078
N	-3.158490	-1.492603	-0.428513
C	-3.004217	-2.928068	-0.301116
H	-2.737687	-3.226712	0.727440
H	-3.946835	-3.417774	-0.573408
H	-2.210293	-3.281450	-0.969934
C	-4.465328	-0.917568	-0.179802
H	-4.552805	0.060939	-0.665018
H	-5.234088	-1.573193	-0.608050
H	-4.676464	-0.799985	0.896522
C	1.709189	-1.545773	0.488149
H	2.201563	-2.005586	-0.391537
H	2.454102	-1.485057	1.293669
H	0.900166	-2.208535	0.810669
C	2.271694	0.714595	-0.089063
H	2.824384	0.436540	-1.007751
H	1.890573	1.733835	-0.218499
H	2.987166	0.732360	0.744521
C	0.433423	-0.101719	-2.259980
C	-0.299023	1.007781	-2.417489
C	-0.695904	1.790740	-3.604953
O	-1.530662	2.662992	-3.602434
O	-0.006029	1.410649	-4.684033
C	1.154430	-1.002115	-3.195100
O	2.355751	-1.127752	-3.207132
O	0.320993	-1.664253	-3.990843
C	-0.323426	2.077015	-5.906377
H	-1.376418	1.919509	-6.175499
H	-0.131946	3.155189	-5.824494
H	0.325651	1.640499	-6.671983
C	0.924966	-2.553053	-4.934783
H	0.102052	-3.027235	-5.478536
H	1.564462	-1.998163	-5.633933
H	1.526431	-3.316447	-4.424123

57

TS_(I-6)-6, G = -1283.229648 a.u.

C	-2.008511	-0.654419	-0.636824
C	0.291353	0.658642	-0.170826
C	-0.930255	1.456389	-0.469572
C	-2.161235	0.754477	-0.684403
C	-1.154679	2.809354	-0.638238
C	-2.546767	2.986665	-0.970256
C	-3.160758	1.738566	-0.997527
H	-4.205850	1.552335	-1.227012
C	-3.197045	4.338211	-1.229316
C	-4.679153	4.176439	-1.580305
H	-4.816093	3.564888	-2.483999
H	-5.239495	3.699499	-0.763013
H	-5.138400	5.158258	-1.769545
C	-2.483197	5.038217	-2.397637
H	-2.931653	6.023522	-2.599815
H	-1.416731	5.196312	-2.180923
H	-2.552462	4.440352	-3.318836
C	-3.076533	5.215580	0.027953
H	-3.581904	4.747756	0.886246
H	-2.024792	5.376327	0.306366
H	-3.533373	6.204269	-0.135735
N	-0.680852	-1.012869	-0.411238
H	-0.412400	3.601111	-0.535253
N	0.960500	0.859776	0.972286
N	-2.958283	-1.576965	-0.750381
C	-2.654491	-2.999509	-0.779350
H	-3.334532	-3.531986	-0.101046
H	-2.779539	-3.405061	-1.793339
H	-1.623705	-3.162432	-0.450204
C	-4.343007	-1.185190	-0.950634
H	-4.639260	-0.431055	-0.210533
H	-4.500842	-0.775088	-1.959003
H	-4.981833	-2.065839	-0.827902
C	2.202083	0.163375	1.265315
H	2.046117	-0.561977	2.079092
H	2.568189	-0.377935	0.388951
H	2.970651	0.881059	1.584887
C	0.343132	1.468315	2.139880
H	-0.517004	2.076937	1.851280
H	0.013301	0.690707	2.847770
H	1.076679	2.108313	2.648666
C	0.176076	-1.047184	-1.503423
C	0.990400	0.015243	-1.346681
C	2.112132	0.480172	-2.163315
O	2.488124	-0.058776	-3.180101
O	2.658820	1.596077	-1.662820
C	0.008193	-1.987378	-2.658309
O	-0.938604	-1.926185	-3.404004
O	0.980867	-2.881728	-2.726939
C	3.748093	2.149616	-2.400285
H	4.580709	1.436422	-2.464650
H	3.434412	2.425257	-3.416008
H	4.066317	3.043609	-1.854383
C	0.912679	-3.808214	-3.816960
H	-0.016561	-4.391000	-3.774734
H	0.965732	-3.276932	-4.776081
H	1.776250	-4.471273	-3.708742

57

TS_I-II, G = -1283.207343 a.u.

C	-1.927818	-0.885973	-0.848374
C	-0.267671	-0.072015	0.300429
C	-0.698867	1.046838	-0.540945
C	-1.919480	0.547128	-1.146492
C	-0.902808	2.474907	-0.370209
C	-2.089184	2.812321	-0.989511
C	-2.745512	1.597753	-1.450613
H	-3.734338	1.552125	-1.903543
C	-2.667521	4.203016	-1.184768
C	-4.056365	4.271066	-0.528960
H	-4.748044	3.536774	-0.966434
H	-3.994596	4.073720	0.551595
H	-4.501212	5.268434	-0.666097
C	-2.802303	4.484178	-2.690241
H	-3.202014	5.495285	-2.863059
H	-1.830173	4.401618	-3.195389
H	-3.485154	3.768102	-3.170673
C	-1.763774	5.263390	-0.549675
H	-0.761543	5.265139	-1.000653
H	-2.195183	6.265287	-0.690533
H	-1.647108	5.099532	0.531549
N	-0.946958	-1.208154	0.021449
H	-0.191664	3.171835	0.064521
N	0.748608	-0.053704	1.138199
N	-2.765165	-1.780195	-1.338491
C	-2.746752	-3.167908	-0.903872
H	-2.033727	-3.288593	-0.085616
H	-3.750457	-3.455388	-0.560472
H	-2.462807	-3.821992	-1.739788
C	-3.789562	-1.408827	-2.301264
H	-4.726119	-1.131666	-1.794716
H	-3.449111	-0.571819	-2.919782
H	-3.986879	-2.265570	-2.956592
C	1.126566	-1.230494	1.905985
H	0.281145	-1.921333	1.961205
H	1.969512	-1.737649	1.416622
H	1.413614	-0.921666	2.919118
C	1.582956	1.130547	1.268122
H	2.594856	0.817633	1.550270
H	1.643890	1.662823	0.312004
H	1.196312	1.810046	2.041982
C	1.091068	-0.435808	-2.259636
C	0.551229	0.694280	-2.085355
C	0.527841	2.001358	-2.785589
O	-0.169061	2.261627	-3.734868
O	1.404252	2.855556	-2.247892
C	1.326947	-1.765745	-1.791477
O	2.199767	-2.096610	-1.007522
O	0.521963	-2.668150	-2.401986
C	1.467867	4.150987	-2.844309
H	0.483286	4.634518	-2.846785
H	2.168108	4.734375	-2.237869
H	1.836355	4.086263	-3.877180
C	0.683990	-4.013067	-1.976111
H	0.449069	-4.127088	-0.908453
H	-0.011039	-4.615513	-2.571487
H	1.710450	-4.367322	-2.145887

57

TS_II-III, G = -1283.206408 a.u.

C	-2.172130	-0.780948	-0.413348
C	-0.056315	-0.281272	-0.276104
C	-0.656180	0.979468	-0.891195
C	-2.099425	0.669880	-0.580798
C	-0.507933	2.355833	-0.295930
C	-1.701243	2.798399	0.164564
C	-2.689629	1.711086	0.039503
H	-3.684362	1.717211	0.484461
C	-2.046973	4.171593	0.700799
C	-2.614416	4.035407	2.122416
H	-2.887420	5.023951	2.520733
H	-3.517616	3.408900	2.144483
H	-1.875172	3.588319	2.803337
C	-3.107299	4.791490	-0.223678
H	-3.383299	5.797301	0.126908
H	-2.729830	4.872311	-1.252371
H	-4.023595	4.183433	-0.250301
C	-0.810231	5.072268	0.733356
H	-0.020436	4.651003	1.372177
H	-0.391190	5.220637	-0.272212
H	-1.073123	6.061700	1.134589
N	-0.947694	-1.283947	-0.166930
H	0.386145	2.966067	-0.405545
N	1.201450	-0.458069	0.056867
N	-3.248798	-1.536896	-0.437611
C	-3.148738	-2.957550	-0.121813
H	-4.024394	-3.471808	-0.531111

H	-2.239074	-3.378340	-0.561684
H	-3.119594	-3.110969	0.967060
C	-4.575816	-0.975171	-0.643302
H	-5.106613	-0.868733	0.313863
H	-4.500254	0.003833	-1.126605
H	-5.151958	-1.644403	-1.293602
C	1.647910	-1.772727	0.502977
H	1.838889	-2.423317	-0.364339
H	2.575435	-1.654255	1.073326
H	0.885507	-2.239234	1.133159
C	2.254033	0.491154	-0.280500
H	2.972933	0.533353	0.547150
H	2.760032	0.168400	-1.202448
H	1.842855	1.488842	-0.438222
C	0.552262	0.004820	-2.880532
C	-0.270788	0.920928	-2.412582
C	-0.938555	1.957164	-3.262525
O	-1.887643	2.632546	-2.926862
O	-0.383986	2.066525	-4.478972
C	1.474831	-0.794680	-3.501208
O	2.704065	-0.613981	-3.548415
O	0.916120	-1.932812	-4.062564
C	-0.993773	3.004010	-5.356754
H	-0.961120	4.020854	-4.941045
H	-0.422905	2.970561	-6.291255
H	-2.042144	2.741680	-5.556587
C	1.842774	-2.865038	-4.574593
H	1.256353	-3.701844	-4.976387
H	2.460516	-2.443060	-5.381878
H	2.522733	-3.249005	-3.797529

57

TS_III-IV, G = -1283.211137 a.u.

C	-2.006516	-0.794985	-0.316937
C	0.136233	-0.273244	-0.307163
C	-0.555232	1.027697	-0.808431
C	-1.974654	0.668924	-0.452228
C	-0.435496	2.421317	-0.260078
C	-1.636969	2.838399	0.203668
C	-2.592993	1.712247	0.133985
H	-3.584429	1.711244	0.585838
C	-2.023162	4.214334	0.705538
C	-3.139364	4.761312	-0.199071
H	-3.441808	5.767791	0.126737
H	-2.802874	4.824072	-1.243730
H	-4.032241	4.119567	-0.171789
C	-0.824998	5.165536	0.668254
H	-1.116215	6.156873	1.044649
H	0.000934	4.799043	1.294905
H	-0.443668	5.295442	-0.354722
C	-2.538276	4.101050	2.149229
H	-3.411799	3.437240	2.220420
H	-1.758871	3.708124	2.818635
H	-2.841664	5.089421	2.525526
N	-0.786198	-1.286293	-0.155061
H	0.435298	3.056215	-0.409175
N	1.277059	-0.313776	0.406233
N	-3.102141	-1.551190	-0.312653
C	-3.017077	-2.978216	-0.051497
H	-3.301722	-3.202854	0.988516
H	-3.701041	-3.512834	-0.723521
H	-1.994185	-3.324035	-0.222563
C	-4.433205	-0.976711	-0.395669
H	-4.402127	-0.008185	-0.904741
H	-5.077668	-1.649284	-0.975973
H	-4.877981	-0.846130	0.603010
C	1.724145	-1.608035	0.895150
H	2.106803	-2.233286	0.068161
H	2.531347	-1.453662	1.620531
H	0.899052	-2.137823	1.379601
C	2.342392	0.603388	0.057871
H	2.832899	0.278919	-0.877853
H	1.960760	1.616538	-0.091579
H	3.082045	0.626572	0.866744
C	0.622258	-0.227622	-2.382970
C	-0.172732	0.845464	-2.270829
C	-0.732892	1.714894	-3.332056
O	-1.550549	2.591002	-3.158702
O	-0.234639	1.416372	-4.541467
C	1.205266	-0.778343	-3.598048
O	2.328980	-0.551528	-4.000634
O	0.394665	-1.685524	-4.184812
C	-0.732483	2.179393	-5.634838
H	-1.820802	2.066543	-5.734129
H	-0.497907	3.245758	-5.512299
H	-0.237424	1.793363	-6.532178
C	0.917190	-2.323426	-5.342397
H	0.159567	-3.044269	-5.670254
H	1.104295	-1.598637	-6.147549
H	1.856032	-2.851302	-5.123219

57

TS_IV-(I-2), G = -1283.227927 a.u.

C	-2.035151	-0.770233	-0.545991
C	0.136202	-0.215492	-0.850799
C	-0.732649	1.163832	-1.122867
C	-2.051221	0.710059	-0.596780
C	-0.576792	2.528205	-0.551726
C	-1.676668	2.844876	0.178268
C	-2.573928	1.670950	0.198710
H	-3.438038	1.567600	0.854622
C	-1.990562	4.139099	0.901151
C	-3.308361	4.704471	0.347501
H	-3.571513	5.642047	0.859747
H	-3.226855	4.916014	-0.728942
H	-4.141691	4.001108	0.490714
C	-0.871466	5.162841	0.697107
H	-1.108502	6.095770	1.229018
H	0.089317	4.792805	1.083319
H	-0.737661	5.408948	-0.366149
C	-2.148018	3.851821	2.403377
H	-2.957301	3.133493	2.597859
H	-1.220411	3.439546	2.827164
H	-2.387674	4.777634	2.947644
N	-0.831123	-1.265337	-0.597407
H	0.242549	3.199136	-0.804211
N	1.229512	-0.199143	0.037500
N	-3.138203	-1.543207	-0.453062
C	-2.969118	-2.973454	-0.290773
H	-2.678951	-3.241499	0.739694
H	-3.913558	-3.477342	-0.528679
H	-2.186501	-3.339939	-0.965913
C	-4.451554	-0.979855	-0.212007
H	-4.531855	0.012565	-0.668609
H	-5.212117	-1.624439	-0.671568
H	-4.679910	-0.894103	0.863725
C	1.280435	-0.973607	1.250099
H	2.119504	-1.694093	1.246811
H	1.397042	-0.338657	2.147421
H	0.355528	-1.548045	1.360730
C	2.391666	0.559071	-0.347325
H	2.991192	0.056335	-1.128474
H	2.119097	1.557131	-0.726776
H	3.036627	0.717130	0.527482
C	0.401727	-0.165860	-2.362718
C	-0.350682	0.924325	-2.566645
C	-0.793704	1.659560	-3.773091
O	-1.821625	2.290837	-3.830344
O	0.080172	1.549378	-4.772693
C	1.079757	-1.157129	-3.233051
O	2.130903	-1.682551	-2.951668
O	0.382536	-1.419198	-4.335988
C	-0.285248	2.176680	-6.002215
H	-1.218315	1.751087	-6.395350
H	-0.414060	3.258750	-5.866636
H	0.534721	1.983740	-6.701087
C	0.971636	-2.346469	-5.249604
H	0.275788	-2.433118	-6.090009

H	1.943408	-1.979090	-5.605344
H	1.110348	-3.327425	-4.776042

57
TS_I-V, G = -1283.204816 a.u.

C	0.533333	1.296094	-0.530308
C	0.446799	-0.846564	-1.037964
C	-0.907873	-0.451289	-0.786452
C	-0.856315	0.926742	-0.451906
C	-2.260718	-0.847055	-0.828401
C	-3.037647	0.290927	-0.507149
C	-2.169492	1.395338	-0.287392
H	-2.484398	2.405181	-0.030637
C	-4.555026	0.350518	-0.384675
C	-4.940147	0.738320	1.052827
H	-4.560793	0.001484	1.776478
H	-4.526872	1.719841	1.327139
H	-6.034192	0.792093	1.166782
C	-5.182923	-1.007329	-0.714984
H	-6.278492	-0.953159	-0.630356
H	-4.943475	-1.326239	-1.739928
H	-4.835223	-1.792441	-0.027817
C	-5.111794	1.405037	-1.354923
H	-4.705128	2.403870	-1.139835
H	-4.858412	1.157345	-2.396637
H	-6.208573	1.468586	-1.279652
N	1.325223	0.210385	-0.846401
H	-2.647368	-1.837258	-1.054768
N	0.867752	-2.052779	-1.352803
N	1.042086	2.494712	-0.322669
C	2.430720	2.820116	-0.612755
H	2.465583	3.771445	-1.159137
H	3.008973	2.931088	0.315734
H	2.867953	2.036030	-1.234553
C	0.179038	3.578037	0.125297
H	-0.416080	3.980522	-0.707881
H	-0.496555	3.226177	0.913454
H	0.802811	4.379600	0.534233
C	2.266548	-2.361803	-1.599144
H	2.342245	-2.984731	-2.499883
H	2.832413	-1.439543	-1.743376
H	2.689505	-2.917195	-0.748890
C	-0.093817	-3.144335	-1.400086
H	-0.817527	-2.993061	-2.213294
H	0.443575	-4.081058	-1.576537
H	-0.625800	-3.214710	-0.442744
C	1.717838	-0.819761	1.691924
C	2.378553	-0.258258	0.779536
C	3.770739	0.185779	0.535969
O	4.496458	0.525236	1.438524
O	4.158764	0.126936	-0.736653
C	0.450799	-1.405178	2.036972
O	0.142919	-2.560902	1.820198
O	-0.343051	-0.533803	2.681107
C	5.501164	0.541478	-0.998739
H	5.654615	1.583622	-0.688693
H	6.218420	-0.100271	-0.470633
H	5.642196	0.448836	-2.080053
C	-1.659986	-0.993397	2.973416
H	-2.160760	-1.355822	2.066420
H	-1.641824	-1.802132	3.717631
H	-2.200972	-0.133004	3.381219

57
TS_V-(I-6), G = -1283.203418 a.u.

C	-2.083242	-0.335314	-0.862182
C	-0.197140	0.692196	-0.018438
C	-1.198572	1.711041	-0.274562
C	-2.360199	1.043036	-0.778854
C	-1.499621	3.055525	-0.106945
C	-2.865953	3.239041	-0.508945
C	-3.399064	2.008436	-0.905689
H	-4.415462	1.830514	-1.245667
C	-3.580230	4.582911	-0.497341
C	-2.854766	5.555494	-1.441972
H	-3.349315	6.539244	-1.446558
H	-1.809629	5.708970	-1.136497
H	-2.850134	5.174584	-2.474168
C	-3.565138	5.160185	0.927616
H	-2.538404	5.304145	1.293857
H	-4.070571	6.137801	0.958582
H	-4.081353	4.490409	1.631576
C	-5.033747	4.435385	-0.957325
H	-5.096879	4.045075	-1.983451
H	-5.598929	3.756334	-0.302482
H	-5.540169	5.411850	-0.941024
N	-0.760874	-0.592716	-0.428249
H	-0.833728	3.844358	0.237495
N	0.692403	0.701946	0.987244
N	-2.858405	-1.307464	-1.306381
C	-2.560463	-2.721399	-1.127152
H	-3.445867	-3.216420	-0.706036
H	-2.319020	-3.193648	-2.089893
H	-1.719051	-2.847906	-0.443237
C	-4.154347	-0.975537	-1.880990
H	-4.068666	-0.098883	-2.533070
H	-4.499733	-1.823894	-2.481231
H	-4.895966	-0.770895	-1.094387
C	1.522097	-0.467675	1.220786
H	2.344305	-0.512831	0.485531
H	1.942024	-0.406628	2.231372
H	0.923768	-1.380796	1.139229
C	1.269855	1.982588	1.354181
H	1.912479	2.373057	0.545968
H	0.482247	2.712318	1.571485
H	1.877564	1.853305	2.256076
C	0.218839	-0.918480	-1.464360
C	0.893062	0.197539	-1.746888
C	2.181816	0.281698	-2.422098
O	3.250453	0.127067	-1.862531
O	2.075181	0.638225	-3.710210
C	0.633798	-2.321043	-1.717769
O	0.480175	-3.242043	-0.948907
O	1.286220	-2.423643	-2.876941
C	3.301669	0.774809	-4.422290
H	3.950483	1.529442	-3.957107
H	3.843917	-0.179995	-4.464312
H	3.036588	1.091137	-5.436930
C	1.889612	-3.687985	-3.152787
H	1.132819	-4.482314	-3.192674
H	2.377522	-3.588458	-4.127493
H	2.635024	-3.939437	-2.386483

57
V, G = -1283.224948 a.u.

C	-1.487556	-0.254856	-1.548213
C	0.224954	0.464636	-0.251715
C	-0.676445	1.553711	-0.400148
C	-1.751904	1.104096	-1.211369
C	-0.983008	2.849067	0.062201
C	-2.242832	3.198153	-0.477228
C	-2.727592	2.114494	-1.254090
H	-3.678677	2.090538	-1.781764
C	-2.975244	4.520830	-0.294491
C	-3.138710	5.205776	-1.661392
H	-2.160101	5.405255	-2.123144
H	-3.715499	4.579067	-2.357008
H	-3.667088	6.166374	-1.558818
C	-2.194671	5.452175	0.638280
H	-2.734157	6.402035	0.767785
H	-2.059608	5.007342	1.634983
H	-1.198896	5.688106	0.235759
C	-4.365294	4.261938	0.308409
H	-4.971404	3.613781	-0.340951
H	-4.286444	3.773416	1.291113
H	-4.915245	5.206117	0.443191
N	-0.272906	-0.652261	-0.955690
H	-0.371879	3.473994	0.707645

N	1.369661	0.458194	0.389230	H	1.748246	2.501625	0.082946
N	-2.210747	-1.039381	-2.311214	C	0.672766	-1.477037	-1.749850
C	-2.039305	-2.484513	-2.414246	C	1.340924	-0.972136	-2.797673
H	-3.026257	-2.927707	-2.588601	C	1.157261	0.432253	-3.093700
H	-1.377144	-2.748747	-3.249535	O	1.873307	1.337142	-2.698109
H	-1.648712	-2.884106	-1.476157	O	0.110335	0.658362	-3.929085
C	-3.314161	-0.454067	-3.065291	C	0.848634	-2.887628	-1.304643
H	-4.217550	-0.379773	-2.442847	O	1.519714	-3.718120	-1.868270
H	-3.037720	0.541466	-3.428151	O	0.173190	-3.171169	-0.168284
H	-3.525557	-1.096024	-3.926580	C	-0.137540	2.019744	-4.257964
C	2.136628	-0.739154	0.711162	H	-0.398550	2.605203	-3.364920
H	2.618814	-0.577696	1.682219	H	0.736619	2.481353	-4.737680
H	1.472953	-1.601554	0.798491	H	-0.981409	2.024028	-4.957032
H	2.909463	-0.928392	-0.046066	C	0.303702	-4.505077	0.316664
C	1.910280	1.733140	0.846575	H	-0.061828	-5.230647	-0.422402
H	1.439173	2.044059	1.790149	H	1.350987	-4.736462	0.553212
H	2.986833	1.616627	1.006658	H	-0.303535	-4.563311	1.226090

S10 References

1 Kläs, H.-G. Beiträge zur Chemie des 2-Azapentalene-Systems. Ph.D. Dissertation (Berichterstatter: Prof. Dr. K. Hafner), *Technische Hochschule Darmstadt*, **1984**.

2 Roger, J.; Royer, S.; Cattey, H.; Savateev, A.; Smaliy, R. V.; Kostyuk, A. N.; Hierso, J.-C. Diastereoselective Synthesis of Dialkylated Bis(phosphino)ferrocenes: Their Use in Promoting Silver-Mediated Nucleophilic Fluorination of Chloroquinolines. *Eur. J. Inorg. Chem.* **2017**, *2*, 330–339.

3 Meiszter, E.; Gazdag, T.; Mayer, P. J.; Kunfi, A.; Holczbauer, T.; Sulyok-Eiler, M.; London, G. Revisiting Hafner's Azapentalenes: The Chemistry of 1,3-Bis(dimethylamino)-2-azapentalene. *J. Org. Chem.* **2024**, *89*, 5941–5951.

4 Neese, F. Software Update: The ORCA Program System—Version 5.0. *Wiley Interdiscip. Rev. Comput. Mol. Sci.* **2022**, *12*, e1606.

5 (a) Najibi, A.; Goerigk, L. DFT-D4 Counterparts of Leading meta-generalized-gradient Approximation and Hybrid Density Functionals for Energetics and Geometries. *J. Comput, Chem.*, **2020**, *41*, 2562–2572. (b) Friede, M.; Ehlert, S.; Grimme, S.; Mewes, J.-M. Do Optimally Tuned Range-Separated Hybrid Functionals Require a Reparametrization of the Dispersion Correction? It Depends. *J. Chem. Theory Comput.* **2023**, *19*, 8097–8107.

6 Weigend, F.; Ahlrichs, R. Balanced Basis Sets of Split Valence, Triple Zeta Valence and Quadruple Zeta Valence Quality for H to Rn: Design and Assessment of Accuracy. *Phys. Chem. Chem. Phys.* **2005**, *7*, 3297–3305.

7 Weigend, F. Accurate Coulomb-Fitting Basis Sets for H to Rn. *Phys. Chem. Chem. Phys.* **2006**, *8*, 1057–1065.

8 Marenich, A. V.; Cramer, C. J.; Truhlar, D. G. Universal Solvation Model Based on Solute Electron Density and on a Continuum Model of the Solvent Defined by the Bulk Dielectric Constant and Atomic Surface Tensions. *J. Phys. Chem. B* **2009**, *113,* 6378–6396.

9 Bannwarth, C.; Ehlert, S.; Grimme, S. GFN2-xTB—An Accurate and Broadly Parametrized Self-Consistent Tight-Binding Quantum Chemical Method with Multipole Electrostatics and Density-Dependent Dispersion Contributions. *J. Chem. Theory Comput.* **2019**, *15*, 1652–1671.

10 Ehlert, S.; Stahn, M.; Spicher, S.; Grimme, S. Robust and Efficient Implicit Solvation Model for Fast Semiempirical Methods. *J. Chem. Theory Comput.* **2021**, *17*, 4250–4261.

11 Casanova-Páez, M.; Goerigk, L. Time-Dependent Long-Range-Corrected Double-Hybrid Density Functionals with Spin-Component and Spin-Opposite Scaling: A Comprehensive Analysis of Singlet–Singlet and Singlet–Triplet Excitation Energies. *J. Chem. Theory Comput.* **2021**, *17*, 5165–5186.

12 Hellweg, A.; Hättig, C.; Höfener, S.; Klopper, W. Optimized Accurate Auxiliary Basis Sets for RI-MP2 and RI-CC2 Calculations for the Atoms Rb to Rn. *Theor. Chem. Acc.* **2007**, *117*, 587–597.

13 Ghosh, S.; Bhattacharyya, K. Origin of the Failure of Density Functional Theories in Predicting Inverted Singlet–Triplet Gaps. *J. Phys. Chem. A* **2022**, *126*, 1378–1385.

14 Peach, M. J. G.; Williamson, M. J.; Tozer, D. J. Influence of Triplet Instabilities in TDDFT. *J. Chem. Theory Comput.* **2011**, *7*, 3578–3585.

15 Lu, T.; Chen, F. Multiwfn: A Multifunctional Wavefunction Analyzer. *J. Comput. Chem.* **2011**, *33*, 580–592.
